# Supplementary material for: The educational pathway to Advanced Practice for the physiotherapist: A systematic mixed studies review
Source: PLoS One. 2025 May 12;20(5):e0322626. doi: 10.1371/journal.pone.0322626 (PMC12068731; doi:10.1371/journal.pone.0322626)
Supplement: S3 Table — (DOCX) [file pone.0322626.s003.docx]

|  |  |  |  |  |
| --- | --- | --- | --- | --- |
| **Study ID** | **Educational Pathway** | **Duration Accreditation  Area of Practice** | **Description of the Education** | **Key Identifiable Components of the Education** |
| Adhikari 2020 | Single Encounter Course | duration: 6 hrs acrd: yes aop: non-specific | The participants were provided with a range of cases or scenarios as a trigger for discussion (Pediatric, MSK, Cardioresp and Neuro). Participants’ active involvement was encouraged and they had to come up with the solution through their critical reasoning during every discussion. A resource person, the first author of this study provided evidence and triggers for discussion through PowerPoint presentations and facilitated participants for discussion in a small group. Each group was asked to discuss within the group first and then to present it to the whole team for further discussion and refine the prescription. This procedure was repeated six times within the six‐hour workshop at various steps (assessment, physiotherapy diagnosis, intervention selection, sequencing of the intervention, parameter selection, and a complete intervention prescription at the end) of the CDM process. The effectiveness of the intervention was assessed before and immediately after the workshop. The first part of the workshop was focused on deriving physiotherapy diagnosis (PTD) (also termed functional diagnosis or functional evaluation) based on the findings from subjective as well as objective examination. The second part of the workshop was focused on developing skills on Exercise Prescription(EP) through clinical reasoning. The protocol adopted for EP was based on an updated and dynamic principle of EP termed FITT‐CORRECT principle, derived by Adhikari et al., 2020 | Lecture/Didactic Approach Case Study Approach Student Assessment |
| Allison 2023 | Single Encounter Course | duration: 8 hours acrd: No aop: MSK | The program included six modules: (1) Overweight and obesity and OA; (2) Weight regulation, overweight and obesity; (3) Examining weight stigma and personal beliefs about weight; (4) Communication approaches for addressing weight management; (5) Interventions for weight management and (6) Health Behaviour Change support. Blended learning modalities including quizzes, lectures, case studies and expert responses were incorporated in line with adult learning principles. Participants randomized to the intervention group were given access to self-directed online learning to complete approximately 8 hours of online education. Participants were required to work through each module and complete a short ‘check your understanding’ quiz at the end of each module, to help reinforce knowledge and learning, before being granted access to the subsequent module. | Lecture/Didactic Approach Case Study Approach Student Assessment |
| Anderseck 2020 | Accredited Area of Practice Training | duration: 260-1000 hrs acrd: yes  aop: MSK | Orthopedic manual therapy is a neuromusculoskeletal specialization of physiotherapy and is associated with constant time-consuming and cost-intensive further training.   The abbreviation OMT (Orthopedic Manual Therapy) is used in this article, although the abbreviation OMPT (Orthopedic Manual Physical Therapy) has become the standard name internationally in recent decades through IFOMPT because this is a specialized neuromusculoskeletal therapy physiotherapists  Certificate training The certificate training is a minimum of 260 hours of advanced training, the scope and procedure of which have been agreed between the professional associations in physiotherapy and the leading associations of the statutory health insurance funds. The duration of the training is usually 2 years and ends with the certificate examination in manual therapy. After passing the examination, the therapists are entitled to examine or treat statutory health insurance patients with a prescription for manual therapy and to bill this at a separate fee rate. However, this is usually only a few euros more than the rate for normal physiotherapy without additional qualifications.  OMT training The OMT training builds on the certificate training. The content and scope of the OMT training corresponds to the requirements of the IFOMPT (International Federation of Orthopaedic Manipulative Physical Therapists). It is the manual therapy subgroup of the WCPT (World Confederation for Physical Therapy). These requirements regulate skills for examining and treating the neuromusculoskeletal system, which go far beyond the scope of the certificate training. With the addition of manipulative techniques and comprehensive supervision in clinical practice on patients, participants learn additional skills in examining and treating these patients. In addition, skills from the fields of science and research are taught, which are essential for professional and evidence-based physiotherapy today. The OMT training comprises around 1000 hours and generally lasts 3 years. It does not entitle you to a special billing position in the statutory health insurance sector. | Skill practice student assessment |
| Balogun 2018 | Single Encounter Course | duration: 3 hours acrd: no aop: non-specific | The professionalism educational intervention implemented in this study consisted of two parts: lecture and discussion (case studies) components. The lecture was presented by the principal investigator who is a distinguished university professor with over 30 years of physical therapy administrative and teaching experience in various universities around the world. The content of the lecture was developed by the principal investigator from different sources in the literature. The highlights of the lecture are presented in Appendix 1. The two-hour classroom lecture was followed by a one-hour question-and-answer session and presentation of case studies on professionalism. The 5 case studies about professionalism were adapted from the literature. The case studies are presented in Appendix 2. The co-investigators actively participated as "facilitators" in the discussion of the case studies. They were also responsible for administering the Professional Inventory before and after the workshop. The principal investigator met the study participants for the first time on the day of the workshop. Two of the 3 co-investigators knew many of the study participants. The educational intervention covered all the professional knowledge questions asked in the Professionalism Inventory. | Lecture/Didactic Approach; Case Study Approach |
| Banks 2013 | Accredited Area of Practice Training | duration: 21 hrs in modules (3x7hrs) + 10 hrs mentorship (1hr x 10 months) acrd: yes  aop: MSK | Three in-service education modules, each lasting 1 day (7 hours) The content of each module was based on subjects identified from analysis of clinical practice.  Determined from sessions of approximately 45 minutes for the participant to examine and treat a new patient whilst being observed by one of seven MACP (Musculoskeletal Association of Chartered Physiotherapists) colleagues. Seven of the 20 competencies were identified as needing to improve, most because their performance ratings during the supervision sessions were the lowest. The 7 competencies were: communication strategies; clinical reasoning; functional analysis of movement; structural differentiation; the range of manual handling skills; the use of research in practice; and critical appraisal of evidence. Each of the competencies matches one of ten dimensions from within the IFOMPT (International Federation of Orthopaedic Manipulative Physiotherapists) educational standards document  Learning outcomes were achieved using a variety of strategies including lectures, self-directed learning workshops, and practical demonstrations. All the sessions were led by clinicians and educators with postgraduate teaching experience.  After each module, participants were asked to reflect on what they had learned by producing three or four further learning outcomes. Between modules, participants were asked to engage in clinical assistance (supervision) with their designated supervisor. Clinical assistance usually takes the form of a 1-hour session every month. Participants received feedback on their performance during a further supervision session (post-test). This supervision session followed the same format as that carried out prior to the start of the in-service education programme.  Learning Outcomes 9.00–10.40 MASTERCLASS Pain mechanisms and their clinical features [pain quiz and Analyse the patient experience of their symptoms and deficits in lecture/workshop] relation to knowledge of the theory of pain mechanisms. 11.00–12.30 FOCUS ON CLINICAL REASONING Clinical reasoning models in practice [patient demonstration Apply a range of clinical reasoning models to clinical practice and forum] 13.00–14.40 RESEARCH TOPIC The use of research in clinical practice [problem solving Reflect upon strategies for embedding knowledge and research activity] within clinical practice 15.00–17.00 CLINICAL SKILLS WORKSHOP Analysis and differentiation of functional demonstration Apply knowledge of the functioning scie | Lecture/Didactic Approach Mentorship Case Study Approach Self Reflection Skill Practice |
| Barton 2021 | Single Encounter Course | duration: 2 days  acrd: no aop: MSK | involved (i) pre- and post-workshop surveys evaluating knowledge and learning needs, (ii) lectures regarding osteoarthritis management, (iii) patient education and exercise-therapy skills training sessions, (iv) training to contribute patient outcomes to the national registry, and (v) discussions about overcoming barriers to implementation. Each trained physiotherapist received an implementation manual, ready-to-use patient education materials (PowerPoint presentations and printable booklets), and access to online implementation resources (e.g. flyers, letter templates for referring doctors). | Lecture/Didactic Approach Case Study Approach Skill Practice |
| Bastick 2020 | Multiple Encounter Course | duration: 8 weeks  acrd: no aop: non-specific | Streams could provide education and training using any method, given the lack of strong evidence demonstrating superiority of any training method over another (Milanese et al, 2014). Education and training was delivered using many and varied educational methods, including the provision of pre-reading, didactic lecture or tutorial format, practical skills training (in non-clinical/lab setting) and ward-based training (including bedside tutorials, practical experience with patients). Six clinical streams (pediatrics, cardiothoracic, critical care, women’s health, orthopedics and neuro-medicine) delivered eight-week training programs (run concurrently with small numbers of therapists, e.g., three to four staff, attending each program). Senior physiotherapists in each clinical stream provided the training (variable years of clinical and educational experience across streams). Primarily face to face small-group intervention, incorporating varied modalities. The intervention was delivered over a 32-week period (four rotations of 8 weeks), with each stream running a single weekly small-group session of 1-h duration. Staff were allocated to stream-specific training programs in a pragmatic ad hoc manner (i.e., three-four staff per stream). Intervention adherence was facilitated by the support of management recommending that junior staff attendance at education sessions be mandatory; however, no other specific strategies were used to maintain fidelity. Participants completed an average of three of four available stream-specific programs in the study period. Formal program attendance (i.e., specific number of sessions attended) was not measured during this study. | Lecture/Didactic Approach Mentorship Skill Practice |
| Bird 2022 | Single Encounter Course | duration: 5 hours acrd: no aop: non-specific | The workshop training program consisted of two modules: the first delivered face-to-face; and the second was delivered by two videoconferences. Module One: Introduction to health literacy workshop incorporated the vignettes from client data as the basis for activities including the co-design of health literacy solutions. Module Two: Health literacy skills workshops delivered over two shorter lunchtime sessions negotiated with individual practices, focusing on communication, designing resources, and workplace audit tools (a total of 5-hours face-to-face learning). Both the content and the timing of these were flexible, with the participants involved in the first module able to choose which health literacy skills they would like to focus on in the second module delivery (e.g. verbal, written, and assessing the organization) and the practice integrating these into their usual professional development activities. | Lecture/Didactic Approach Case Study Approach Skill Practice |
| Brennan 2006 | Multiple Encounter Course | duration: 2 days + 6 month follow up acrd: no aop: MSK | A 2-day CE course focusing on manual therapy of the spine and corresponding exercise interventions supported by evidence was conducted for physical therapists at the participating clinics during October 2003. Two experienced educators and physical therapist clinicians with Fellowship status in the American Academy of Orthopedic Manual Therapy taught the course. The course included lecture and interactive hands-on practice sessions, with the instructors in approximately equal proportions. The evidence for manual therapy interventions for patients with neck pain was reviewed. The ongoing clinical improvement project focused on standardizing examination procedures and tracking clinical outcomes, instead of on treatment protocols or algorithms. Therapists who participated in this project met monthly following the 2-day CE course, providing opportunities for them to discuss their experiences working with patients with neck pain with other therapists and to discuss barriers to standardization. Clinical outcome data, including disability scores, visits, and costs, were presented at these meetings to track progress and reinforce the practice behavior changes identified as favorable to promote better outcomes. “Clinician facilitators” also were available to discuss cervical treatment techniques, evidence-based literature, and the evaluation process. In addition, a follow-up, hands-on review session was conducted approximately 6 months after the original CE course to review hands-on evaluation and treatment skills. | Lecture/Didactic Approach Mentorship Skill Practice |
| Briggs 2023 | Residency and Fellowship | duration: not reported acrd: yes aop: non-specific | Residency education is focused mentored training in a specialty area of practice, and fellowship education is focused mentored training in a subspecialty area of practice. Furthermore, professional and postprofessional education (e.g., residency and fellowship training) fall along a continuum of progression and depth of learning, knowledge, and skills.  one-on-one mentoring is a requirement in accredited residency and fellowship programs, Mentoring and reflection on experiences are expected to facilitate residents’ development and enhance their clinical reasoning skills. | Mentorship Self Reflection |
| Camden 2015 | Single Encounter Course | duration: not reported acrd: no aop: pediatric | The module is comprised of five sections: 1) Identification; 2) Planning Interventions and Goals; 3) Evidence-Based Practice; 4) Management; and 5) Resources. Each of these five main sections is further divided into subsections, each consisting of five distinct elements including: 1) general information, key messages, definitions, explanations, and examples; 2) a case scenario “What About Max?” providing evidence-based clinical applications; 3) links to additional resources (e.g., downloadable one-page evidence-based flyers; web pages; or documents external to the module which might be of relevance); 4) videos demonstrating strategies and/or interventions; and 5) “questions for reflection” to challenge the user’s understanding of the information presented. | Lecture/Didactic Approach Case Study Approach Self Reflection |
| Carr 2020 | Mentorship | duration: not reported acrd: no aop: MSK | regular observed practice with formal graded feedback, an established approach to their practice-based education programme registered interest to partake in this study. The selected organisation was an independent healthcare provider with a large team of physiotherapists working across three community sites, predominantly delivering MSK physiotherapy to NHS patients. Two precursory requirements were identified to achieve a valued practice-based collaborative learning cycle. Both focused on reflective exercises required of a mentor prior to engaging in a learning activity with their learner. | Mentorship Student Assessment |
| Cheema 2022 | Residency and Fellowship | duration: not reported acrd: not reported aop: MSK | Based on survey results, PTs were classified into one of two clinician-training groups: those who were post-professionally trained through either a residency or fellowship, or not residency or fellowship trained. | Mentorship |
| Chipchase 2016 | Single Encounter Course | duration: 2 days acrd: no  aop: MSK | The two-day workshop provided an evidence-based approach towards the diagnosis and management of neck disorders, with an emphasis on multimodal interventions inclusive of advice, education, exercise and manual therapy. The two-day timeframe was selected as it represents common practice and has been utilised by multiple studies exploring the effectiveness of continuing professional development workshops related to the neck and spine (Bekkering et al., 2005; Brennan et al., 2006; Cleland et al.,2009). The course especially promoted a research-informed therapeutic exercise program within the multimodal program that has been shown to be efficacious for persons with neck disorders in clinical trials (Jull et al., 2002, 2007). The workshop was 12.5 h in duration and consisted of lectures (2.5 h), as well as demonstrations, practice and discussion (10 h) over a two-day period. The lead instructor and assistant provided supervision in the practical sessions. | Lecture/Didactic Approach Case Study Approach Skill Practice |
| Cimoli 2012 | Single Encounter Course | duration: 3 hours acrd: no aop: non specific | An EBP professional development workshop was designed specifically for this study. This workshop was presented by two recognised experts in the field of EBP.  Prior to the workshop participants were provided with a package of pre-reading material. This package comprised some EBP background reading prepared by the co-presenters. It also included an article from a peer-reviewed journal related to physiotherapy practice selected in consultation with the two workshop presenters. This article was used to guide group discussion within the critical appraisal component of the workshop program. The workshop was three hours in duration and was delivered via a combination of didactic presentations (90 minutes), group discussion (45 Evidence-Based Practice (EBP) in Rehabilitative Physiotherapy The Internet Journal of Allied Health Sciences and Practice, 2012minutes), and practical skills exercises including database searching (45 minutes). The workshop addressed topics such as formulation of clinical questions, sources of evidence, access and search strategies for EBP, tools and processes for critically appraising research, and the application of EBP into clinical practice. | Lecture/Didactic Approach Case Study Approach Skill Practice |
| Cleland 2009 | Multiple Encounter Course | duration: 8hrs over 2 days (+/- 4hrs over 4-7 weeks) acrd: no  aop: MSK | The CE course was delivered over 2 days (4 hours per day) and focused on the management of neck pain with a previously developed classification system. The CE course included both lectures (approximately 25%) and hands-on practical sessions (approx 75%). The lecture portion included discussion of the current best evidence in support of the classification system for the management of neck pain. The practical sessions consisted of demonstration and practice of manual physical therapy techniques (thrust and nonthrust) directed at the cervical and thoracic spine, as well as therapeutic exercises targeting the deep neck flexor, lower trapezius, middle trapezius, and serratus anterior muscles. Details regarding the exact techniques demonstrated and practiced by the participants can be found elsewhere. One group was randomly assigned to receive ongoing education in the use of the evidence-based interventions, and the second group received no further education organized by IHC beyond the CE course. The additional training included two 1.5-hour educational meetings provided by the same clinicians who delivered the 2-day CE course. These meetings occurred 4 and 7 weeks after the completion of the 2-day course. In addition to the 2 educational meetings, all therapists randomly assigned to receive ongoing education participated in an outreach visit. The outreach visit included a 1-hour co-treatment of a patient with neck pain in the therapist’s own clinical practice setting with the principal investigator of the study. The therapist identified the patient for whom the co-treatment would occur on the basis of patient availability and the consent of the individual patient. After the co-treatment, the therapist and the principal investigator discussed the clinical presentation of the patient. | Lecture/Didactic Approach Mentorship Skill Practice or Practical Application |
| Constantine 2012 | Masters Level Program | Duration: 1-2 yrs  acrd: yes  aop: MSK | "post-graduate courses leading to an academic award, such as a Master of Science (MSc) degree, are highly structured and delivered over a longer time period. the inclusion criteria were expanded to include any graduate of a Masters in manual therapy programme accredited by the International Federation of Orthopaedic Manipulative Physical Therapists.   Modules (1) “Manual therapy concepts” (20 masters or “M”-level credits) (2) “Research methods” (20 “M”-level credits) (3) “Lower quadrant management” which includes a 90-hour clinical placement including assessment of a “live” clinical examination (40 “M”-level credits) (4) “Upper quadrant management” as per lower quadrant module (40 “M”-level credits) (5) Dissertation – empirical- or literature-based research project(60 “M”-level credits)"  It can be seen that the program structure (Table 1) places a considerable emphasis on the development of clinical skills and reasoning in the actual clinical setting. Involvement in this type of learning required that the participants engage in a frequently difficult process of deconstructing their own clinical practice followed by a process of reconstruction.  This was experienced and framed by the participants as personal development.  “One of my main reasons for doing the course was that I was really interested in the clinical aspects of the course, to try to help me change my practice through clinical placements [a requirement of the programme] and so I do feel that the course has met those expectations in that it has changed my clinical practice for the better”.  improving the skills required to undertake research, advance theoretical knowledge, evidence supporting manual therapy are all components of the MSc level education in physiotherapy.  “What it [the programme] did do was lead me to the conclusion that I needed to be more engaged with understanding what was going on and take greater ownership in my own professional development”. | Lecture/Didactic Approach Case Study Approach  Mentorship Skill Practice Student Asssessment Research Assignments |
| Cowell 2019 | Multiple Encounter Course | duration: 10 months acrd: No aop: non-specific | The lead researcher co‐taught the training programme with R.P., a musculoskeletal physiotherapist with 13 years’ experience and a special interest in NSCLBP. Both of these authors had had extensive clinical and teaching experience in CFT. The programme was built on an established teaching programme that had been developed and refined over the previous decade. A multifaceted learning approach was adopted, informed by previous BPS training programmes (Main, Sowden, Hill, Watson, & Hay, 2012) and best evidence guidelines for the management of LBP (Foster et al., 2018; NICE, 2016). Formative pretraining assessments also informed the content and delivery mode of the training programme. This included evaluating the physiotherapists' attitudes and beliefs, as measured by the Pain Attitudes and Beliefs Scale for Physical Therapists (PABS‐PT) (Ostelo, Stomp‐van den Berg, Vlaeyen, Wolters, & de Vet, 2003) and the Attitudes to Back Pain Scale for musculoskeletal practitioners (ABS‐mp) (Pincus et al., 2006). The physiotherapists' perceptions of the challenges of implementing a broad BPS approach for NSCLBP were explored through semistructured interviews, and communication practice was examined using a qualitative data‐driven inductive method to analyse video‐recorded initial encounters with two NSCLBP patients. The initial assessments also provided the lead researcher with an opportunity to observe the physiotherapists' diagnostic, observational and interpretive skills. The 10‐month programme included 3 days of formal educator‐led training, to develop knowledge and awareness of multidimensional causal pain mechanisms for LBP, effective communication practice and targeted interventions for LBP patients using CFT. To develop the physiotherapists' skills and confidence, 17 hr of problem‐based learning (PBL) activities were included, followed by educator‐facilitated group discussions. These educator‐led training and PBL sessions were consolidated by observation of a CFT educator assessing and treating four “live” patients, which provided opportunities for observing and modelling experienced clinicians. A range of teaching methods were employed, including web‐based resources, and e‐book, audio and video training materials. To develop awareness of effective communication practice, the physiotherapists' actual recordings (accompanied by the transcripts) of consultations from the pretraining phase were used as an alternative to traditional role‐play. Six months of clinical mentoring by the educators (I.C., R.P.) allowed for individual clinical observation and feedback in the physiotherapists' own clinical environment, as well as one‐to‐one video review sessions of the baseline recorded assessments. Physiotherapists were sent a summary email after each supervision session, to reinforce key learning points. The training resources and content are detailed in Table 2 | Lecture/Didactic Approach Case Study Approach Mentorship Self Reflection Skill Practice Student Assessment |
| Cunningham 2017 | Residency and Fellowship | duration: 18 months acrd: yes aop: MSK | a clinical residency program is a structured experience for PTs following entry-level education that is designed to advance the therapist’s knowledge, skills, clinical reasoning, and attributes in a specific area of practice. The residency experience combines opportunities for ongoing mentoring to the resident, including required written and practical examinations, with a foundation of evidence-based practice and a needs assessment, interactive education, multiple of instruction, multiple exposures to the material, and case-based learning. In addition, the program emphasizes and provides clinical mentoring within the resident’s current place of employment. active reflection on the outcome of the treatment, as compared to previous experience, is encouraged to facilitate the transfer of knowledge from the program to clinical practice. | Lecture/Didactic Approach Case Study Approach Mentorship Self Reflection Skill Practice Student Assessment |
| Cunningham 2019 | Residency and Fellowship | duration: 18 months  acrd: yes  aop: MSK | Physical therapists in the residency program complete six onsite modules over 18 months. Each module consists of ten days of onsite education provided by physical therapy instructors from the United States. The online didactic portion of the program utilizes the Clinical Practice Guidelines and Current Concepts in Orthopedics, 3rd edition (American Physical Therapy Association) as background reading and preparation for participation in onsite modules [2]. The emphasis of the onsite modules is development of clinical reasoning and advancement of skills in participants, with a focus on manual therapy clinical practice and evidence-based practice. In addition to onsite modules and online resources, residents receive clinical mentoring by instruc- tors from the United States. | Lecture/Didactic Approach Mentorship Skill Practice Student Assesment |
| Cunningham 2021 | Residency and Fellowship | duration: 18 months  acrd: yes  aop: MSK | The residency program in Kenya was based on the current residency model for physical therapists in the United States. The 18-month program consists of 12 weeks of onsite didactic and practical education, online education, and clinical mentoring. The mission of the residency program is to graduate physical therapists who can guide their communities and profession in the advancement of quality patient care and education. | Lecture/Didactic Approach Mentorship Skill Practice |
| Cunningham 2022 | Residency and Fellowship | duration: 18 months  acrd: yes  aop: MSK | The 18-month residency program included self directed learning, onsite modules, and one-on-one mentoring with residents in the clinical setting. The program was organized into six distinct modules. To progress in the program, residents had to achieve adequate performance of 75% on a written and a practical examination provided at the completion of each module. Following completion of the 18-month residency program, residents were required to successfully pass a comprehensive written examination and a live patient practical examination.  The didactic portion of the program utilized the Clinical Practice Guidelines from the American Physical Therapy Association Orthopedic Section and Current Concepts of Orthopedic PT (3rd ed.) as background reading and preparation for participation in onsite modules. Each of the six onsite modules consisted of 10 days of onsite education provided by physical therapy instructors from the United States. Instructor qualifications included being a faculty member within an accredited United States physical therapy program or having an advanced certification in both orthopedics and manual therapy.  In addition to onsite modules and didactic resources, residents received clinical mentoring by instructors from the United States. The mentorship focused on integrating the knowledge and skills learned during the residency program into clinical practice and emphasized advanced clinical reasoning skills. Mentoring supported the mission of the program to graduate advanced orthopedic physical therapy practitioners.  Mentoring occurred in timeframes separate from the onsite modules, allowing for additional contact with instructors and opportunities for the clarification of the application of residency materials. | Lecture/Didactic Approach Case Study Approach Mentorship Self Reflection Skill Practice Student Assessment |
| Demmelmaier 2012 | Multiple Encounter Course | duration: 15 hours over 20 weeks acrd: no aop: MSK | The intervention was based on social cognitive theory and emphasized individual goal setting, skills training, and feedback throughout the intervention. The intervention comprised two phases: 1) knowledge acquisition and 2) skills training. It was framed by a short introduction (session 1) and five 3-hour group sessions during 20 weeks (sessions 2–6). The structure and content of the intervention is presented in Table 2. The sessions took place in a conference room at the clinic and were led by the first author with assistance from the last author.  At the first session, the participants received relevant sections of The New Zealand acute low back pain guide (Kendall, Linton, and Main, 1997), including a Swedish summary. They also received a scientific article describing prognostic factors in MSK disorders (Gatchel and Turk, 2004). They were given four written questions to guide their reading, focusing on knowledge, perceived importance, and perceived barriers in assessment of yellow flags. Two weeks after receiving the written material, the second group session was held. It included structured discussions based on the four written questions and the participants’ own summaries of steps taken in knowledge and attitudes. Discussion about guidelines. Feedback on performance in recorded consultations. Development of check list. Feedback on performance by listening to recordings. Individual goal setting, repeated follow up and adjustment. Follow-up and adjustment of individual goal setting. Feedback by listening to recordings. Individual feedback on performance by e-mail/telephone. Individual plan for relapse prevention Reflection on guidelines and perceived obstacles. Assessment of prognostic factors in authentic consultations, according to individually set goals Individual feedback on performance by e mail/telephone. Continuous use of assessment skills according to individual relapse prevention plan | Lecture/Didactic Approach Case Study Approach Skill Practice |
| Dennis 1987 | Accredited Area of Practice Training | duration: not reported acrd: yes aop: MSK | Graduate Diploma programmes has been the hallmark of recent professional development in Australia.  Unlike Masters and Doctorate programmes, these were designed to offer better understanding of assessment and patient management skills to practising therapists.  and sixteen manipulative therapists (eight females and eight males) were selected from the Australian Physiotherapy Association register of private practition-ers, Melbourne branch (1982) and the register of Manipulative Therapists.  Graduate Diploma programme in manipulative therapy may show a focussed approach because they are using the decision rules of that discipline, or they may show an eclectic approach integrating their new skills with previously learned techniques. | Skill practice |
| Deutscher 2014 | Single Encounter Course | duration: 28 hrs  acrd: yes aop: MSK | The McKenzie postgraduate educational program consists of four 28-hour courses (Parts A through D) and a qualification credential examination. Parts Aand B educational courses consist of (1) lecture format augmented by demonstration of examination and treatment by the instructor on several different volunteers and real-time patients experiencing lumbar (Part A) and cervical (Part B) pain, and (2) open discussions throughout the course to enhance the participants’ understanding of the practical application of the McKenzie approach. Parts C and D are considered advanced training, with a major emphasis on problem solving and case studies, clinical reasoning for patient classification, and practicing manual spinal mobilization techniques. Only after all training levels are completed, a 1-day qualification credential examination, consisting of written and practical testing modules, is offered to demonstrate a basic level of competency in applying the McKenzie method. This study investigated the impact of any level of Mckenzie training* | Lecture/Didactic Approach Case Study Approach Skill Practice Student Assessment |
| Dizon 2014 | Single Encounter Course | duration: 1 day acrd: no aop: non-specific | Development of the intervention - Preliminary studies were undertaken prior to this RCT to inform the development of the program and to identify specific strategies to enhance the delivery of the program to the participants. The EBP training program was entitled the ‘EBP for FilPTs’. The training program was modelled as a complex intervention with “fixed/constant’ and ‘variable’ components. It was also underpinned by theories of adult learning, educational strategiesand the evidence regarding the effectiveness of EBP training programs. It was then designed in the context of the needs of the physical therapists and the nature of the local practice considering that they are not first contact practitioners. ‘Fixed’ components: The ‘fixed’ component of the EBP for FilPTs is the one day face-to-face training, in the form of lectures and practical sessions, consisting of the following lectures with supporting practical sessions (to influence skills in EBP): 1. Introduction to EBP 2. Hierarchy of evidence and study designs 3. Drafting the clinical question using the PICO format 4. Designing the search 5. Critical appraisal of the evidence and 6. Answering the clinical question based from the evidence found. ‘Variable’ components: The ‘variable components of the program are the EBP Checklist, the online EBP support, use of printed materials and more time for practical sessions. The EBP Checklist (Additional file 1) is to make evidence-based recommendations to referring doctors. It contains items related to validity and applicability of the evidence, magnitude of effects and capacity of the physical therapist to deliver the evidence-based patient management. Physical therapist would also have access to the training materials (lectures and references) in case they need to refresh their knowledge regarding the EBP. | Lecture/Didactic Approach Skill Practice |
| Fary 2015 | Multiple Encounter Course | duration: 4 modules over 4 weeks acrd: No aop: MSK (RA) | 4 learning modules and 2 clinical case studies based on outcomes of our previous work. The modules include 1) RA: the disease and recognition in practice; 2) RA: the early stage of the disease; 3) RA: the chronic stage of the disease; and 4) extraarticular features of RA and comorbid conditions. Content within each module was focused on delivering essential knowledge and translating that knowledge into practical clinical skills, i.e., the “know” and “do” for best-practice physiotherapy management of RA. Essential related content was identified in our previous study that sought to define these criteria by performing an international Delphi study and critical appraisal of 15 RA clinical guidelinesKey information across all modules was then integrated into 2 practice-relevant case scenarios. | Lecture/Didactic Approach; Case Study Approach |
| Furze 2019 | Residency and Fellowship | duration: 1 yr acrd: yes aop: MSK and Pediatric | These educational programs aim to facilitate clinical reasoning abilities, improve communication skills, and enhance the use of evidence and patient management approaches beyond entry level competencies in specific specialty areas   The structured educational experience must include both didactic and clinical components and emphasize ongoing mentorship over direct patient care.   Six physical therapy residents wrote reflective narratives across 4 time placements during their one-year residency | Lecture/Didactic Approach Mentorship Self Reflection Skill Practice Student Assessment |
| Green 2008 | Masters Level Program | duration: 1 yr acrd: yes aop: MSK | In 1992, at Coventry University, the MSc Manipulative Therapy programme was developed. It was the first of its kind in the UK and was developed in partnership with the clinical interest group, the Manipulative Association of Chartered Physiotherapists (MACP).  The MACP accredited the Coventry University programme, and students become eligible for MACP membership once they successfully complete the postgraduate diploma stage of the course.  n the UK, the MACP is the specialist manipulative therapy group recognised by the International Federation of Orthopaedic Manual Therapists (IFOMT). The IFOMT (2005) vision is ‘promotion of excellence and unity in clinical and academic standards for manual/musculoskeletal physiotherapists’. The MACP has a history of providing formal education and continuing professional development for manual therapists, and recognised the need to offer Master’s level study in a taught MSc  The Quality Assurance Agency (QAA) for Higher Education (2001) states that post-graduate students studying at Master’s level ‘will have shown originality in the application of knowledge, and they will understand how the boundaries of knowledge are advanced by research. They will be able to deal with complex issues both systematically and creatively, and they will show originality in tackling and solving problems’. For AHPs Master’s level education offers opportunities to explore practice and develop knowledge and clinical reasoning skills (Alsop and Lloyd, 2002) and according to the QAA (2001) Master’s level students will have the ‘qualities needed for employment in circumstances requiring sound judgement, personal responsibility and initiative, in complex and unpredictable professional environments The graduates from the Coventry University MSc Manipulative Therapy programme should, therefore, be ideally placed to fulfil the new roles within the NHS as they have attained a recognised Master’s level qualifica- tion from a programme that aims to ‘enable the development of senior practitioners into specialist roles within clinical practice, research and educational envir- onments’ | Lecture/Didactic Approach Mentorship Skill Practice Student Assessment |
| Hansell 2023 | Single Encounter Course | duration: 10.2 hrs ±5.3 acrd: yes field: Cardiorespiratory | Purpose: This unit is designed to cover the theoretical and practical curriculum for lung and diaphragm for acute care physiotherapists.  Prerequisites: Learners should have completed the ASUM Physics Image Optimisation unit or accredited equivalent. Training: Recognised either through attendance at an ASUM accredited Lung and Diaphragm course or equivalent.  Assessments: Learners are required to perform supervised ultrasound scans with documentation in a logbook. Unit Objectives  On completing this unit the candidates will be able to: Understand the role of lung and diaphragm ultrasound in acute care, Have an awareness of evidence, use and limitations with lung and diaphragm ultrasound, Identify and demonstrate practical understanding of: Basic settings of ultrasound scanning equipment , High and low frequency probes, uses, limitations, Patient and ultrasound probe positioning for lung and diaphragm ultrasound , Attain basic skills in ultrasound imaging of normal thoracic anatomy (ribs, intercostal space, pleura, diaphragm, heart) and key abdominal organs (liver, spleen, kidneys) , Attain understanding of key ultrasound findings of: lung, pleural pathologies and diaphragm dysfunction The unit will cover image interpretation and the clinical context of lung and diaphragm ultrasound, including: Approaches to scan the lungs/chest wall, and the limitations and the important principle that enough of the lung surface must be scanned to be able to rule in/out the disease that is in question. Ultrasound artefacts and equipment settings, probe orientation to optimize visualisation of the relevant thorax artefacts including the role that certain controls (such as gain and depth) have in regards to normal and key lung/pleural diseases. Teaching methodologies: A pre-test shall be conducted at the commencement of the unit, which focuses on the main  learning points.; An appropriately qualified clinician will be involved in both the development and the teaching of the course and will be present for at least part of the course itself.; Each course shall comprise at least 4 hours of teaching time, of which at least 1 hour shall be practical teaching, and another hour interpreting images of normal and pathological lung US findings and/or ultrasound phantoms. Stated times do not include the physics, artefacts and  basic image optimization which should be provided if delegates are new to ultrasound. : The lectures presented should cover substantially the same content as the notes in this syllabus.; The attendees will receive handout materials of all the presentations and practical checklists for the practical sessions. ; The live scanning sessions for this unit will include normal patients and either simulators, mannequins or patients to demonstrate pleural effusions and other pathology. Other lung pathologies not covered will be demonstrated by ‘image interpretation’ sessions in which the candidates must interpret the images/videos of the relevant pathology. | Lecture/Didactic Approach Skill Practice Student Assessment |
| Harrison 2022 | Multiple Encounter Course | duration: 8 hrs, over 3 months  accd: no aop: non-specific | The training program was delivered over 3 months. Training addressed all five steps in the evidence-based practice process (Albarqouni, Hoffmann, and Glasziou, 2018) and 64 of the 68 core competencies for evidence-based practice training (Albarqouni et al., 2018). The focus of the training was on therapy, so competencies which relate to appraising diagnostic, prognostic, etiology and qualitative studies, respectively were not covered. The face-to-face content was delivered at each hospital in existing protected time for professional development. All physical therapists at each site whether involved in the research project or not received the education and training. Training was tailored to the knowledge and skill mix of the participating physical therapists and addressed the barriers to evidence-based practice pertinent to that site (based on pre-training data). A flipped classroom approach was used. The program delivered approximately 8 hours of content (through three 90-minute face-to-face practical sessions and 10 video lectures) and consolidation tasks after each practical session (see Appendix Table C2). Practical sessions involved group work, case studies, interactive activities and role play. These were facilitated by academics and clinicians with expertise in evidence-based practice. The 10 videos covered the theory of evidence-based practice and were delivered by experts in each step of the process. A description of the training program using the Template for Intervention. Description and Replication (TIDieR) checklist (Hoffmann et al., 2014) is in Appendix Table C1. While we did not explore the equivalence in quality of training across the sites, systems were in place to promote similar training. The number of people attending each practical session and the number of times each video was viewed were recorded. | Lecture/Didactic Approach Case Study Approach Skill Practice |
| Heneghan 2022 | Mentorship | duration: 150 hours acrd: Yes aop: MSK | Students individual learning contract learning outcomes aligned to IFOMPT educational standards, assessment and management of a range of patients, full infirmed consent gained by mentor/mentee, following mentee patient history taking, mentees reflect on data to plan for physical examination, physical examination and management provided as required, mentor observation of mentee, peer mentoring facilitated through observation of mentee, patient centred care supported with resources, ongoing formative feedback and discussion of clinical reasoning for all cases. | Case Study Approach Mentorship Self Reflection Skill Practice Student Assessment |
| Jones 2008 | Residency and Fellowship | duration: not reported acrd: yes aop: MSK | APTA describes a physical therapist clinical residency as “a planned program of post- professional clinical and didactic education that is designed to advance significantly the physical therapist’s preparation as a provider of patient care services in a defined area of clinical practice | Lecture/Didactic Approach Mentorship Skill Practice |
| Kafri 2023 | Single Encounter Course | duration: 20 hours acrd: no aop: non-specific | The didactic module included an interactive, 20-hour course composed of an introductory presentation of the general concepts in Motor Learning, practice variables, and learning strategies. The content was delivered using multiple educational strategies. A detailed description of the module is presented in Additional file 1. Participants received handouts of the presentations, a list of selected references and links to relevant educational resources. A PhD-level physical therapist and motor learning researcher delivered the module. | Lecture/Didactic Approach Case Study Approach Skill Practice |
| Karas 2016 | Single Encounter Course | duration: not reported acrd: no  aop: MSK | The initial Knowledge Translation education programme began with an in-service reviewing two types of thoracic spine manipulation: a seated distraction and a supine thrust as described by Cleland et al. (2007c) and Gibbons and colleagues (Gibbons, 2000; Gibbons and Tehan, 2002), respectively. We chose these two techniques because they, or variations of them, are commonly used in MT. Other references may have been used, but our main purpose was to provide a description to standardize the education and treatment process in the best way possible. The review consisted of a brief introduction to the techniques, the supporting evidence, demonstrations of the techniques, hands- on assessment of the PTs’ execution of the techniques, time to practice, and follow up questions. Following the programme, the PTs were given descriptions of the techniques, accompanied by pictures. All of the PTs had utilized these techniques in the past; however, their exposure varied from prior continuing education, PT school or being taught the technique from a colleague. We utilized a multimodal education approach, including written and hands-on education, and online evidence summaries, to attempt to increase the use of thoracic spine manipulation by PTs for patients with neck pain. | Lecture/Didactic Approach Skill Practice |
| Karvonen 2015 | Multiple Encounter Course | duration: 5 days  acrd: no aop: MSK | 5-day intensive course entitled ‘Physiotherapy for LBP in the early phase; physiotherapeutic examination and instruction’ 2 contact days: DDx in MSK medicine, s/s, psyco-social factors, red flags, guidelines for meds 3 contact days: CR in PT, clinical guidelines for LBP, clinical tests (theory and praxis), sub-classification (theory and practice), therapeutic exercise 12 hrs: independent training of clinical tests in peer mentor groups at workplace in btw contact days. | Lecture/Didactic Approach; Mentorship Skill Practice |
| Kerssens 1999 | Multiple Encounter Course | duration: 28 hrs over 6 months acrd: no aop: non-specific | Communication skill training consisted of learning to clarify patients’ perceptions, motives, and resistance. All the physiotherapists brought in a case study from their own practice. These cases were mostly patients with somatic complaints and considerable psycho-social problems. Adherence enhancing skills consisted of the application of various strategies. The training was based on a manual which describes 11 strategies to enhance adherence. They were trained to improve their communi- cation skills and the transfer of adherence enhancing skills. Patient education, as they practiced it in the pre-training stage, was assessed by means of registration of each session of five to 10 patients by the physiotherapist and by patient questionnaire, administered at the start of therapy (T1), at the end of therapy (T2) and 6 months later (T3). After the training the same procedure was followed. | Lecture/Didactic Approach Case Study Approach |
| Lambrinos 2023 | Single Encounter Course | duration: 6 hrs acrd: no aop: Cardiorespiratory | The education reviewed theoretical and practical components of MI-E prescription and application. This was in the format of mixed media, including summaries of evidence, videos created by the expert physiotherapists (EL, AP, OM), demonstrating practical components, links to relevant resources, self-directed readings, case studies, and quizzes. Completion of the education course was targeted to improving knowledge on initial MI-E set up, titrating settings, assessing therapy efficacy, and problem solving | Lecture/Didactic Approach Case Study Approach Student Assessment |
| Lane 2022 | Single Encounter Course | duration: 16 hours acrd: no aop: pain | 16-hour PNE training program. Eight hours of training was provided online, then supplemented with a 1-day, in-person, practice session, designed to improve carryover into clinical practice.  The online content will include the majority of the didactic portion of the training. The 6-h in-person session is designed to accomplish the following objectives: 1) review the most important concepts from online training; 2) demonstrate incorrect vs effective PNE approaches using actual case examples, 3) practice proper assessment and PNE implementation using role-playing, and 4) Discuss how to implement a PNE approach in conjunction with the therapists’ current therapeutic approaches of manual therapy and exercise. | Lecture/Didactic Approach Skill Practice |
| Lawford 2018 | Multiple Encounter Course | duration: 3-months (3 training days total) acrd: no aop: MSK (OA) | Training program. Physical therapists completed a training program delivered by HealthChange Australia, which provides a health service delivery methodology (HealthChange Methodology) for person-centered care, including promotion of health literacy, shared decision-making, behavior change, and self-management to support adherence to evidence-based recommendations. HealthChange Methodology operationalizes and integrates psychological theories and principles commonly used as the basis for complex behavioral interventions (36–38), drawing on similar techniques to those used in motivational interviewing, solution-focused counseling, and cognitive behavioral therapy. The program combines 3 main processes to facilitate health behavior change: formulating a behavioral goal intention, converting intention into action and maintenance, and person-centered communication processes (39). Specific person-centered practice principles and behavior change techniques involved in HealthChange Methodology are described in Table 1. The methodology also restructures consultation tasks (see Supplementary Figure 1, available on the Arthritis Care & Research web site to align with patient information and decision-making needs, guiding clinicians to avoid or recognize and address potential patient adherence barriers. The training program comprised 2 initial training days (HealthChange core training part 1), skills practice and audit over 3 months, followed by 1 final training day (HealthChange core training part 2). All training days were run by a senior facilitator (CB) in a workshop-style format, involving lectures, role-playing exercises, group discussions, and video demonstrations. During the initial training day, physical therapists were provided with a lecture note booklet and a HealthChange miniguide, containing a series of cue cards relating to principles and processes taught. The first 2 training days covered 3 key components of HealthChange Methodology: practice principles to guide effective communication and knowledge transfer, techniques to identify and address barriers to behavior change, and a 10-step decision framework to guide consultation decision-making. Following the first 2 training days, each physical therapist was assigned 4 patients with knee OA to practice the processes taught during training over a 3-month period. Patients were provided with a folder that included information to increase knowledge about the importance of motivation for success with self-management, a personal self-management plan, and exercise instructions. Physical therapists were provided with a structured consultation framework using HealthChange Methodology, embedded within online treatment notes. Each physical therapist conducted 2 consultations over the telephone (2 weeks apart) with each patient. These calls aimed to commence the patient on a structured strengthening and/or physical activity program, and then review progress and modify the program/plan as needed. All calls were audio-recorded and reviewed by the training facilitator, and physical therapists self-audited their own calls against a customized check-list. The final training day reviewed key concepts and discussed audit findings. Physical therapists were financially compensated for the time invested in the training and self-audit activities, as well as for participating in the semi-structured interviews. | Lecture/Didactic Approach Case Study Approach Skill Practice |
| Lawford 2019 | Single Encounter Course | duration: 2 days (16 hrs) acrd: no aop: non-specific | Physiotherapists completed a training programme delivered by HealthChange Australia which provides a health service delivery methodology (HealthChange® Methodology) which enables health service providers to embed person‐centred care into clinical consultations, care planning, discharge planning, disease management, health promotion, rehabilitation, return to work and other health services. HealthChange® Methodology provides a framework which aims to help health service providers improve client health literacy, shared decision‐making, self‐management and behaviour change. The Methodology integrates numerous models and theoretical concepts of behaviour change, addressing three main processes to facilitate optimally health behaviour change in clients: (a) formulating a behavioural goal intention; (b) converting intention into action and self‐regulation; and (c) person‐centred communication processes (Gale & Skouteris, 2013). HealthChange® Methodology defines a set of person‐centred practice principles and techniques that allow and encourage self‐reflection, as well as enabling assessment of how well the intervention is implemented. The training programme comprised two back‐to‐back training days, run by a senior facilitator (C.B., a musculoskeletal physiotherapist) from HealthChange Australia in a workshop‐style format. This involved lectures, practice activities, group discussions and video demonstrations of how to apply person‐centred practice principles and techniques (Table 1) in clinical scenarios. The workshop provided training in how to apply HealthChange® Methodology's nine person‐centred practice principles, seven essential behaviour change techniques (Table 1) and the 10 Step Decision Framework (Figure 1), in clinical practice. Physiotherapists were provided with lecture notes, and a mini‐guide summarizing HealthChange® Methodology principles and techniques. At the end of the workshop, physiotherapists were provided with a structured consultation framework to use during their initial and follow‐up telephone consultations for the RCT, embedded within online treatment notes, with prompts for when, and how, to use HealthChange® Methodology in their consultations | Lecture/Didactic Approach Self Reflection Skill Practice |
| Levsen 2001 | Multiple Encounter Course | duration: 1 year  acrd: no aop: MSK | A Long-Term Course in Manual Therapy: Maitland Approach, presented by an expert physical therapist with significant post-graduate, education in manual therapy. The course involved. class participation one weekend a month for 12 months, with approximately 16 hours of class time on those weekends. Content focused on musculoskeletal examination and intervention. In addition, participants were required to develop case study presentations emphasizing clinical reasoning between each months session.Certifications of completion were issued based on attendance. No competencies were required prior to or folding the course and there was no final exam. | Lecture/Didactic Approach Case Study Approach Skill Practice |
| Lonsdale 2017 | Single Encounter Course | duration: 8 hrs acrd: no aop MSK | physiotherapists participated in a 1-hour refresher workshop on evidence-based physiotherapy care for chronic low back pain. In addition, physiotherapists in the experimental arm completed 8 hours of communication skills training, details of which have been published previously. | Lecture/Didactic Approach Case Study Approach Skill Practice |
| Louw 2022 | Single Encounter Course | duration: 3 hrs acrd: no aop: pain | Therapists working for the national PT group had been given access to online, self-directed CE content, including PNE. The content of PNE is well-documented and consistent with other studies. A 3-hour presentation focusing on peripheral sensitization, central sensitiza- tion, biopsychosocial factors associated with pain, threat appraisal of the brain, nociception, stress and endocrine responses to pain, and various therapeutic endogenous strategies to ease pain were used. Images, metaphors and examples were used along with the educational content. | Lecture/Didactic Approach |
| Maas 2012 | Multiple Encounter Course | duration: 4 sessions over 6 months acrd: no aop: upper extremity | The program for both groups consisted of four 3-hour sessions. In sessions 1, 2, and 4, the participants worked on written cases that fully covered the patient profiles described in the guidelines. Session 3 consisted of a review of patient records using a set of quality indica- tors derived from the KNGF guidelines on record keeping. The main difference between the 2 interventions is that in the PA approach the tasks are structured, with a focus on performance rather than discussion, and roles are predefined. Each participant performed 3 roles: physical therapist, assessor, and simulated patient. Because the therapists were complete novices in the PA method, the process was supervised by a group coach PA: In the role of physical therapist, they analyzed the case by reasoning aloud and demonstrated (hands-on) diagnostic and treatment skills. Peer performance was assessed by using a global scoring sheet designed to support peer assessors in giving constructive feedback. It contained 3 performance categories (planning, performance, and evaluation) that were scored on a 5-point Likert scale (from 1much improvement needed to 5no improvement needed). Accordingly, qualitative oral improvement feed- back was given. CD: written cases were included in the program guide to allow for proper preparation, along with instructions and written ques- tions to guide the discussion pro- cess. After completion of the program in July 2012, and before the posttest, all participants received an e-mail with model answers to all of the cases that were discussed during the program to control for unin- tended differences in knowledge development between and within groups due to the influence of the group coach. | Case Study Approach Self Reflection Skill Practice |
| MacPherson 2019 | Residency and Fellowship | duration: not reported acrd: yes aop: MSK | Fellowships are considered the pinnacle of post-professional clinical training in the physical therapy profession as one is required to attain either board certification or residency training prior to admission to a fellowship [1]. The core goal of the extensive training is to enhance the development of advanced clinical reasoning and communication skills along with higher level application of evidence-based practice. This in-depth educational journey is believed to enhance personal practice standards as well as overall standards of the profession [2,3]. In the area of orthopedic manual physical therapy (OMPT), experiential, didactic, and reflective learning strategies are organized in a formal curriculum with a variety of structures.  In the United States, fellowship training is delivered using two primary methodologies, the on-site, or in- residence, fellowship and a hybrid learning approach. The former is a traditional approach in which the FiT takes residence as a clinician within the fellowship organization. In contrast, the hybrid, or blended learning, design allows the FiT to maintain employment with their existing organization, with training provided through a blend of in-person and digital-based delivery methods. | Lecture/Didactic Approach Mentorship Self Reflection  Skill Practice  Student Assessment |
| Madi 2018 | Masters Level Program | duration: 1 yr acrd: yes aop: MSK | postgraduate master’s level (M-level) programmes approved by the Musculoskeletal Association of Chartered Physiotherapists (MACP)  In terms of directed learning hours the minimum requirements for MACP approved programmes is 200 hours of theoretical learning, 150 hours of practical learning and 150 hours of mentored clinical practice. The programme examined in this study has a modular structure (Box 5.1) that allows full-time students to complete their postgraduate diploma (i.e. M-level qualification) in less than a year, and thus become eligible for MACP membership  Research Methods  Advancing Practice through Personal and Professional Development (APPPD) Advanced Musculoskeletal Physiotherapy 1: Lower Quadrant (AMP1)  Advanced Musculoskeletal Physiotherapy 2: Upper Quadrant (AMP2)  Evidence Based Clinical Practice (EBCP)  Optional module  Physiotherapy educators are reported to utilise a range of educational approaches to advance clinical reasoning skills, such as problem-based learning, guided observation, capturing and reflecting on therapists’ reasoning style (Ryan and Higgs, 2008), using actual or simulated patients (Edwards and Rose, 2008), case reports (Rivett and Jones, 2008) and professional socialisation.  Raising awareness; Promoting critical thinking and challenging beliefs; Process of knowledge deconstruction and evoked students’ critical thinking and reconsideration of their model of practice (i.e. frame of reference). Promoting Examining prior experiences in the context of M- reflection and level knowledge and skills. introspection: An ongoing process of raising awareness through feedback comprehensive, regulatory and relational feedback. Collegial knowledge exchange: Learning from Promoting peer learning and sharing experiences peers and skills with colleagues and a process of passing on programme experience from one cohort to another. Collective knowledge construction: Making sense of the new knowledge and skills in pairs, small groups, and whole class discussion and debates.: Continued Continuous process of learning engagement learning through self-interaction in self-directed studies: informal university discussions, and moving the conversation to workplace settings.: Dynamic learning environment: Recognising cohort characteristics: Adapting the learning environment to meet the cohort’s biography, background knowledge and size: Tailored and flexible learning environment: A personalised learning culture that meets students at their level and negotiates their learning needs.: Context of clinical reasoning advancement: Cohesive learning environment: The cohesion across and within programme modules supports learning transition through graded exposure.: Effective facilitators of learning: Educator’s preparation, attitudes and behaviours that support synergistic interaction with adult learners at an advanced level of criticality.  1) promoting clinical reasoning within the biopsychosocial framework; 2) promoting thinking within a patient-centred care framework; and 3) moving students away from technical rationality to considering professional artistic practices such as creative practice and embracing uncertainties.  Pedagogy included: students’ self-assessments such as SWOT17 analysis; educator-led interactive discussions, writing reflective journals, summative assessment in the forms of a viva voce examination and seminar presentation Students are exposed to a range of educational strategies ... obviously lectures that look at advancing knowledge ... but also the critical discussion and social interaction ... we anticipate that students will learn probably 40-50% through their engagement with their peer group or with the other students. (Educator) A collaborative and collective process of making sense of the new knowledge and skills in pairs, small groups, whole class discussions and debates. Continuous process of learning engagement through self- directed studies, informal university discussions and workplace learning. The programme structure enabled students to share knowledge beyond the point of educator-student contact. Some of this learning was purposefully planned through self-directed studies. | Lecture/Didactic Approach Mentorship Self Reflection Skill Practice Student Assessment Research assignments |
| Mansell 2020 | Multiple Encounter Course | duration: 1 year  acrd: no aop: Cardiorespiratory | Three training sessions provided four monthly interspersing SBE sessions. – 1 hour long: MDA competencies; – Paediatric update; Practical skills refresher. In addition, two SBE training sessions lasting 3 1⁄2 hours each spaced 6 months apart. Four to five candidates in a group for SBE. Mixed experience and specialty groups. | Lecture/Didactic Approach Case Study Approach Skill Practice |
| March 2024 | Single Encounter Course | duration: 3 hrs acrd: no aop: MSK | •Face-to-face educational workshop (video conferencing if required by COVID-19 restrictions). •Workshop to include didactic/instructional components and simulation components. •Structured approach to developing therapeutic alliance and communication. •Enhanced patient-led approach to goal setting.  3 hour face-to-face workshop, including interactive seminar components, simulation and debriefing. Two aspects of simulation were used during the face-to-face workshop. The primary mode was engagement with a simulated patient experiencing knee osteoarthritis. The simulated patient was used in a tag-in-and-out fishbowl simulation with all participants, a role play simulation in groups of two to three participants, and for debriefing in character after both activities | Lecture/Didactic Approach Case Study Approach Skill Practice Simulation Based Training |
| Murray 2015 | Single Encounter Course | duration: 8 hrs acrd: no aop: MSK | Physiotherapists from the intervention arm also participated in 8 hours of communication skills training, comprising two 4-h sessions. The first training session incorporated an overview of the main SDT concepts and covered strategies for implementing the communication skills during physiotherapy practice. Video recordings of simulated initial treatment sessions were shown. These vignettes first depicted a physiotherapist displaying controlling communication styles, which were then contrasted with depictions of needs-supportive communication behaviors. Active role-play and group discussion were also used. At the end of the session, each physiotherapist recorded 2 or 3 goals for strategy implementation during his or her treatment sessions in the upcoming week, along with likely obstacles and anticipated solutions. Physiotherapists were provided with choices regarding these goals; they were advised to choose strate- gies that they believed required most improvement or would have the most benefit for their patients. The second training block consisted of group discussion regarding the facilitators and barriers to implementing the communication strategies during the previous week. Further simulated video recordings of follow-up physiotherapy sessions with a controlling versus needs-supportive communication style were shown, followed by group discussion between the physiotherapists and the workshop leader. At the end of the session, physiotherapists revised and set new goals regarding their implementation of the SDT-based strategies For example, one physiotherapist set a goal to help her patients with CLBP set simple, measurable, achievable, recorded, and time-based (SMART) goals regarding their home-based rehabili- tation exercises, and another set a goal to replace a common controlling phrase (“I want you to do this for me, ok?”) with a more needs-supportive suggestion (“If you do this, you’ll give yourself the best chance for improvement”). As in the first session, physiotherapists were advised to choose goals related to strategies they believed required most improvement or would have the most benefit for their patients. | Lecture/Didactic Approach Case Study Approach Skill Practice |
| Naidoo 2022 | Residency and Fellowship | duration: 1 year acrd: yes aop: MSK | In addition to patient care and educational hours, residents participated in community service, mentored scholarship, and served as laboratory instructors in a Doctor of Physical Therapy (DPT) program.  Residents passed two LPEs and three written examinations, and met other graduation requirements to successfully complete the residency. | Lecture/Didactic Approach Case Study Approach Mentorship Self Reflection Skill Practice Student Assessment |
| Ntoumenopoulos 2017 | Single Encounter Course | duration: 1 day acrd: no aop: Cardiorespiratory | a one-day DTU course that included 2.5 h of didactic lectures and 4 h of expert-led practical skill training (Table 1). Half of the course participants were staff members of the institution conducting the DTU course and they were expected to also undertake a practical skill examination on the day after the course. Prior to the course the participants were sent a key narrative review article,for its comprehensiveness to enhance learning and reduce the cognitive load during the workshop, that covered the basics of DTU theory and its practical applications in critical care including the identification of the key pulmonary pathologies such as pneumothorax, pleural effusion, lung collapse, lung consolidation, pneumonia and interstitial oedema. The experts who led the practical training sessions included three medical doctors (HCT, RPS, WDS), one experienced trained ultrasonographer and an experienced trained critical care physiotherapist (GN) all with current clinical and teaching experience in diagnostic thoracic ultrasound. | Lecture/Didactic Approach Skill Practice |
| Olsen 2015 | Multiple Encounter Course | duration: 6 months acrd: no aop: non-specific | The intervention consisted of a multifaceted and clinically integrated training program in EBP (6 ECTS-credits), delivered to CIs over a six-month period (October 2008- April 2009) (Table 1). The training program was multifaceted as combinations of several teaching strategies were employed: workshops, assignments, supervision and exams. The workshops were a mixture of lectures (didactic sessions) and small-group activities that required participants to be interactive. Four half-day workshop sessions covering the EBP steps and processes were held in classrooms at hospital settings (HUS) and delivered sequentially over a six-week period (Table 1). Between and after workshops, five individual written assignments (Week 2, 4, 7, 1, 21) were required from the participants. To ensure clinically integrated learning of EBP, each assignment required participants to reflect on and describe how to apply the EBP steps in real clinical situations. Assignment one and two required participants to write a coherent paper on identified clinical information need and question formulation. For the three first assignments they had to reflect on how to supervise students in using the EBP steps. Assignment three to five required participants to work through all EBP steps using the EBP tool (Table 2). The EBP tool can be used to document the EBP steps and process: information need, clinical question asked, search strategy and result of this, critical appraisal of the research results found (validity, reliability and applicability), if and how the research results are integrated or shared in clinical practice and how the changes made to clinical practice are evaluated. The EBP tool is a learning tool intended to provide health care professionals with practical EBP skills. The EBP steps and processes registered in one document facilitate the learning process and the possibility of receiving and giving feedback. Development of this tool was inspired by working files developed for trainee doctors in Norway [28]. For each assignment, participants received supervision via phone and/or email, in addition to guidance from a librarian when necessary. All five assignments had to be completed before the final exam. The exam took form of an individual oral presentation, where participants focused on how to apply the EBP steps to a real patient situation and how to supervise students in the EBP process (Week 27) (Table 1). The exam was assessed as pass/fail. The objectives of the program (Table 1) were related to the EBP steps and processes described previously. The program was delivered by a project group of five physiotherapists (including NRO, HL, BF), from both academic and clinical positions, and with a range of expertise in EBP, physiotherapy, higher education and research. | Lecture/Didactic Approach Mentorship Self Reflection Skill Practice Student Assessment |
| Overmeer 2009 | Multiple Encounter Course | duration: 8 days over 8 weeks  acrd: yes aop: MSK | The course was aimed at identifying and addressing psychosocial prognostic factors within physical therapy treatment. Main sources used during its development were a book on biopsychosocial approach dealing with pain patients [18], a systematic review of psychological factors as predictors of chronicity or disability [19] and the New Zealand guidelines for low back pain [20]. The course focused on theory, role-playing to practice new skills and feedback on the practised skills comprised. Sixty-four actual teaching hours of 8 hours per day once a week on eight consecutive weeks permitted the practice of newly acquired skills between teaching sessions. Homework assignments with patients they were treating in their own clinical practice enabled feedback on the practiced skills the following week. | Lecture/Didactic Approach Case Study Approach Skill Practice |
| Overmeer 2011 | Multiple Encounter Course | duration: 8 days acrd: yes aop: MSK | a postgraduate course consisting of e-learning and two workshops (blended education) on the application of a strategy for exercise prescription in patients with KOA and comorbidity. The course was provided by the authors and was aimed at identifying and addressing psychosocial prognostic factors within physical therapy treatment. The course focused on theory, role playing to practice new skills, and feedback on practiced skills. The theoretical content of the course included lectures about “yellow flags,” patient-practitioner communication. Yellow flags” refer to psychological and social/environmental prognostic factors for disability and work loss in patients with recent onset of MSK pain (ie, belief that pain necessarily implies damage or distressed mood). Practical training of new skills by means of role playing followed each theoretical section. Between teaching sessions (64 actual teaching hours; 8 hours per day onceper week for 8 consecutive weeks), participants could practice newly acquired skills as homework assignments with patients whom they were treating in their own clinical practices; they could obtain feedback on practiced skills at the teaching session the following week. University credit was given and was equivalent to studying 25% during the term. | Lecture/Didactic Approach Case Study Approach Skill Practice |
| Perry 2011 | Masters Level Program | duration: 1 yr acrd: yes aop: MSK | Clinical Masters (MSc) programme in manipulative therapy (MT) at Coventry University; The MACP accredited the Coventry University programme.  Postgraduate study offers opportunities for students to explore, develop and question practice and knowledge through the processes of critical analysis, knowledge synthesis, clinical reasoning and problem solving.  Masters education facilitates an irreversible, integrative transformation of an individuals internal perspective with a concomitant extension of the students breadth of discourse and personal/professional identity.  This was a follow-on study from a postal questionnaire that explored the participants’ career pathways following Masters education in manual and manipulative therapy (Green et al., 2008)  Master’s level education offers opportunities to explore practice and develop knowledge and clinical reasoning skills | Lecture/Didactic Approach Mentorship Skill Practice Student Assessment |
| Peter 2013 | Single Encounter Course | duration: IW 3 hr; CE 2 hrs acrd: yes aop: MSK (OA) | Interactive Workshop (IW) The workshop started with a short summary of guideline recommendations. Subsequently the participants were divided in subgroups of 810 PTs. The patient presented his or her complaints and their consequences for daily activities and participation. More information was gathered by interviewing. Within each subgroup decisions were made concerning initial assessment, treatment modalities and the measurement instrument to be used, based on clinical reasoning. PTs and patients taking part in the educational course could provide feedback concerning all the decisions made. During this process the expert PT was available to give additional feedback. In a plenary session, the IW ended with a discussion about a fictional case and questions concerning the content of the guideline. The IW workshop lasted three hours.  Conventional Education (CE) It comprised a presentation about the guideline developmental process and the recommendations in the guideline. Two different cases were presented to the group (one patient with hip OA and one with knee OA) and their initial assessment, treatment and the evaluation of treatment by means of measurement instruments were described, all according to the guideline. The educational course lasted two hours. | Lecture/Didactic Approach Case Study Approach Skill Practice |
| Peter 2015 | Single Encounter Course | duration: 3 hours acrd: yes aop: MSK (OA) | The interactive, educational course was developed and evaluated in a previous pilot study [13]. The course was guided by an expert physiotherapist, in cooperation with three to four patients and three to four physiotherapy teachers, who were instructed concerning their role during the course (Appendix 1). A process of clinical reasoning was followed within the educational course according the Hypothesis-Oriented Algorithm for Clinicians (HOAC) II principles [17]. The course lasted 3 h and was offered free of charge. In both groups the participants received accreditation from the national professional organization for the educational course (four Continuing Education points), provided after they had attended the educational course and completed all questionnaires. | Case Study Approach Skill Practice |
| Petty 2011  (Knowledge Acquistion) | Masters Level Program | duration: 1 year acrd: yes aop: MSK | MSc Neuromusculoskeletal Physiotherapy from one UK university.  Course requirement of 200 h of neuromusculoskeletal physiotherapy theory, 150 h of practical skills development and 150 h of mentored clinical practice.  The most powerful learning process of the MSc was on clinical placement with an MACP approved clinical educator; this involved direct observation of their clinical practice with patients with subsequent questioning, discussion and critical feedback. In addition, participants observed the educator with patients who sometimes became a really good role model (P10) The main focus of the course was developing practitioners diagnostic reasoning for patients with physical impairments and this was borne out in the data. Assessment findings were now used to guide treatment choice and gave me the freedom to create treatment techniques to suit me and the patient (P6) | Lecture/Didactic Approach Mentorship Case Study Approach Self Reflection Skill Practice Student Assessment |
| Petty 2011 (Learning Transitions) | Masters Level Program | duration: 1 year acrd: yes aop: MSK | These courses follow the educational standards of the International Federation of Orthopaedic Manipulative Physical Therapists with a minimum of 200 hours of neuromusculoskeletal physiotherapy theory, 150 hours of practical skill development and 150 hours of mentored clinical practice. A major focus of these courses is to develop clinical expertise and enable successful practitioners to obtain advanced clinical practice and extended-scope practitioner roles.  ‘I found the placements really, really helpful and even though it was very, very difficult, it was probably the most positive thing to come out of the course.   Participants expected to enhance their knowledge and skills progressively through didactic teaching from a skilled and informed tutor. They expected to be given information and be told how to perform skills: One thing I was surprised with was that I thought the teaching would be more prescriptive, I thought when I was being taught, I would be told do like this, this and this and somebody would come in and tell me how to do it. But it wasn’t actually like that; it was quite student led with lots of discussions.’ (P7) ‘The practical hands-on skill were taught differently than I was used to and the way I expected. On the weekend clinical courses, there was a way to do something. On the MSc, techniques were taught more openly, less prescriptive, which was liberating really.’  The contradiction that triggered learning involved critical evaluation of their practice knowledge. This was particularly pertinent within musculoskeletal-specific modules that involved 6 weeks of classroom-based teaching and 6 weeks of clinical practice. Some aspects of practice knowledge were more challenging than others. Giving and receiving critical feedback on their handling skills posed relatively little challenge; they were familiar with this during in-service training and weekend courses. Critical classroom discussion that explored theoretical knowledge and reasoning was less familiar and posed a greater challenge. By far the greatest challenge and most powerful learning experience was an MACP mentor directly observing their clinical practice with patients, and providing critical evaluation and feedback of their performance. | Lecture/Didactic Approach Mentorship Case Study Approach Self Reflection Skill Practice Student Assessment |
| Prizinski 2021 | Residency and Fellowship | duration: 1 yr acrd: yes aop: MSK | PTs were enrolled in a twelve-month orthopedic residency program which required an interactive mentorship component.   The residency program is an organized and structured post professional curriculum that allows novice physical therapists to learn advanced skills to achieve greater patient outcomes.  Following the participants taking the online learning modules, there were two live coaching session. The first live session involved discussing the application of the online material into behavioral styles, driving forces in communication, and potential barriers. The second live session involved the residents separate from the mentors. The resident session involved how they perceived implementing the learned material over the past 6 weeks with their mentors into patient centered care.   This CoP or learning environment is made up of mentorship, clinical practice (learning from patients), and the resident applying reflective and clinical reasoning skills to develop in their decision-making and patient centered approach during their first year of practice. The themes from this study are presented in greater detail with samples from the interviews. | Mentorship Student Assessment |
| Rebbeck 2006 | Single Encounter Course | duration: 8 hrs acrd: no aop: MSK | Intervention for the implementation group consisted of dissemination of guidelines, initial education by opinion leaders, and follow-up education. Physiotherapists in the implementation group initially attended a one-day (8 hour) workshop. The workshop included interactive sessions outlining the content of the guidelines, practical sessions covering the treatments endorsed in the guidelines, particularly those that were relatively ‘new’ for physiotherapists (ie, ‘reassure patient’ and ‘advise to act as usual’), and the use of functional outcome measures. Local opinion leaders were used to deliver some of the program content. Physiotherapists were given a laminated copy of the algorithms outlining the process of care (MAA 2001), appointment cards, and marketing material to be used for general practitioners who usually refer to the practice. They received a follow-up educational outreach visit (2 hours) approximately six months later. At this session, problem solving regarding use of the guidelines in clinical practice was undertaken and an update of the evidence given. Intervention for the dissemination group consisted of dissemination of guidelines by mail, ie, physiotherapists in this group were given but not directed to use the guidelines. Both groups were given the same information regarding the trial and its outcome measures | Lecture/Didactic Approach Case Study Approach Skill Practice |
| Resnik 2004 | Accredited Area of Practice Training | duration: not reported acrd: yes aop: MSK | Certification is the process by which a physical therapist builds on a broad base for professional education and practice to develop a greater depth of knowledge and skils related toa particular area of practice" The specialist certification process is coordinated through APTA byhte American Board ofPhysical Therapy Specialties (ABPTS)*. ABPTS developed criteria and procedures for specialist certification and recertification and based the orthopedic clinical specialists (OCS) examination upon the competency matrix identified in the Description fo Advanced Clinical Practice". As of 2002, 1854 therapists have been certified as an OCS  Manual therapy residency programs have existed since the Kaiser Permanente program was established in 1979. The American Academy of Orthopaedic Manual Physical Therapy (AAOMPT) was established in 1992°, and ti supports development fo clinical residency programs in manual physical therapy. AAOMPT has established standards for competency fo physical therapists trained in residency programs. In 2002 six residency programs were approved by AAOMPT®. In 1998 the APTA enacted voluntary  Manual therapy certification programs differ from residency programs. The former are offered by avariety of institutions and organizations. Several manual therapy certification programs, including those ta Kaiser Permanente®and the Institute of Orthopedic Manual Therapy',are also APTA-approved clinical residencies'®. Certification programs vary in structure, content, intensity and approach to their curriculum. Some programs involve coursework and oral and written examinations but do not include supervised clinical training | Mentorship |
| Rodeghero 2015 | Residency and Fellowship | duration: not reported acrd: yes aop: MSK | systematic mentored training of a residency or fellowship program  standardized program with an emphasis placed on mentorship | Mentorship |
| De Rooij 2020 | Multiple Encounter Course | duration: 6 months acrd: no aop: MSK | The blended learning course was developed by an expert PT/researcher (MROO) in collaboration with an educational instructional designer and teachers from the Amsterdam University of Applied sciences. The expert PT has >15 years of experience in treating patients with knee OA, is an expert in the development of comorbidity-related adaptations and is experienced in teaching health professionals. Each workshop was led by the expert PT and performed with the help of five PT teachers who were familiar with the treatment of patients with knee OA and comorbidity and thedeveloped strategy. The teachers received oral and written instruc-tions on how to guide the participants in the process of clinical reasoning. The blended educational course consisted of five e-learning lectures and two interactive workshops. The content of the educational course was based on the previously developed and teste strategy (de Rooij et al., 2017). The content and the topics that were educated in the e-learning lectures and workshops are summarized in table 1. After following the e-learning lectures and the two workshops, PTs treated patients with knee OA and comorbidity, during a 6-month period. Target comorbidities were coronary disease, heart failure, type 2 diabetes, COPD or obesity. During the intake the PT checked whether the patient fulfilled the clinical criteria of the American College of Rheumatology for knee OA (Altman et al., 1986). The Cumulative Illness Rating Scale (CIRS) was used to measure comorbidity (Hudon Fortin, & Vanasse, 2005; Hudon, Fortin, & Soubhi, 2007). Patients had to score a severity score ≥ 2 for the comorbidity on the CIRS, indicating that the comorbidity has an impact on daily activities and the patient was receiving regular care for the comorbidity | Lecture/Didactic Approach Case Study Approach Skill Practice |
| Rushton 2010 | Masters Level Program | duration: 1 yr acrd: yes aop: MSK | One ‘case’ of a university course in manipulative physiotherapy was selected. Manipulative physiotherapy is a specialist area of physiotherapy. Postgraduate development informed by international and subsequently nationally applied standards of educational practice (International Federation of Orthopaedic Manipulative Physical Therapists [IFOMPT], 2008) Recent development in healthcare knowledge and technology demands greater clinical expertise by healthcare professionals in managing more complex problems, a need that is addressed partly by masters level education. The emphasis on developing clinical expertise is evident in the recent proliferation of masters level courses, commonly taken following an initial Honours degree. The emphasis within the existing frameworks is on knowledge at masters level. Findings from limited research in healthcare do however identify different areas of importance for clinical courses, for example, the application of skills to the clinical context, improved confidence and clinical reasoning, and advanced clinical skills Components of the Characteristics of the component examination process --> Student–patient interaction Examiner–examiner interaction 1 Examiner–student interaction 1 Examiner–examiner interaction 2 Examiner–student interaction 2 Examiners observing student actions in assessing and managing patients Discussion of observations and identification of areas to explore Discussion of identified areas with student: Discussion of student performance, decision re grade and feedback Feedback to students on performance | Lecture/Didactic Approach Mentorship Case Study Approach Self Reflection Skill Practice Student Assessment |
| Schreiber 2012 | Multiple Encounter Course | duration: 90 mins + 16 weeks  acrd: no aop: Pediatrics | Unique aspects of the CE program included the following: grouping attendees based on practice setting for case-based discussion segments; integrating attendee cases into large group discussion segments; providing detailed printed support materials; a 90-minute session devoted to KT; and a follow-up Wiki discussion board for interaction among attendees and course instructors related to application of course information. Requirement that each participant create an individualized KT plan using the checklist in Table 1. This was based on the notion that successful KT requires additional reflection by the user on the various factors that may affect the process. During the 16-week period following the conference, these individuals participated in an online discussion group (a Wiki) that included posting of questions, comments, and interaction with other group members and the course instructors related to course content and implementation of the KT plan. | Lecture/Didactic Approach Case Study Approach Self Reflection |
| Seif 2019 | Multiple Encounter Course | duration: not reported acrd: no aop: MSK | The Orthopaedic Manual Therapy Certification is a 5-course series developed by faculty at the Medical University of South Carolina (MUSC). The course content focused heavily on examination and treatment of the spine, specifically low back and neck regions, utilizing the current evidence-based practice guidelines and emphasized the use of standardized outcome measures. At each subsequent course, there was an opportunity for the participants to review, ask questions, and practice skills from prior courses with instructors available for feedback. Participants completed patient case assignments after each course that were designed to demonstrate the integration of the new course techniques and their use of outcome measures. In order to receive the certification, participants had to attend all courses, successfully complete all post-course assignments, pass an online written examination, and pass an in-person practical examination | Lecture/Didactic Approach Case Study Approach Skill Practice Student Assessment |
| Shalabi 2024 | Single Encounter Course | duration: not reported acrd: no aop: non specific | online continuing medical education (OCME) courses | Not Specified |
| SjodahlHammarlund 2013 | Masters Level Program | duration: 10 weeks, 200 hrs acrd: yes aop: non specific | This study took place in the context of a master’s program where each student planned their selection of courses to be included in their master’s degree. Depending on individual needs, the course selection and the order in which courses were taken also varied. Among the optional courses, two self-directed online courses were developed in Movement Science  The design of the Movement Science courses Each course was based on self-directed learning during 10 weeks, part-time (i.e. equal to 200 h). The learning outcomes were the starting point directing the learning and working processes (6). The course had a clear structure involving specific deadlines for presenting assignments, peer-reviews and teachers’ feedback.The structure was based on the student activities and no predefined lectures or reading material was scheduled. During the first week, the participants proposed research questions, selected assignments, planned their schedules, and the content of each assignment based on their learning needs and in relation to the learning outcomes. To ensure that the content was scientifically adequate, the individual planning was processed in two steps; after an initial peer review and revision of the research questions (step 1), a teacher also gave additional feedback to ensure that the project was adequate in topic, depth and width to meet the learning outcomes of the course (step 2). Depending on the comprehensiveness of each assignment, each student worked on three or four assignments during the course. Each participant also selected how to communicate their findings, e.g. podcast, poster, quiz, patient information. To facilitate the direction of their collaborative work, the assessment criteria were available from the start of the course to scaffold the discussions and peer-reviews.  Instructions for writing the self-reflections When an assignment was completed, the participants were instructed to write their self-reflections. The guidelines to the self-reflections suggested the par- ticipants to consider e.g. “What went well? Why? If you would do this assignment again, would you do it in another way? What do you need to develop? How do you want to proceed?” | Self Reflection Skill Practice Student Assessment Research assignments |
| Smith 1999 | Residency and Fellowship | duration: 1 yr acrd: yes field: MSK | the oldest manual therapy residency program in the United States, the Kaiser Permanente Physical Therapy Residency Program in Advanced Orthopedic Manual Therapy in Hayward, California, which was developed in 1979. This program was modeled after yearlong manual therapy residency programs in South Australia. It is one of approximately 13 orthopaedic residencies operating within the United States, all of them recognized by the American Academy of Orthopaedic Manual Physical Therapy.  Curriculum content in the residency program is directed in part toward preparing residents for varying levels of teaching and consultation after graduation. priority of teaching clinical reasoning  the residency program has strong emphasis on developing clinical expertise and less emphasis on professional writing extensive time spent on review of the literature during the program is in the context of being able to read and review critically and apply findings to current clinical practice | Mentorship Skill Practice Student Assessment |
| Souter 2019 | Residency and Fellowship | duration: variable acrd: yes aop: variable | For the purposes of this study, formal post- professional development was considered completion of an accredited residency training and/or fellowship training program through the ABPTRFE. As opposed to informal post-professional development opportunities, such as many continuing education courses, accredited residency and fellowship programs provide structured learning experiences that reflect measurable learning objectives for prescribed competency domains. Further, assessment of content mastery is also a consistent feature of formal post-professional development, which is not often found in informal post-professional opportunities | Mentorship Self Reflection Skill Practice Student Assessment |
| Stathopoulos 2003 | Masters Level Program | duration: 1 yr acrd: yes aop: MSK | practising physiotherapists who had completed physiotherapy taught master’s courses provided by a UK university took part in a focus group interview.  The provision of postgraduate academic courses for professional physiotherapists has been suggested to enhance specific areas of professional practice in terms of development of a greater knowledge base in the professional field; development of academic generic attributes, including critical judgement and analytical skills; and development of research skills.  undertaking a master’s programme, emphasising particularly the importance of the development of academic skills, such as critical thinking, research skills, and ability for self-directed lifelong learning  graduates from physiotherapy based master’s degrees at a specific university in the United Kingdom Academic courses designed to extend cognitive skills in individuals | Lecture/Didactic Approach Skill Practice Rearch assignments |
| Stevenson 2006 | Single Encounter Course | duration: 5 hrs acrd: no  aop: MSK | The 17 physiotherapists randomized to the intervention group received an educational programme utilizing evidence-based principles and administered by local opinion leaders. At baseline, all the study participants were asked to identify opinion leaders in various areas of physiotherapy, including back pain management, chronic pain management, knee pain management, research, literature searching and critical appraisal. The local opinion leader identified in pain management was approached and agreed to lead this evidence-based session. This was based on ‘Psychosocial Yellow Flags’ as identified by Kendall et al. (1997). It included: advice about the work situation; advice on returning to normal activities; advice on increasing activity levels; encouraging early return to work; encouraging patients to undertake activities themselves and challenging patients’ unhelpful attitudes and beliefs about pain. The 13 physiotherapists randomized to the attention control group received a standard in-service training package on clinical management of knee dysfunction and pathology. Both groups received 5 hours of training. This approach was chosen to control for the non-specific effects of training such as extra attention and peer support. Each group was unaware of the study hypothesis and the difference in method of delivery, that is, an ‘evidence-based educational session’ or ‘usual’ in-service training | Lecture/Didactic Approach |
| Stevenson 2020 | Residency and Fellowship | duration: 1 yr acrd: no aop: MSK | The content of the ‘Musculoskeletal Interface Service Clinical Trainee Development Programme’ was informed by the needs of the applicants, identified at interview and by those with expertise in working in and leading the service Key components were clinical mentorship, a planned and progressed increasing clinical workload, built in time for reflection and personal development, directly observed clinical practice, reflection on practice and identifying best practice and audit.  Each trainee would be required to collect evidence to support each element of the programme and present a portfolio of evidence by the end of the 12 months. Trainees competency would be assessed when they felt ready to undertake the competency, in line with autonomous practice. When achieved, they could be signed off by a senior clinical member of the team   The programme was built on the principle that the trainee would require mentorship during clinical consultations and whilst interpreting results and planning patient management. Therefore, a mentor was named for each clinical session. Clinics started with four new patients for a 4 h slot, increasing on a 3-monthly basis to reach a maximum of six new patients and one follow up at 10 months. monthly peer observation of a clinic consultation was undertaken, verbal and written feedback was provided. Each trainee was required to work through a ‘Best Practice Framework’ which contained key competencies.  To achieve this, trainees, depending on their own learning style, could utilize peer support, case presentations, formal tutorials, formal course attendance, experiential learning, mentor and peer support, reflective practice and feedback and internal and external visits to achieve the competencies. Injection therapy training was commenced from months 9 to 12. This competency could be achieved though attending a formal masters module or experiential learning through mentorship. Both routes were governed by clear competencies, which included principles of injection therapy, health and safety issues, life-long learning, pharmacology, managing emergency situations, professional and legal issues, common injections and the evidence to underpin practice.  Over the 12 months, activities were phased in to compliment clinical activity. Clinics, with reduced patient numbers to allow for reading, self-directed learning, experiential learning, started immediately as did peer/observed assessment. The programme was designed to encourage trainees to undertake any learning within the working day | Lecture/Didactic Approach; Case Study Approach Mentorship Self Reflection Skill Practice Student Assessment |
| Swinkels 2015 | Multiple Encounter Course | duration: 4 days over 4-5 months acrd: No aop: non-specific | The programme consisted of four interactive half-day training sessions spread over 4–5 months. Between sessions, participants were instructed to use the measurement instruments in the toolkit with patients in their clinical practice; coaching and feedback were provided during the four training sessions. In each session, participants discussed the instruments in the toolkit; their use in daily practice for diagnostic, prognostic, or evaluative purposes; and the interpretation of test results in relation to their own patients and in the process of clinical reasoning. In addition, physiotherapists were taught how to overcome organizational barriers in their own practice settings (e.g., by sending out questionnaires in advance or using special software). | Lecture/Didactic Approach Case Study Approach Skill Practice |
| Synnott 2016 | Multiple Encounter Course | duration: 9 x 12 hrs acrd: no aop: MSK | All participants had received CFT training from CFT trainers (www. pain-ed.com) (inclusive of authors KOS, POS and WD). Training included both workshop attendance, in which they observed CFT trainers assessing and treating live patients, and supervision of clinical practice. All participants had participated in at least two CFT workshops (average of nine workshops completed to date, average duration of 12 hours), and were supervised by CFT trainers for at least four sessions of clinical practice with patients. | Lecture/Didactic Approach  Case Study Approach Mentorship Skill Practice |
| Tilson 2014 | Multiple Encounter Course | duration: 6 months  acrd: no aop: MSK | Physical therapist driven Education for Actionable Knowledge translation (PEAK) program – an educational program designed to promote physical therapists’ integration of research evidence into clinical decision-making at the individual and organizational level. 6 months in duration and consisted of four consecutive, interdependent components: 1) securing resources and leadership support; 2) a two-day training workshop; 3) guided small group work to develop a locally relevant list of evidence-based actionable behaviors – the “Best Practices List”; and 4) review, revision, and agreement to implement the Best Practices List. All components of the PEAK program supported a participant-driven learning experience: to work as a group to generate a Best Practice List around a common, participant-selected clinical area. The Best Practices List is a locally generated list of evidence-based, actionable behaviors that participants agreed (as a group) to implement in their clinical practice. Participants self-organized into small groups to review literature and generate evidence-based actionable behaviors. The actionable behaviors were reviewed and revised through a process of peer and expert review until all participants felt that they could implement the Best Practices List in practice [7]. | Case Study Skill Practice |
| Westervelt 2020 | Mentorship | duration: 3 x 1hr over 5 weeks acrd: no aop: MSK | Mentoring groups were assigned based on participant availability. All groups were composed of 1 clinical expert mentor and 2 mentee participants. 3 x 1-hour clinical mentoring sessions were held over 5 weeks using the Skype for Business videoconferencing platform.  Mentee participants selected and presented de-identified case studies of complex past or current patients with spinal dysfunction during each video- conference call. Mentees shared an outline of their selected patient case study with their mentoring group 2 days before their conference call to allow for all group members to familiarize themselves with the details of the case. Mentees identified major clinical questions they had regarding these patients and listed them beneath the case study. During the videoconferencing session, the mentor facilitated group discussion, promoted critical thinking, and presented current evidence regarding the case and clinical questions presented. | Case Study Approach Mentorship |
| Whitman 2020 | Residency and Fellowship | duration: 32.1 months(avg) acrd: yes aop: MSK | The American Board of Physical Therapy Residency and Fellowship Education (ABPTRFE) defines fellowship training (FT) as a ‘postprofessional planned learning experience comprised of a curriculum encompassing the essential knowledge, skills, and responsibilities of an advanced physical therapist within a defined area of subspecialty practice’   Region Specific Management Courses: Cervicothoracic, Lumbopelvic, Upper Extremity and Lower Extremity Delivery: 32 weeks in content role, 32 weeks as TA; faculty lead, online and immersive lab  Patient Management Framework and Advanced Clinical Decision Making Delivery: 18 weeks in content role, 18 weeks as TA; faculty lead online and immersive lab; weekly live 1-2 hour sessions  Fellowship Virtual Rounds Delivery: 18 weeks, faculty lead, online and immersive lab; weekly live 1-2 hours sessions  Pain Science and Psychosocial Implications in Musculoskeletal Care Delivery: 8 weeks, faculty lead  Introduction to OMPT and Professional Socialization; Mechanisms of Manual Therapy; Evidence-Based Practice and Writing Case Reports Courses | Lecture/Didactic Approach Case Study Approach Mentorship Self Reflection Skill Practice Student Assessment |
| Williams 2019 | Mentorship | duration: 150 hrs acrd: yes aop: MSK | The intervention was a 150-hour clinical mentorship programme aimed at facilitating clinical reasoning. The rationale for the intervention was drawn from the educational standards document of the International Federation of Orthopaedic Manipulative Physical Therapists (IFOMPT) a non-governmental federation promoting international excellence and unity in clinical and academic standards in the field of musculoskeletal physiotherapy; it is a subgroup of the World Confederation for Physical Therapy, which is a recognised partner of the World Health Organization. A minimum of 150 hours of mentored clinical practice is recomended for students, where the clinical mentor is a member of the member organization of IFOMPT. Furthermore, the clinical mentoring component of masters programmes that has been explored in terms of its impact on physiotherapist performance and career (as highlighted in the introduction) utilises this same educational approach. The intervention was delivered by mentors who were members of the Musculoskeletal Association of Chartered Physiotherapists (MACP), the UK member organisation of IFOMPT, having qualified at MSc / PhD level from a higher education establishment and who also had previous experience in delivering such mentorship at post-graduate level. The intervention took place in the usual clinical context of the participating physiotherapists, consisting of the mentors observing the participating physiotherapists assessing and treating new and follow-up patients, discussing and facilitating clinical reasoning processes immediately after the patients left the clinic. The model of clinical reasoning utilised for discussion was the dialectical model of Edwards et al [55] derived from qualitative studies of expert physiotherapists incorporating diagnostic and management aspects of clinical reasoning (such as narrative, collaborative and hypothetico-deductive reasoning) delivered over a sustained period, specifically to the learning needs of the clinician, with real time feedback | Mentorship Self Reflection Skill Practice Student Assessment |
