# Supplementary material for: The educational pathway to Advanced Practice for the physiotherapist: A systematic mixed studies review
Source: PLoS One. 2025 May 12;20(5):e0322626. doi: 10.1371/journal.pone.0322626 (PMC12068731; doi:10.1371/journal.pone.0322626)
Supplement: S4 Table — (DOCX) [file pone.0322626.s004.docx]

|  |  |  |  |  |  |  |  |  |  |  |  |
| --- | --- | --- | --- | --- | --- | --- | --- | --- | --- | --- | --- |
| Study ID | EP | Data Collection Tool | Pillars | Clinical Practice Pillar Supporting Text: | Qualitized | Leadership Pillar Supporting Text: | Qualitized | Education Pillar Supporting Text | Qualitized | Research Pillar Supporting Text | Qualitized |
| Adhikari 2020 | Single Encounter Course | Self‐administered questionnaire created by the authors: Clinical Decision Making Skills Assessment Tool (CDMSAT)  Section “A” is in the form of visual analog scale (VAS); 0 (no knowledge) to 10 (optimal knowledge). There are total 22 items to assess CDM. Items 1 to 14 assess knowledge, recent evidence, intervention selection, contextual factors, physiotherapy diagnosis, and use of reflective practice. Items 15 to 22 assess EP skills (a core component of CDM). The score of these 22 items can be analyzed individually or in total. The higher the total or itemized score better is the skills. The section ‘B’ is in the form of multiple‐choice and open questions to explore additional information from the participants  The CDMSAT was given to the participants to fill in before and immediately after the workshop | Clinical Practice | Each item and total Exercise Prescription (EP) score as well as the total Clinical Decision Making  (CDM) score demonstrated significant improvement in the VAS score after the workshop. The total CDM score yielded larger effect size (0.9) than those of any other individual item.  Between group analysis: - there was no group differences pre‐workshop between two levels of experience and between first and second workshops, both for EP and CDM - a significant difference was seen between BPT and MPT groups for CDM at pre‐training and both EP and CDM at post‐training  There was significant difference within the BPT group both for - EP (mean difference = 16.97, SD = 15.97, t = 5.82, P < 0.001) - CDM (mean difference = 53.40, SD = 28.26, t = 10.35, P < 0.001).  There was significant difference within the MPT group both for - EP (mean difference = 15.33, SD = 9.48, t = 5.60, P < 0.001) - CDM (mean difference = 38.83, SD = 20.65, t = 6.51, P < 0.001) | Qualitized: Improved clinical decision making including: assess knowledge, recent evidence, intervention selection, contextual factors, physiotherapy diagnosis, and use of reflective practice and capacity to prescribe exercise  1.3 1.6 1.7 1.11 |  |  |  |  |  |  |
| Allison 2023 | Single Encounter Course | electronic questionnaires at baseline and 6 weeks post randomization. To evaluate the effectiveness of the program, a custom self-report tool (RIVA questionnaire) was developed by the researchers  The primary outcome was self-reported confidence in knowledge about weight management for patients with OA   Secondary measures include self-reported confidence in clinical skills for weight management for patients with OA, self-perceived competence in nutrition care to patients with chronic disease (NUTCOMP) questionnaire and weight stigmatized attitudes (Anti-Fat attitudes questionnaire)  Participants were asked to rate their confidence on a 5-point Likert style scale where 1 = Not at all confident, 2 = Not very confident, 3 = Somewhat confident, 4 = Confident, 5 = Very confident responding to the knowledge and clinical skills statements. | Clinical Practice | The education group demonstrated a greater mean increase in confidence in knowledge of weight management at 6 weeks compared to the control group (adjusted between-group mean difference RIVA-A (95% confidence interval (CI)), 22.6 (19.6, 25.5)).  For the secondary outcome measure of confidence in clinical skills, the education group’s mean confidence improved at 6 weeks, while the control group demonstrated no change (adjusted between group mean-difference RIVA-B (95%CI), 14.6 (12.7, 16.4)).  Self-reported competency in nutrition care as measured by the NUTCOMP questionnaire, showed a greater improvement in the education group compared to the control group (adjusted between group mean-difference (95%CI), 39.9 (34.5, 45.4)).  Weight stigmatized attitudes, as measured by the Anti-Fat attitudes questionnaire total score at 6 weeks, improved in the education group compared to the control group (between group mean difference (95%CI) -6.5 (-11.4, -1.6)) | Qualitized: Improved confidence in knowledge and clinical skills. Altered attitudes re weight stigmitization (understanding population health needs).  1.10 1.11 |  |  |  |  |  |  |
| Anderseck 2020 | Accredited Area of Practice Training | Questionnaire posted on facebook and sent via email centring on what advantages and disadvantages does this specialization bring, what changes can be noticed after completing the further training and how does this affect patient satisfaction? | Clinical Practice Education Research | The respondents' perception of more detailed anamnesis and diagnostic skills (99.5% agreement), higher patient satisfaction (subjective feeling of the therapist) (84.2% agreement) or better advice for patients (98% agreement) clearly positive. There was also a high level of agreement for achieving increased self-confidence (97.9% agreement) and a better view of the entire.  For 44.7% of those surveyed, the patient clientele has changed, as more patients are now coming to them with complex and difficult problems. | Qualitized: Improved ability to take detailed hx and enhanced diagnostic skills. Feeling more confident in their work and having a broader perspective on patient care. Shift in patient base, more presenting with complex and difficult problems  1.4 1.6 |  |  | In the present study it was found that 17.4% of those surveyed in Germany work in school and further education and in a physiotherapist practice | Taking on education roles as well as clinical roles.   3.8 | In this work, 2.1% of those surveyed stated that they worked in practice and in research, and another two people (1.1%) stated that they worked exclusively in the field of research. There are also 5 OMT therapists who work in research and also in other areas of physiotherapy. | Working in research roles as well as clincal roles.   4.1 |
| Balogun 2018 | Single Encounter Course | The Professionalism Inventory evaluation tool was administered before the professionalism educational intervention lecture and was re-administered 1 hour after the lecture.  this standardized Professionalism Inventory that has been found among Nigerian physical therapists to be highly stable and internally consistent when re- administered within two-week testing intervals.  The knowledge of professionalism scale has 10 questions designed to assess respondents’ basic understanding of professionalism. Each question has a Yes, No, or Don’t know response option. The attributes of the professionalism scale has respondents rate their opinions on 16 professional behaviors and attitudes on a five- point Likert scale ranging in a continuum from Strongly Disagree (1) to Strongly Agree (5). The 16 attributes of professionalism, perception-based statements have 8 subscales: clinical competence, a spirit of inquiry, accountability, autonomy, advocacy, innovation and vision, collegiality and collaboration, and ethics and value. For knowledge of professionalism, the minimum score is 0, and the maximum possible score is 100; the attribute of professionalism minimum score is 16, and the maximum possible score is 80. | Clinical Practice | The educational intervention did not significantly (p > .05) improve the aggregate attributes of professionalism score. However, an in-depth analysis of the professionalism subscales data indicated that the intervention score significantly (p < .05) improved some attributes of professionalism. Clinical competence, accountability, autonomy, innovation and vision, and collaborating and collegiality improved significantly (p < .05) | Qualitized: Some aspects os professionalism may have improved, or at least knowledge of professionalism in the categories of clinical competence, accountability, autonomy, innovation and vision and collaborating and collegiality.  1.1 1.2 1.11 |  |  |  |  |  |  |
| Banks 2013 | Accredited Area of Practice Training | Semi-structured questionnaire  1. Have you achieved the learning outcomes you identified at the beginning of the academic year? 2. Do you feel the three modules have supported your personal CPD over the year? 3. Have you been able to design your clinical assistance sessions around the learning outcomes from the modules? 4. Have you carried out any self-directed learning activity related to the modules learning outcomes? 5. Has the CPD programme [modules, clinical assistance, self-directed learning] had an influence on your PDA and KSF outline? 6. Has your practice changed over the last year because of the CPD programme? 7. How would you change or modify the programme for the next academic year? 8. Would you be happy to receive feedback on your clinical practice again to help identify learning outcomes for 2011-12?  Competency Performance Rating (Pre and Post intervention) The sessions included approximately 45 minutes for the participant to examine and treat a new patient whilst being observed by one of seven MACP colleagues. All assessors had experience in evaluating clinical competencies at a postgraduate level and worked for the Trust. The allocation of participants to assessors was not random, but was based on their working locality for convenience. The observation of practice was followed by 15 to 20 minutes of discussion about the participants clinical rea- soning competencies and 15 to 20 minutes of feedback on their performance based on 20 prior-identified competencies. Each of the competencies matches one of ten dimensions from within the IFOMPT educational standards document. A visual analogue rating scale of performance in each competency was used to enhance written feedback. Data for each competency was based on measurement, in centimetres, of a line or cross-placed on the visual rating scale by the mentor. The rating scale was designed to give feedback to participants on where they were performing clinically on a 15-cm measurement scale between a novice practitioner with 0-cm (0%) competency and an expert with a 15-cm (100%) competency  Analysis of a convenience sample (n=20) was made of the differences between the groups of data for each competency. The aim was to deter- mine whether there were improvements in clinical perform- ance and if so, whether the improvements were significant. | Clinical Practice Research | Results show that, overall, there was an improvement in 17 out of 20 competencies when comparing performance rating pre- and post-intervention (Figure 2). The greatest improvement came in the range of handling skills demonstrated (14.6% increase in visual analogue rating score), and the least in the process of reassessment (0.7%).   Communication improved by 6%, clinical reasoning by 4%, functional analysis of movement by 6%, structural differentiation by 12%, range of manual handling skills by 14.6%  96% of respondents felt that their practice had changed positively over the year because of the programme. | Qualitized: Improved handling skills, communication and clinical reasoning.  1.6 1.7 1.11 |  |  |  |  | the use of research in practice by 4%, and critical appraisal of evidence by 4.6% | 4.3 |
| Barton 2021 | Single Encounter Course | Following informed consent [Ethics approval provided by La Trobe University, S16-51], and prior to training, an investigator-developed survey was administered (Additional file 2). The survey, developed by the research team (CJB, JLK, JW, NL and KMC), was informed by clinical practice guidelines [8–10] and the theoretical domains framework (TDF) [40,41]. Questions explored (i) current practices when treating knee osteoarthritis; and (ii) confidence and be- liefs about capabilities to provide patient education and exercise-therapy to people with knee osteoarthritis. This survey was repeated 1-2-weeks post-training completion. A further repeat of the survey at 12-months post-training, sent to 775 physiotherapists and completed by 147 (19%), also explored implementation barriers and enablers via open-ended questions | Clinical Practice Leadership | Health professional: Training had a small effect on the number of physiotherapists discussing treatment goals, prescribing neuromuscular exercise, using supervised exercise, and discussing the importance of weight management (ES = 0.10–0.26) all or most of the time (Table 3).  Training had a moderate effect on the proportion of physiotherapists believing they had been trained to deliver guideline-recommended education and exercise-therapy (ES = 0.46), and small effect on perceived knowledge and skills (ES = 0.27) to do so (Table 4). Training had a large effect on the proportion of physiotherapists confident to prescribe neuromuscular exercise (ES = 0.51); and moderate effect on the proportion of physiotherapists confident to provide education and exercise therapy following guidelines when a patient is not motivated, provide education related to self-management and physical activity, and discuss weight management (ES ¼ 0.30–0.43) | Qualitized: Improved capacity to discuss Rx goals, presrcibe exercise, discuss weight management with OA population. Improved confidence of PTs to educate patients and follow guidelines.  1.5 1.7 1.10 1.11 | Health professional: 91% (337/371) of physiotherapist post-training survey respondents had implemented GLA:D® or intended to imple- ment in the next 6-months. 79% (116/147) of physiotherapists who responded to the 12-month survey had implemented GLA:D® | Lead new practice and service redesign in implimenting GLAD.   2.6 2.7 |  |  |  |  |
| Bastick 2020 | Multiple Encounter Course | Kirkpatrick’s hierarchical model was used as the basis to evaluate the effect of this education program (Kirkpatrick and Kirkpatrick, 2009). Kirkpatrick’s model outlines four levels of educational evaluation: 1) participant reaction (e.g., satisfaction or happiness); 2) participant knowledge (knowledge or skills required); 3) participant change in behavior; and 4) change in health outcomes (Hutchinson, 1999; Kirkpatrick and Kirkpatrick, 2009). Outcomes in this study were measured at Levels 1, 2 and 3. Level 1 outcomes (participant reaction) were measured by collection of descriptive comments through survey at the completion of each 8-week education block. The Level 2 outcome (knowledge) was measured using self-reported self-efficacy in stream-specific learning objectives pre and post each 8-week training block (see Intervention for more details). Self-efficacy was measured on a 0–100 scale where 0 = cannot do (the task/objective) at all, 50 = moderately certain can do and 100 = highly certain can do (Bandura, 2006). Self-efficacy was selected as a proxy for behavior on the basis that self-efficacy is an important indicator of future behavior (Bandura, 2006; Delany et al, 2015; Holdsworth, Skinner, and Delany, 2016). This was particularly important given the resources required to observe actual clinician behavior for such a large group of physiotherapists, which would have also involved observation across each of the clinical streams, would have been substantial and were not available for this study. Level 3 outcomes (i.e., clinician behavior) were assessed using self-rated independence and self-rated ability to independently work weekend shifts in specific clinical areas. Self-rated independence in stream-specific clinical practice was measured by junior staff using a 4-point Likert confidence scale as follows: 1) not confident; 2) confident with close supervision (i.e., another physiotherapist supervising in the direct area); 3) confident with distant supervision (i.e., another physiotherapist available via phone); or 4) independent. This was measured to assess whether the training program had any effect on clinician perceived practice behavior. Self-rated ability of junior staff to work stream-specific weekend shifts was also measured using a ‘Yes/No’ rating, to assess whether the training program affected in any change in practice behavior of relevance to the department (i.e., increased workforce capacity to meet the demands of a 7-day service) | Clinical Practice | Self-efficacy Self-efficacy improved in many objectives following training in all clinical streams (Table 4). The mean improvement in self-efficacy across objectives ranged from 2.9 (95% CI −8.7 to 14.5) to 43.3 (95% CI 4.8–81.8) points, and there was significant improvement in self-efficacy (p < 0.05) for 41/51 (80%) of objectives (Table 4)  Clinician independence and ability to work weekends Self-rated independence scores improved at an individual level for 45.6% of stream-specific learning objectives; 52.8% were unchanged and 1.7% reported a decrease in perceived independence (i.e., in 45.6% of objectives, individuals ranked their self-rated independence at a higher category post-training). However, only one objective demonstrated significant improvement in work readiness; the ability to work on the wards with a deteriorating patient with cystic fibrosis in the cardiothoracic stream (Tables 5 and 6). Several objectives could not have a p-value calculated as no therapists crossed the threshold from not work ready (categories 1 and 2) to work ready (categories 3 and 4) (Table 6). There was a significant relationship between self-efficacy and physiotherapist’s perceived level of independence and confidence to work in practice settings, p < 0.001 (Figure 1). This relationship was consistent after training, with a demonstrable link between improved self-efficacy following training and self-rated independence of practice (Figure 2, p < 0.001). Self-rated ability to work stream-specific weekend shifts increased from 56–70%. However, no stream achieved a statistically significant increase in staff able to work independently during weekend shifts (p range 0.10–1.0) | Qualitized: Increase in self efficacy and self-rated independence, demonstrating understanding of responsibility and autonomy.   1.2 |  |  |  |  |  |  |
| Bird 2022 | Single Encounter Course | The final stage of the study focused on evaluating the efficacy of the workshops to impact staff health literacy  and client management via survey and interview. This involved the re-administration of a Health Literacy  Knowledge, Skills and Experience Questionnaire three weeks after the final workshop and impact evaluation of the workshops by interviews after the workshops.   Post-intervention experiential surveys and phone interviews explored the physiotherapists’ perspectives on perceived benefits of engaging in this project,challenges to implementation of new knowledge, and actual and planned changes in client management. Each interview was audio-recorded, transcribed, and thematically analyzed (Braun, Clarke, Hayfield, and Terry, 2019). Each participant was given a unique identifying letter and  number combination for quotes. | Clinical Practice Leadership Education | Quantitative: Significant differences were observed in the pre and post comparison for 14 out of 15 questions. The findings are also presented by domains; significant differences were observed for both knowledge and skills domains (all p < .001). Improvements for these domains are large; 63% and 65%, respectively. For both domain changes the effect size was 2.7 which is a large effect.  Qualitative: Theme two: changing practice to promote understanding described several tools (strategies) that they had learned about to check understanding and how they changed their practice as a result i.e teach-back was described as a potent tool for practice change: “[I have been] using the teach back model, and often finding that the inconsistency between what I’ve sort of discussed for the session and what they’re sort of understanding, so it’s a really powerful tool in that regard”.  described the way they had focused more on listening and tailoring the health information to the needs of the clients, rather than trying to impart professional knowledge routinely. Two examples that demonstrate this: “just looking at our listening skills, . . . allowing them to ask questions and be able to express their understanding of their health condition” (P4) - “so that we can start from where they’re at the moment and just give them little bits of info and checking whether they understand it.” (P3)  Awareness of the importance to use plain language as part of practice change was described by this participant: “incorporating more awareness of when I’m using jargon and how to write things in a more easily understood and generic manner”. (P10)  Within the physiotherapists’ reflections there was evidence of a change to having a more person-centered approach: “my starting different conversations with clients of, “What do you need from me today?” (P10).  The physiotherapists described a process of self- reflection that focused on their role in promoting client understanding:“Instead of trying to make them think that we’re testing them, but actually try and just clarify that I’ve taught them correctly. So, it’s more of a reflection on me than them.” (P6). | Qualitized: PTs demonstrated improved knowledge and skills re health literacy.  1.3 1.5 1.10 1.11 | Theme three: reflection on roles within the clinic These data highlighted the participants’ learning about  the attributes of a health literacy responsive organization  (clinic), including fostering effective communication  practices and building the capability of the health pro- fessionals (physiotherapists). Thus, the data within this  theme can be viewed on two levels: 1) at the level of the  clinic; and 2) at the individual practitioner level. Within  the responsibility of the whole clinic, increased health  literacy awareness is described: “I think as a practice [clinic], we’re just more aware of it on a whole”. (P2) This awareness produced tangible changes to the way in which information was provided to people including via the Internet: “in terms of how we write our blog posts and our website  design and our pamphlet design, we’re starting to be a bit more considerate, due to participating in this study” (P3) and in written material: “looking at how to construct an information brochure for  clients, what that would look like, and at what level it’s  best to do that so that clients get the most benefit out of  those.” (P4) The physiotherapists described a process of self-reflection that focused on their role in promoting client understanding: “Instead of trying to make them think that we’re testing  them, but actually try and just clarify that I’ve taught  them correctly. So, it’s more of a reflection on me than  them.” (P6). Within the physiotherapists’ reflections there was evi- dence of a change to having a more person-centered  approach: “my starting different conversations with clients of, “What do you need from me today?” (P10). | 2.2 2.5 2.9 | Theme one: the multi-faceted nature of health literacy requires multiple strategies - The interviewees described an understanding that health literacy is multi-faceted and varied. For example: “it’s influenced by a whole number of factors in terms of the environment that you’re in, the behaviour of the practitioner, the information that’s provided by the practitioner,” (P1). recognizing the importance of client experiences with health professionals and the health system in determining current health literacy profile:“so, more on their exposure to health providers and their understanding of health terms and use of health facilities and . . . their previous exposure”. (P10) knowledge gained through the workshops was used by the physiotherapists to respond to individual health literacy needs “it’s definitely given me some insight into how varying different people’s health literacy may be and that I have to be accommodating and adaptable” (P1). Clinicians described translating this new knowledge about health literacy diversity into practice by responding to varied client needs by providing information in multiple formats. One example is: “I write things down a lot more, use diagrams. Yeah, I try to include as many different forms of communication in my sessions ́. (P6) Qualitative: Theme two: changing practice to promote understanding described several tools (strategies) that they had learned about to check understanding and how they changed their practice as a result i.e teach-back was described as a potent tool for practice change: “[I have been] using the teach back model, and often finding that the inconsistency between what I’ve sort of discussed for the session and what they’re sort of understanding, so it’s a really powerful tool in that regard”. described the way they had focused more on listening and tailoring the health information to the needs of the clients, rather than trying to impart professional knowledge routinely. Two examples that demonstrate this: - “just looking at our listening skills, . . . allowing them to ask questions and be able to express their understanding of their health condition” (P4) - “so that we can start from where they’re at the moment and just give them little bits of info and checking whether they understand it.” (P3) Awareness of the importance to use plain language as part of practice change was described by this participant: “incorporating more awareness of when I’m using jargon and how to write things in a more easily understood and generic manner”. (P10) | 3.3 3.7 3.8 |  |  |
| Brennan 2006 | Multiple Encounter Course | Clinical outcome of patients with neck pain were based on the change in NDI scores, rate of change per visit for the NDI, and achieving a minimum detectable difference in NDI scores.  The number of physical therapy visits, length of stay, and total physical therapy charges also were recorded for each patient.  The effectiveness of the 2-day CE course was examined by comparing the clinical outcomes of patients who were treated by attending or nonattending therapists during the year preceding the CE course (pre-course period) with the outcomes in the year following the CE course (post-course period) | Clinical Practice | The ANCOVAs comparing clinical outcomes between participants and nonparticipants, adjusted for age, sex, and baseline NDI and pain scores, revealed a significant interaction between time (pre- or post-course) and participation when NDI change scores were the dependent variable (P.037, mean difference4.03, 95% confidence interval1.30 – 6.76) (Tab. 5). The interaction effect on NDI change scores is graphed in Figure 5, indicating that therapists who attended the CE course and who participated in the clinical improvement project showed improved clinical outcomes from the pre- to post-course period, whereas therapists who attended the CE course and who did not participate in the clinical improvement project experienced a decrease in clinical outcomes over the same time period.  The participant therapists were able to improve their clinical outcomes for patients with neck pain while maintaining significantly lower median physical therapy charges than nonparticipant therapists, suggesting improved cost-effectiveness of care. | Qualitized: Improved clinical outcomes for patients, and less cost to the patient after engaging in the course.   1.7 1.11 |  |  |  |  |  |  |
| Briggs 2023 | Residency and Fellowship | Eight self-perceived clinical competency questions were developed based on the Dreyfus19 model of adult skill acquisition and refined by the study team and focus groups. Questions asked the respondents to rate their level of agreement or disagreement with each statement on a 5-point Likert scale.  The survey also included a section with focused questions measuring the percentage of time spent on various job duties/responsibilities in a typical week. Included 6 items: “patient care (including documentation),” “administration,” “research,” “clinical teaching/ mentoring” “teaching in academia,” and “other.” | Clinical Practice Education Research and Development | Self-Perceived Clinical Competency Results from the Kruskal–Wallis test comparisons demonstrated significant differences across groups across all clinical competencyquestions except relying “heavily on clinical practice guidelines when treating patients.” Pairwise post hoc tests demonstrated that the residency-trained group reported higher clinical competency scores (greater agreement) compared with the non–residency-/fellowship-trained group regarding responsibility for patient progress (mean [SD]: 3.4 [0.7] vs 3.1[0.9]; P = .007) and importance of reflection (4.4 [0.8] vs 4.2 [0.7]; P < .001; Table 2). All other questions did not show a significant difference between residency-trained and non–residency-/fellowship-trained groups (Table 2). fellowship-trained group scored higher (agreed more) compared with the non–residency-/fellowship-trained groups with the following:  1) being comfortable with ambiguity/uniqueness in patient care (4.3 [1.1] vs 3.9 [1.0];P < .001) 2) patient failure being their responsibility (3.4 [0.9] vs 3.1 [0.9]; P < .001) 3) the importance of reflection (4.7 [0.6] vs 4.2 [0.07]; P < .001) 4) their skill performance (4.3 [1.0] vs 4.2 [0.8]; P < .001).  Conversely, the fellowship-trained group compared with the non–residency-/fellowship-trained group scored lower (disagreed more) with the following:  1) relying heavily on protocols when treating patients (2.4 [0.9] vs 2.9 [1.0];P < .001) 2) feeling overwhelmed by my patient care and workload (2.4 [1.1] vs 2.7 [1.1];P < .001) 3) having difficulty prioritizing and organizing patient care needs (1.7 [0.8] vs 2.0 [0.9]; P < .001; Table 2). | 1.1 1.2 1.3 1.5 1.11 |  |  | Weekly Job Responsibilities Results from the Kruskal–Wallis test comparisons also demonstrated significant differences across groups across all job duty/responsibility items except “Other” (Table 3). residency-trained group reported less time spent in patient care (75.8% [28.5] vs 78.2% [29.9]; P = .02) and greater time spent in research (3.8% [12.6] vs 1.5% [7.6];P < .001), clinical mentoring (4.5% [7.1] vs 3.1% [6.8]; P = .02), and teaching in academia (6.5% [20] vs 2.9% [13.5]; P < .001) compared with the non–residency-/fellowship-trained group, respectively (Table 3). fellowship-trained group also reported less time spent in patient care (58.4% [35.9] vs 78.2% [29.9];P < .001) compared with the non–residency-/fellowship-trained group. e fellowship-trained group reported greater time spent with: 1) administrative duties (16.9% [22.9] vs 12.1%[23]; P < .001), 2) research (5.1% [12.6] vs 1.5% [7.6]; P < .001), 3) clinical mentoring (6.9% [9.9] vs 3.1% [6.8]; P < .001), and 4) teaching in academia (10.9% [22.3] vs 2.9% [13.5]; P < .001) compared with the non–residency-/fellowship-trained group (Table 3). Finally, differences between the residency-trained group and the fellowship-trained group were present for several items. fellowship-trained group reported greater time spent with: 1) administrative duties (16.9% [22.9] vs 7.2% [23]; P < .001), 2) research (5.1% [12.6] vs 3.8% [12.6]; P = .01), 3) clinical mentoring (6.9% [9.9] vs 4.5% [7.1]; P = .005), and 4) teaching in academia (10.9% [22.3] vs 6.5% [20]; P < .001) compared with the non–residency-/fellowship-trained group (Table 3). | More residency and fellowship trained physios work in clinical mentoring and teaching in academia than those who have not done the training.  3.8 | Weekly Job Responsibilities Results from the Kruskal–Wallis test comparisons also demonstrated significant differences across groups across all job duty/responsibility items except “Other” (Table 3). residency-trained group reported less time spent in patient care (75.8% [28.5] vs 78.2% [29.9]; P = .02) and greater time spent in research (3.8% [12.6] vs 1.5% [7.6]; P < .001), clinical mentoring (4.5% [7.1] vs 3.1% [6.8]; P = .02), and teaching in academia (6.5% [20] vs 2.9% [13.5]; P < .001) compared with the non–residency-/fellowship-trained group, respectively (Table 3). e fellowship- trained group also reported less time spent in patient care (58.4% [35.9] vs 78.2% [29.9]; P < .001) compared with the non–residency-/fellowship-trained group. e fellowship-trained group reported greater time spent with: 1) administrative duties (16.9% [22.9] vs 12.1% [23]; P < .001), 2) research (5.1% [12.6] vs 1.5% [7.6]; P < .001), 3) clinical mentoring (6.9% [9.9] vs 3.1% [6.8]; P < .001), and 4) teaching in academia (10.9% [22.3] vs 2.9% [13.5]; P < .001) compared with the non–residency-/fellowship-trained group (Table 3). Finally, differences between the residency-trained group and the fellowship-trained group were present for several items. e fellowship-trained group reported greater time spent with: 1) administrative duties 16.9% [22.9] vs 7.2% [23]; P < .001), 2) research (5.1% [12.6] vs 3.8% [12.6]; P = .01), 3) clinical mentoring (6.9% [9.9] vs 4.5% [7.1]; P = .005), and 4) teaching in academia (10.9% [22.3] vs 6.5% [20]; P < .001) compared with the non–residency-/fellowship-trained group(Table 3). | Residency and fellowship trained physios spend more time in research compared to those not trained.   4.1 |
| Camden 2015 | Single Encounter Course | Three questionnaires were used (pre-, post- and follow-up), with each containing items related to three variables: self-reported knowledge, skills and EBP. To compare changes in PTs’ self-reported knowledge and skills, the same items were included in all three questionnaires (see questionnaire items in Tables 2 and 3).   Items were adapted from previous questionnaires that evaluated DCD beliefs,  knowledge, and skills in occupational therapists. These items were reviewed by expert DCD therapists, and have been used in multiple studies since 2004 (e.g. Missiuna et al., 2012). Each item used a 7-point  Likert scale for responses. In order to describe the sample, background information was collected in the pre-questionnaire (e.g., work setting, number of children with DCD seen per week). In order to document EBP, closed- and open-ended questions were also included in each questionnaire | Clinical Practice Research | Overall, participants’ perceptions of the usefulness of the module in increasing their knowledge and skills were high, with mean scores of 6.0 (SD:1.1) and 5.5 (SD:1.0), respectively (n=49). Specifically, 79% of the items evaluating self-perceived knowledge and skills increased significantly following  module completion  Many felt better equipped to help families despite limited resources  The themes of intended behavior change were quite varied and included: 1) trying to better identify children who might have DCD; 2) use of assessment tools; 3) involving the family and the child more in goal setting; 4) changing their focus of intervention (e.g., participation instead of impairment, increasing capacity-building with teachers and community partners); and 5) sharing more information with families, colleagues, physicians, teachers, and community partners  The increase in knowledge and skills found immediately after module completion was maintained for 24 items out of the 27 that were significantly increased in the post questionnaires compared to the pre questionnaires | 1.4 1.5 1.6 1.7 1.9 1.11 |  |  |  |  | Overall, participants’ perceptions of the usefulness of the information and resources contained within the module in supporting them in providing EBP was high, with a mean score of 6.2 (SD:0.8) (n=49).   Following module completion, almost all participants (92%) reported feeling more confident in providing EBP.   “I knew already about the best evidence but struggled a little as to how to apply these principles. I feel the videos provided a great resource to this application"  Approximately one third (34%) reported having changed one or more aspects of their practice following module completion. General themes noted as influencing PTs’ ability to provide EBP included having access to resources such as evidence-based information, handouts, and site ‘champions | 4.3 |
| Carr 2020 | Mentorship | Two phases of data generation were completed for this study within the participant’s workplace, both led by the lead researcher. The initial phase comprised of eight individual one hour interviews. Two patient forums and a pilot interview informed the development of an initial interview guide (available online – appendix B) An intensive interviewing style was adopted throughout all interviews to allow the researcher to conduct a broad exploration of the area of study and facilitate the researcher in focusing the interviews towards developing  topics of interest (Braun and Clarke 2013). A process of concurrent data generation and analysis was completed following each interview to identify emerging codes and categories which required further exploration in subsequent interviews. This process supported the development of iterative interview guides to ensure questioning became more focused to prevalent and significant emerging codes to facilitate progressive development of conceptual categories (Birks and Mills 2015) The second phase of data generation comprised of secondary one hour interviews with two participants who had completed initial interviews and a subsequent two hour focus group. Theoretical sampling informed selection of participants for this phase of data generation sampling participants on their ability to generate data to saturate developing categories (Charmaz 2014)The two participants (P2 and P3) sampled for secondary interviews were  selected to explore unsaturated developing categories they had discussed in their initial interviews. Three participants (P9, P10 and P11) who had not completed initial interviews were sampled in addition to all original participants to partake in the focus group to ensure additional insights could be captured. Three participants (P3, P7 and P8) were unable to attend the focus group due to work commitments. Due to time constraints theoretical sampling for phase two generation remained within the single participant cohort. Data generation ceased at this point as the researcher and research supervisor considered sufficient data saturation had been achieved. Subsequent analysis of these data concluded that no new properties were added to the developing categories and that the identified properties provided adequate meaning for patterns within the data, representing therequirements for data saturation in grounded theory research (Glaser 1978, Charmaz 2014, 2006). All episodes of data generation were digitally recorded and transcribed verbatim by the researcher. Transcripts were returned to participants to review for accuracy prior to analysis. A reflexive journal was maintained by the lead researcher throughout the data generation process to document the influence of their prior knowledge and opinions on the data generation process. Regular researcher memos were also documented to demonstrate how their interpretations of the emerging data affected the analysis processes and subsequent data generation. | Clinical Practice Leadership Education | Observed clinical practice situated in the workplace has been presented as an appropriate learning activity to facilitate the development of clinical expertise. The findings of this study have demonstrated how observed practice allows the learner to demonstrate, and the mentor to observe, a range of knowledge types required for clinical expertise. Epistemic or scientific knowledge is recognised as invariable knowledge that is “technical and rational” and traditionally grounded in positivistic inquiry. This form of knowledge is recognised as an aspect of physiotherapy practice that we are professionally well developed in (Jensen 2011). This study recognised how regular workplace observed practice with mentor feedback allows the mentor to view the application of such knowledge by the learner within the observed clinical practice. The mentor is able to cite epistemic knowledge within their feedback, relevant to the clinical presentation observed, and the learner is able to show application of this knowledge in subsequent observed practice sessions.  Professional craft knowledge is described as knowledge gained through reflection on professional practice experience (Higgs and Titchen 2001). It considers a clinician’s level of phronetic knowledge by recognising their ability to integrate epistemic knowledge to the context of the care to be provided, considering how practical issues will affect this care.   Mentors in this study identified how observing clinical practice enables them to witness a learner’s ability to dynamically consider and apply these forms of knowledge to individual episodes of care. | 1.11 | “I think (the approach)…empowers the team…because all of them have to go through it, they all know what it feels like to go through it…they are more likely to feedback and then…the learner willthen help others to learn.” – P8(M) | 2.4 | “I think having this in place…create(s) an environment where we are a team that wants to continue with learning and development,…people are more encouraged to naturally want to learn in between those watched assessments” – P2(M) | 3.2 3.4 3.5 |  |  |
| Cheema 2022 | Residency and Fellowship | Outcomes Database  Swinkels et al., 2007 A retrospective observational cohort design examining data from a large national  database including patient demographics, health characteristics, and outcomes using the  Patient Inquiry software developed by Focus on Therapeutic Outcomes (FOTO), a Net  Health company that provides outcomes management software solutions for rehabilitation  therapists. FOTO is a commercial patient outcome assessment system that is used across the United States. The database contains patient outcome data from a variety of settings, and includes demographic information and protected health information while using computerized adaptive tests (CATs) to collect and track outcomes in the form of a functional status (FS) score  Clinician Survey FOTO does not gather clinician-training demographics about subscribers, so a preliminary survey using e-blasts with collaboration from several professional organizations was necessary to recruit clinicians. The web-based survey (Appendix A) was created with the assistance of researchers that have had to employ a similar survey in the past including a former director of an accredited orthopedic physical therapy residency program and FOTO researchers. The headline of the invitational e-blast included “Are you a therapist who used FOTO for at least 10 completed patient episodes for low back pain anytime between 2015–2017? | Clinical Practice Leadership | Once adjusted for propensity scores, those without advanced training showed an insignificant 0.5 point smaller change in FS compared to clinicians with the advanced training (p=0.3551)  The clinicians with post-professional training (residency and fellowship) showed an insignificant 0.4–0.5 point increase in FS change (after propensity adjustments) compared to the neither group, but this difference is not likely to be clinically meaningful (Wang et al., 2010). | Qualitized: Residency and fellowship training showed some correlation with improved patient outcome scores compared to those without; though not necessarily clinically meaningful.  1.11 | In our study, using a logistic regression technique, we saw an insignificant 0.1 point difference (lower) in FS score change when the treating clinician did not have post-professional training, but this difference increased to 0.5 points after the propensity score adjustment. This is an indicator that those with advanced training are in fact being referred patients who are more clinically complex.  It is also worth noting that those who use a subscribed outcomes database may not accurately reflect the typical training and priorities of most PTs. For example, an average of 71.12% of survey responders had OCS certification, while overall only 6% of U.S. PTs have this certification(Specialists, 2021). It can be argued that those with higher levels of training are inherently high-achievers and more likely to see value in tracking patient outcomes and are more prone to utilize standardized outcome documentation systems | 2.3 2.7 2.8 |  |  |  |  |
| Chipchase 2016 | Single Encounter Course | Practice behaviour was measured with a purposively designed, semi-structured questionnaire containing closed (Likert-type re-sponses) and open questions. The questionnaire was developed inan iterative process based on previously published questionnaires (Grant and Niere, 2000; Hurley et al., 2002), the expertise of the project team, feedback from previous workshops and pilot testing on a sample representative of the study population. The ques- tionnaire had two sections and questions are presented within the tables of results (Tables 2 and 3, respectively). Briefly, section one included questions related to practitioner confidence in the assessment of cervical motor and sensorimotor function, as well as the prescription and progression of exercise in the management of patients with neck pain. Category codes were: 1 ¼ not confident; 2 ¼ somewhat confident; 3 ¼ confident; 4 ¼ very confident. Section two gathered information on their usual management strategies for patients with neck pain. Category codes were: 1 ¼ not at all; 2 ¼ some of the time; 3 ¼ most of the time; 4 ¼ all of the time. Questionnaires were completed by all participants prior to the first weekend workshop and again four weeks after the final follow-up workshop. The four-week intervals between the initialworkshop, follow-up workshop and final assessment were selected to provide participants with sufficient opportunity to adapt their practice behaviours and treat an adequate number of patients (Bekkering et al., 2005; Brennan et al., 2006; Cleland et al.,2009). Questionnaires were mailed to participants with a Reply Paid envelope. Reminders and follow-up phone calls were made to enhance the response rate.Clinical outcomes achieved by participants were assessed with the Neck Disability Index (NDI), which is a reliable and valid outcome measure for patients with neck pain (Vernon, 2008). Patient data were collected on two occasions. First, on enrolment into the study (two months prior to the workshop), all participants were asked to administer the NDI to 10 successive neck pain patients on the first day of treatment and then again at the fourth occasion of presentation to the physiotherapist. All participants were asked to repeat this again on another 10 successive neck pain patients after completion of the follow-up educational intervention (two months after the two-day program). The patient outcome data was collected this way to measure the clinical effectiveness of the educational intervention before and after the workshop/s following three treatments. This provided consistency in the outcomes of a defined amount of treatment, rather than a variable amount of treatment as would occur if data were collected at the initial treatment and at discharge | Clinical Practice | Section 1 Participants were asked to report their confidence in being able to perform a number of examination and management techniques for patients with neck pain disorders. Table 2 presents the mean scores or each question for both the intervention and control group before and after the intervention. While both groups significantly improved in all areas from baseline, no significant between-group differences were identified for any of the Likert scale responses (see Table 2). When subsections of the confidence questionnaire were totalled, no significant differences were observed between the intervention and control in terms of assessment (F = 0.12, df = 1, p = 0.73, n 2 = 0.01 [95% CI -0.27 to 0.29]), clinical reasoning (F = 0.71, df = 1, p = 0.41, n2 = 0.04 [95% CI -0.34 to 0.42]), or progression of exercises (F = 0.02,df = 1, p = 0.90, n2 = 0.01 [95% CI -0.79 to 0.81])  Section 2 Participants were asked to consider their last ten patients with neck pain/dysfunction and to report how often they used a range of management strategies. Table 3 presents the mean scores for the frequency with which participants reported theiruse of a variety of management techniques in their usual man- agement of neck pain patients. The focus of most management for neck pain prior to the education intervention included manual therapy, postural re-education, ergonomic advice and home exercise programs. Following the educational intervention, there was no change in practitioners' reports of the frequency with which they used any of these management strategies (p > 0.05)  Patient outcomes data Although reminders were sent to participants, not all practitioners collected data on 10 successive patients before and after the educational intervention. The participants collected a total of 158 patients with complete NDI data before the intervention and 115 after the intervention (Table 4). NDI scores were calculated out of 50. Changes in NDI data over time were calculated and then aver- aged for each participant. There was no effect of TIME (F = 0.45, df = 1, p = 0.51, n2 = 0.00 [95% CI -0.04 to 0.04]) or GROUP (F = 0.36, df = 1, p = 0.56, n2 = 0.00 [95% CI -0.04 to 0.05]). In addition, there was no significant TIME*GROUP interaction. This meant that there were no differences between groups in terms of patient outcomes as measured by the NDI before and after the educational in- terventions (Table 1; F = 2.88, df 1, p = 0.11, n2 = 0.03 [95% CI -0.04 to 0.10]). | Qualitized: The single day workshop improved the confidence of the PTs to perform a number of examination and management techniques re neck disorders, regardless of the extra follow up or not.   1.4 1.7 1.11 |  |  |  |  |  |  |
| Cimoli 2012 | Single Encounter Course | The final survey was formatted as a paper-based survey and consisted of 19 questions to examine the areas of interest: • Current EBP activity (5 items) • Perceived EBP skills (4 items)  • Recognition of EBP terminology and search strategies (3 items) • Beliefs and attitudes to EBP (4 items) • Local barriers to EBP (2 items) • Year of graduation as a physiotherapist (1 item) Refer to Appendix A for a complete version of the survey. Most survey items included four and five response options (4 and 5-point Likert scales). Two questions concerning local barriers   This study consisted of two rounds of data collection. Participants were asked to complete the survey prior to attending the workshop (pre-workshop survey data), and again six weeks later after attending the EBP workshop (post-workshop survey data). | Education Research |  |  |  |  | Prior to attending the workshop, all respondents reported searching either monthly or six monthly. After attending the EBP workshop, most participants reported undertaking searches for clinical practice relevant research on either a fortnightly or weekly basis.  Pre-workshop survey data indicated that most participants were reading research literature related to clinical practice on a monthly basis. When participants were surveyed after attending the workshop most reported that they were undertaking this activity fortnightly. An increase was also seen in the number of participants reading research literature on a weekly basis. | Evidence of the PTs engaging in self-directed learning.  3.2 | A positive shift in participants’ self-reported knowledge and skills in EBP was observed with most participants rating their EBP knowledge and skills as “good” in the post workshop survey  Fortnightly, weekly and daily categories were collapsed and revealed an increase in the frequency of using literature and research in clinical decision-making from 54% pre- workshop to 65% after attending the workshop  Following the workshop, participants were undertaking EBP activity including literature searching and reviewing relevant literature directly related to clinical practice on a more frequent basis. | 4.3 |
| Cleland 2009 | Multiple Encounter Course | At each visit, patients provide a condition-specific disability outcome score and a numeric pain rating (0 –10),28 and these values are entered into the database. The Neck Disability Index (NDI)29 is the condition-specific disability scale used for patients with a chief com- plaint of neck pain. Also included are the patient’s age, sex, symptom duration, and date of surgery (if applicable). The number of physical therapy visits, the duration of physical therapy services, typical costs billed for physical therapy, and insurance provider also can be obtained from the database. | Clinical Practice Leadership | therapists who received the ongoing educational intervention in addition to the 2-day CE course achieved significantly greater reductions in disability for their patients than therapists who only attended the 2-day CE course.   Additionally, therapists in the ongoing education group had a higher percentage of patients who achieved the MCIC for disability than therapists in the control group (64.0% versus 53.5%, P.017).   there were no differences between groups in the percentages of patients achieving the MCIC for pain  From the results of the present study, can only infer that the on-going educational program led to improved clinical outcomes. | Qualitized: PTs in the multiple encounter course potentially implementing interventions achieving greater patient outcomes 1.7 1.11 | Therapists in the ongoing education group used fewer visits (mean difference1.5; 95% CI 0.81, 2.3; P.001) over a shorter duration (median 23 days and 28 days, respectively; P.002) at lower costs (median$599.76 and $735.83, respectively; P.001) than therapists in the control group. | PTs post training evidencing the impact in better patient experience re fewer visits, shorter duration and lower costs. 2.3 2.7 |  |  |  |  |
| Constantine 2012 | Masters Level Program | Data were collected by in-depth interviews conducted on the telephone. The relatively unstructured format of this type of interview is commonly used in phenomenological research and is considered particularly suitable as a method for gaining an understanding of experience | Clinical Practice Leadership Education Research | better able to efficiently integrate the information they gained from their first interview with a patient. They described how their approach had changed from a “set-piece” patient interview to a more “strategic” interview focused on eliciting patient information that would aid their clinical reasoning.  more patient-centered interview: “I definitely let the patient give a narrative now rather than systematic questioning”. Improvements in “hands-on” motor skills were consistently described during the interviews in addition to the improved communication skills previously described. However, most of the participants recognized that improvement in handling skills was only one component of their clinical knowledge base, as Richard explained: “The handling, the reasoning, the thinking, the communication came together – at least in my case – it seemed to have worked for me”.  "each treatment that I do is very much different or specific for that patient and I don't have any stan- dard treatments that I would use."  it has changed my clinical practice for the better”.  it also brought with it some additional professional responsibility to be a role model and share “expert” knowledge | 1.4 1.5 1.6 1.7 1.10 1.11 | While this altered relationship was generally experienced as a positive one, it also brought with it some additional professional responsibility to be a role model and share “expert” knowledge. The participants perceived an expectation that they would serve as an educational resource  The participants characterized their practice, after completing the MSc, as speedy and efficient. Working in this way meant achieving better results with patients sooner | 2.2 2.3 2.7 | As Richard explained: “There's also a pressure because people then start to look to you for peer support or education or being very – errr, testing you. That's what I felt, almost testing you in a nice way, testing you continually”.  Involvement in this type of learning required that the participants engage in a frequently difficult process of deconstructing their own clinical practice followed by a process of reconstruction. This was experienced and framed by the participants as personal development. | 3.1 3.2 3.8 | Also included in the theme of precision thinking were the participants' descriptions of their enhanced ability to access and critically appraise the research literature relevant to manual therapy. These newly developed abilities were closely associated with an increased skepticism about the views of “opinion leaders” or “gurus” in the profession. The participants now felt able to question the lack, or nature, of the evidence supporting the views of manual therapy opinion leaders. | 4.2 4.3 |
| Cowell 2019 | Multiple Encounter Course | Semistructured face‐to‐face qualitative interviews were conducted to explore each physiotherapist's perceptions and experiences of the training programme and its perceived impact on clinical practice. All interviews were audio‐recorded. The topic guide (see Appendix 1) was developed in advance by the research team to elicit in‐depth information about physiotherapists' experiences of the training programme. The interviews were guided by a flexible question route (Silverman, 2010). Minor modifications were made following a pilot interview. One author (I.C.) conducted the one‐to‐one interviews, which lasted between 50 min and 60 min, and took place in the PT's practice setting. I.C. undertook 3 days of training in qualitative research methods prior to commencing the study. He was an experienced male physiotherapist, working in private practice, with a clinical and research interest in BPS management of LBP. He was also a CFT educator. The lead researcher (I.C.) adopted a reflexive stance to the data (Silverman, 2010), acknowledging that the researcher's position may have had an influence on the findings and interpretation of the data. | Clinical Practice Leadership  Education | observing themselves examining and treating patients, in order to develop their communication skills, were highlighted. This helped with developing an appreciation of the interactional consequences of questioning style and the importance of judicious use of language with patients.  A key area for reported self‐confidence was in this aspect of patient communication, in that the physiotherapists were more prepared to ask those difficult questions. "Rather than me being scared about asking the questions, which, again, is probably a change from before to after training, just in terms of confidence."(P09)  "Communication with the patients has changed quite a bit from before the training to after, and you have to be able to communicate well and listen well for this concept to work effectively. The biggest shift for me, personally, is that change in communication, which just allows me then, probably, to identify more drivers." (P09)  This “new” style of communication felt more patient focused and accommodated greater opportunities for the patients to voice their agenda:"And having a fluid conversation with the patient, I think, is very different from a normal physiotherapy assessment, where you have your boxes that you have to tick. I think that would be huge for me. I guess both (i.e. A fluid conversation and physiotherapy assessment forms) of those really sum up for me that you put things on the patient's agenda, and not the therapist's agenda." (P02)  physiotherapists appreciated that a strong patient–therapist alliance was central to the CFT approach, which was facilitated by effective communication: "That first communication session is when you really find out what's going on, and you start to build that relationship with the patient. And if you don't do it in that session, then you might not have that opportunity again." (P08)  helped with developing an appreciation of the interactional consequences of questioning style and the importance of judicious use of language with patients: "Get some feedback on your communication style ... if we pause it here, this is the question you asked; why did you ask that question? ... Or if you'd have said this, do you think that that could have meant that the patient might have reflected on their problem in a different way? And the words you use." (P02) | 1.4 1.5 1.6 1.7 1.11 | In the NHS [National Health Service], I don't have the time. but what I try to do is, I try to make the time, somehow. I'll double‐book patients, so that they have an extra length of time. (P06)  Confidence in identifying psychosocial factors was emphasized, with recognition that this was an extension of their traditional scope of practice: "I'm looking at more of the psychological domains quite a bit more, feel a bit more confident in terms of that." (P 07)  required that physiotherapists extend their traditional scope of practice beyond just physical factors: "I don't think it's an easy approach to use. It is complex because there's so much involved in it and within it but, it's certainly the way forward, for most physiotherapy." (P09)  There was a strong sense that patient outcomes were superior, in that patients often responded more quickly to CFT interventions and the results were more likely to be long lasting: "I would have thought, I'm not sure if I can get you better, whereas now, I think more often, I'm confident in saying, I think I can change this person. I feel much less likely to fail these people now." (P09) | 2.5 2.7 2.11 | The opportunity to analyse and self‐reflect on what happened during real clinical encounters allowed the physiotherapists to gain awareness of all aspects of their own patient–professional interactions  perception that the formal training was the start of a learning journey with a concept that was constantly evolving  Enhancing patients' confidence to move was a catalyst for them to engage in active self‐management strategies: Far more success in terms of a good outcome, and very often a really good outcome, so there's way more than these people that I see using this concept that end up at a point where they can completely self‐manage, most of the fear or the anxiety around the disorders has gone. (P09) | 3.2 3.3 3.8 |  |  |
| Cunningham 2017 | Residency and Fellowship | The assessment tool utilized was based on the American Board of Physical Therapy Specialties Dimensions of Specialty Practice in Orthopedics. The assessment tool was comprised of 58 items within four categories of performance: examination, evaluation, diagnosis, and prognosis. Overall pass rates were analyzed | Clinical Practice | The average live-patient practical examination score for PTs without residency training was 38.2%, and their pass rate was 0.0%. The average live-patient practical examination score for residency-trained PTs was 83.4%, and their pass rate was 92.3%. These findings are statistically significant (p < 0.001). Utilizing a live-patient examination allowed for the assessment of performance in addition to competence (knowledge). These results demonstrate the effectiveness of the training on knowledge, skill development, and most importantly, the clinical reasoning associated with expert practice.  The residency graduates were able to more effectively collect necessary key information and collectively interpret this information to determine a definitive diagnosis. | Qualitized: Residency trained PTs performed significantly better on APTA approved performance exam that looked at ability to clinically examine, assess, diagnose and determine prognosis. Demonstrating an ability to effectively coolect key info and interpret to aid in accurate diagnosis.  1.4 1.5 1.6 1.7 1.11 |  |  |  |  |  |  |
| Cunningham 2019 | Residency and Fellowship | to assess the observable components of clinical reasoning used in the live patient examination, a clinical performance evaluation tool based on the Description of Specialty Practice (DSP) in Orthopaedic Physical Therapy was utilized [10]. The tool assesses the physical therapist’s ability to collect key information, integrate the information into a previous knowledge framework to develop a diagnosis and prognosis, and select appropriate interventions based on this assessment  The assessment form contains a total of 64 items or skills. The tool was chosen for this study because it incorporates context or environment as well as interaction with the patient through a live patient examination  immediately following the final practical examination, residents participated in individual, one-on-one interviews onsite with the primary investigator. The primary investigator used open- ended questions to probe the perspectives of partici- pants and guide the interviews. The participants were asked to share their hypothetical physical therapy diagnosis for the patient and describe the process they utilized to determine the diagnosis. In addition, participants were questioned about their proposed treatment plan based on the diagnosis. | Clinical Practice | Participants demonstrated a significant improvement on the live patient examination from baseline to completion on 17 of the items, in the categories of evaluation, diagnosis, and prognosis. One item in the category of intervention regarding joint mobilization demonstrated a statistically significant change. Two additional items, exercises for mobility and soft tissue mobilization, approached significance at .002.  The categories of examination and diagnosis demonstrated a statistically significant change. The category of prognosis approached significance at .015. The category of evaluation did not demonstrate a statistically significant change.  Residents discussed using the hypothetical-deductive reasoning process and narrative reasoning process throughout the examination of the patient, and in some instances, they integrated both processes to develop a physical therapy diagnosis. Residents also discussed the need to perform a thorough individualized examination and the utilization of key findings to form a hypothetical diagnosis.  "My reasoning has changed in terms of how I listen to the patient tell me her story. Because I listen, I’m trying to come up with either a hypothesis or the nature what this is, or time and pattern of the patient’s pain. So it helped me be able to listen better and include a number of things the patient tells me, so that I’m able to involve the patient also."  Residents discussed the need to examine the patient from a systemic perspective versus looking at the area of somatic symptoms in isolation. They noted the need to recognize both medical and psychological issues present and considered this as a component of the clinical reasoning process.  Through clinical reasoning you are able to determine whether a patient is appropriate for physical therapy. We are able now to tell the red flags or the yellow flags. These kind of things, the psychosocial need. | 1.4 1.5 1.6 1.7 1.8 1.11 |  |  |  |  |  |  |
| Cunningham 2021 | Residency and Fellowship | Semi Structured Interviews Interviews were conducted in September and October 2016. The primary investigator (PI) traveled to each participant’s place of work to perform the individual, face-to-face interviews. The PI had completed graduate training in qualitative research methods and had been mentored by an experienced qualitative researcher in interviewing techniques. Graduates were familiar with the PI as an individual collecting data regarding the outcomes of the program and also from participation in two previous studies. Other than that, there was no relationship with the researcher. Prior to participating in audiotaped, semi-structured interviews; each participant provided informed consent and completed a brief demographic questionnaire. Interviews with both the graduates and employers lasted  approximately 40 minutes each. 1. Could you describe your experience in the residency program? 2. Has your clinical practice changed since the completion of the program?  3. Has completion of the residency program affected patient outcomes? Employers 4. How many residency graduates are practicing in the clinic? 5. Have you noticed a change in their clinical practice? Closure: Is there anything else you would like to discuss before we end this interview? | Clinical Practice Leadership Education | Themes discovered through the interviews: (1) clinical reasoning development, (2) increased efficacy in patient care, (3) consultations for complex patients, (4) enhanced clinic reputation. Each of the themes built upon each other to result in a transformation of clinical practice  Graduates noted the personal change in their practice as the result of their clinical reasoning development. As stated by one graduate, “Clinical reasoning is the best way to do your examination, determine a diagnosis, your prognosis. Then at the end of the day, you know this patient has this problem and you are going to give a correct intervention.” The ability to determine a hypothetical diagnosis was further supported by another graduate: "prior to residency, I was not going deeper to really establish the cause of pain, but now I have the ability to do that. I can confidently do it and distinguish between hypotheses and come up with the right one. I also positively identify the functional limitations and impairments that the patients have."  integrated the knowledge and examination techniques taught throughout the residency. | 1.6 1.7 | the graduates began to provide mentoring to their peers. Not only did the physical therapists notice a change in practice, the patient progress was also recognized in the medical community. recognition as expert clinicians, resulted in increased referrals to the clinic, enhanced clinic reputation, and improved clinic income  “We get more referrals. Now the doctors want to know whether we are doing OMT. So they are sending patients for manual therapy.” This was reinforced by an employer, “We have also experienced some increase in referrals. So that in itself has also created a positive image of the hospital and in particular the department.”  “I am seeing patients for a shorter period and getting tremendous results.” Another graduate noted that this increased efficiency was resulting in financial savings for the patient as well.  transformation in practice was recognized by the residency graduates and their employers alike | 2.3 2.5 2.7 | one-on-one mentoring to their colleagues. As stated by one graduate, “It has reached the point where most of my colleagues come to me for advice on what to do for the patients, which is something they were not doing before.” This was reinforced by another graduate, “When they are seeing a difficult patient, they call me in to see the patient together.   enthusiasm to share knowledge   encouragement of colleagues to complete professional development activities through the invitation to share new knowledge became a motivator to pursue future learning opportunities. | 3.4 3.7 3.8 |  |  |
| Cunningham 2022 | Residency and Fellowship | All residents voluntarily completed a survey to determine the impact of residency education on the professional development and career advancement of residents in the United States. Included demographic information as well as additional nineteen items related to the residents’ professional development and career advancement. The survey utilized a five-point Likert scale ranging from major positive to major negative. The survey was piloted in 2014 with the first cohort of the residency program.   To determine the value and influence of the residency on professional development and career advancement, median values were determined for each of the survey questions.  In addition to the survey, all residents participated in individual, one-on-one, semi-structured interviews conducted at the residency site upon completion of the program. The investigator used open-ended questions to probe the participants and guide the interviews. The same interview guide was used with all participants. A qualitative phenomenology research design was used to describe how the residency was experienced from the residents’ perspective. 20 to 30-min period. | Clinical Practice Leadership Education Research | graduates reported a positive impact of residency education on the ability to 1) perform a comprehensive evaluation; 2) utilize clinical reasoning in treatment decisions; 3) provide an effective treatment to achieve projected outcomes; 4) treat complex patients  acquired clinical reasoning skills to assess the patient and inform an individualized treatment plan  themes emerged from the interviews: - evolution of practice from protocol-driven to individualized treatment plans - positive change in physical therapy practice  With wider treatment perspectives, these residents relied on newly acquired clinical reasoning skills to assess the patient and inform an individualized treatment plan. O.N. commented: "It changes how we would treat things. Like I said initially, two people may have the same problem in the shoulder, but presenting very differently. So you not have the same treatment plan for each and every other patient. You have learned to individualize each person, his or her problem."  performing consultations for difficult patients. D.M. noted: "Then when you go to clinic, you find that your friends and doctors would say, ‘I want you to see my patient.’ Because he knows, after he touches my patient, it is not the same as my colleague touching the patient ... Now colleagues come to you and ask what do you do and you tell them this (is) what I do and I got this knowledge here."  change in practice and the communication of the new approach to patient care was also discussed: "I was talking to my colleagues, not only physios but doctors, and I was telling them what I learned in clinical reasoning; the ICF, the manual skills, everything. How everything is good. It is increased, not only when in physio condition but in general health care conditions. You find that in most conditions, you often have to involve everyone." | 1.2 1.3 1.4 1.5 1.6 1.7 1.9 1.11 | their perspective on the scope of physical therapy was expanded by the residency program  theme from interviews:  - promotion of professional development within the wider physical therapy community  expressed how the program impacted changes within physical therapy in Kenya as a profession "We’ve learned a lot and we do appreciate and it has changed the face of physiotherapy practice in Kenya for many of us"  residents described gaining respect from their colleagues and members of the health-care team fostered by providing them continuing education and consultations for complex patients. This reflects a perceived change in the role of the physical therapist within the health-care team. The residency education in Kenya led not only to a recognition of the graduates’ newly acquired clinical practice expertise but also an opportunity to expand collaborative practice within the wider health-care team. | 2.1 2.3 2.5 2.8 | theme from interviews:  - Commitment to lifelong learning described a commitment to pursuing additional opportunities to further their education.  many residents reported providing information to other members of the health-care team.   the residents discussed promoting a new approach to patient care by providing formal instruction and education to their colleagues, as well as serving as a consultant to their peers for challenging case  - promotion of professional development within the wider physical therapy community | 3.1 3.2 3.4 3.7 3.8 | graduates reported a positive impact of residency education on the ability to 7) implement a treatment plan based on scientific literature | 4.3 |
| Demmelmaier 2012 | Multiple Encounter Course | Number of assessed yellow and red flags  Division of consultation time  Knowledge about red and yellow flags in back pain was measured with three items, modified from Overmeer, Boersma, Main, and Linton (2009)  Biomedical and biopsychosocial attitudes toward back pain was measured with the Pain Attitudes and Beliefs Scale for Physiotherapists (PABS-PT) (Houben et al, 2005a).   The participants’ self-efficacy for assessment of the specific yellow and red flags was measured with a study-specific measure constructed by the first and the last author. It consisted of 11 items corresponding to the 11 factors defined in the main outcome measure ‘Number of screened yellow and red flags.’  Assessment of prognostic factors and time spent on assessment were measured continuously in all phases of the study. Knowledge, attitudes, and self-efficacy were measured at the end of baseline, in the middle of the knowledge acquisition phase, three times during the skills training phase, and immediately after finishing the intervention. | Clinical Practice | Assessment of red flags increased from baseline (1, 0, 1, respectively) to post-intervention (4, 4, 3, respectively) for three participants. One participant remained unchanged and assessed red flags in two consultations both at baseline and post-intervention. They all reported high self-efficacy (range 8–10) for assessing red flags.  Knowledge about yellow and red flags in back pain was substantial at baseline in all participants and increased slightly during the study. Biomedical orientation decreased and biopsychosocial orientation increased to some extent.  Changes over time were seen in all four participants, with increased assessment of prognostic factors and less time spent on detailed discussions about pain symptoms. | Qualitized: PTs improved in the time they took with patients and their diligance in assessing risk and prognosis re yellow and red flags.  1.4 1.8 |  |  |  |  |  |  |
| Dennis 1987 | Accredited Area of Practice Training | Observation | Clinical Practice | The manipulative therapists devoted significantly more time to patient assessment than did the generalists  Statistically significant differences were observed in patient assessment (amount and amount of pain focus) and in treatment selection (passive joint mobilising) and a trend was observed for the use of supervised exercise.  The manipulative therapists spent more time on pain related assessment: 60.78'10 for females and 53.43% for males compared to 46.57'10 and 38.37% for the female and male generalists. The main effect for group based difference was significant (F = 9.0, p < ·01). | Qualitized: The MTs spent more time with patients, focused to understanding their pain experience and tended to use more manual therapy and exercise in their RX choices.   1.4 1.5 1.7 1.11 |  |  |  |  |  |  |
| Deutscher 2014 | Single Encounter Course | Data were collected within the Maccabi health care system from April 2006 to December 2012. Maccabi performs routine outcomes data collection as part of its normal treatment procedure.  Functional status was measured using FOTO’s lumbar-specific computerized adaptive test (LCAT).  final data set included only episodes of care for patients who completed the LCAT both at admission and discharge from therapy. As each episode of care was analyzed separately, the term patient refers to each patient episode of care | Clinical Practice | Physical therapists with more advanced McKenzie educational training were admitting patients with significantly lower FS at admission and had higher (10%-21%) FS change scores compared to physical therapists who had less training. The unadjusted number of visits per episode of care was lower for patients treated by physical therapists with any McKenzie education compared to those with no training, and up to 11% to 13% lower for those treated by physical therapists with a McKenzie education level of Part C and above.  After controlling for patient risk factors and treatment-related confounders, all educational levels were significantly associated with an additional 0.7 to 1.3 FS points at discharge compared to no McKenzie education, with no significant differences between educational levels. The random factor (physical therapist) was significant and explained only 2.2% of the variance in FS at discharge.  After controlling for these risk factors, all McKenzie educational levels were significantly associated with fewer (0.6-0.9) visits compared with no McKenzie education. No significant differences in adjusted number of visits were identified between educational levels, with the lowest coefficient (–0.94) found at the credential level. The random factor (physical therapist) was significant and explained 8.7% of the variance in number of physical therapy visits. | Qualitized: PTs with McKenzie training see more complex patients, help them achieve significantly greater functional scores in less visits compared to those without the education.   1.6 1.7 1.11 |  |  |  |  |  |  |
| Dizon 2014 | Single Encounter Course | The Adapted Fresno Test (AFT) was used to measure changes in EBP knowledge and skills. - The AFT is composed of clinical scenarios relevant to physical therapy and occupational therapy and items related to formulating a question, identifying and searching for sources of evidence, identifying the best study type to answer the question and relevance, validity and significance of the evidence found. The AFT is scored using a scoring rubric and the total possible score is 156.  EBP Attitudes questions related to changing practice if good quality evidence exists, support to undertake EBP activities, and confidence in searching the literature and undertaking critical appraisal. The questions are to be answered by choosing whether the physical therapists agree, disagree or neither.  EBP behavior Behavior regarding EBP was measured by activity diaries. The physical therapists in both groups were instructed to log their activities by placing a tick (√) in the options related to activities used to find the answer to a case they were faced with. | Research |  |  |  |  |  |  | Improvements in EBP knowledge and skills were found in the EBP group compared with the waitlist control group at immediate post-training and three months after the training, as shown in Table 2 and Figure 2 (using the median scores and 95% confidence interval for the median). The improvement in knowledge and skills of the EBP group was significant in all items of the AFT in over all time periods except for Q2 (Sources of information; advantages and disadvantages) at post training **Attitudes:** No statistical difference was found between the EBP group and waitlist control group at pre-training for all but two of the EBP attitudes. This was regarding changing practice if good quality evidence exists, and confidence in undertaking a literature search. Whilst a slightly larger percentage of the EBP group agreed that practice should change if good quality evidence exists, none of the physical therapists (in either group) disagreed. Whilst a larger percentage of the control group agreed they lack confidence in undertaking a literature search, a larger percentage of the EBP group neither agreed nor disagreed, which implies that most of the physical therapists (in either group) were not really confident to do a literature search (Table 4). At immediate post-training and three months post-training, significant differences were found between the groups in terms of attitudes, specifically in terms of the following EBP activities: 1. Lacking confidence in undertaking a literature search (more physical therapists disagreed to this in the EBP group than the control group) and 2. Confidence in undertaking a critical appraisal (more physical therapists agreed to this in the EBP group than the control group). Interestingly, it could be noted that there was also an increase in number in these attitudes items in the control group at post training period. Possible reasons are explored in the discussion section. **EBP behavior**: A total of 37 physical therapists submitted their diaries for analysis (EBP group =18, waitlist control group =19). Of the 18 diaries from the EBP group, 16 had reported cases (unique cases = 14, usual cases = 9) and 2 did not contain any documented activity. Of the 19 diaries from the waitlist control group, 13 had reported cases (unique cases = 10, usual cases = 10) and 6 did not contain any documented activity. For the purpose of reporting, the activities were categorized as to EBP behaviors (formulating PICO, logging PICO, searching research evidence,appraising evidence and applying the evidence) and non-EBP behaviors (asking colleagues, asking medical doctors and reading textbooks). a. Unique cases Fourteen physical therapists from the EBP group and ten from the waitlist control group reported to have had unique cases. For unique cases seen, more physical therapists in the EBP group significantly performed EBP behaviors (such as formulating their PICO, logging a PICO, searching for research evidence, appraising and applying the evidence) compared with the waitlist control group. More physical therapists in the waitlist control group significantly performed non-EBP behaviors (such as asking medical doctors and reading textbooks). No difference was noted between groups in terms of asking colleagues (Table 5). b. Usual cases: Nine physical therapists from the EBP group and ten from the waitlist control group reported to have had usual cases. For usual cases seen, more physical therapists in the EBP group significantly performed EBP behaviors (such as formulating their PICO, searching for research evidence, appraising and applying the evidence) compared with the waitlist control group. No difference was noted between groups in terms of the non-EBP behaviors (asking colleagues, asking MDs and reading textbooks (Table 6) | PTs that underwent the training were mor confident in performing a literature search and critically appraising the evidence to support their clinical practice decisions.  4.2 4.3 |
| Fary 2015 | Multiple Encounter Course | change in physiotherapists’ self-reported confidence in managing people with RA and retention of change  An online questionnaire using Qualtrics software captured outcomes, including the physiotherapist’s self-reported 1) confidence across a broad range of RA knowledge and skills areas (9 and 8 questions, respectively, 5-point Likert scale, range 1–5); 2) satisfaction with the physiotherapist’s ability to manage patients with RA (numeric rating scale 1–10); 3) responses to 6 clinically related statements focusing on red flags and extraarticular features of RA (5-point Likert scale, range 1–5) based on previously identified pertinent clinical issues also identified as important by an expert Delphi panel; and responses to questions relating to 2 clinical vignettes | Clinical Practice | Significant between-group differences were observed at the end of the 4-week RCT. The magnitude of change in outcomes scores in the intervention group was significantly greater than the control group for self-reported measures of confidence in knowledge (between group mean difference [MD] 8.51, 95% confidence interval [95% CI] 6.29, 10.73), confidence in skills (MD 7.26, 95% CI 5.1, 9.43), satisfaction in ability to manage people with RA (MD 2.06, 95% CI 1.42, 2.70), responses to the pre-diagnosis (MD 0.76, 95% CI 0.37, 1.15), and early stage RA (MD 1.49, 95% CI 0.88, 2.10) clinical vignettes (Table 3). Effect sizes for these differences ranged from 0.50–1.62, representing moderate to large effects.  participants who improved their scores in the 6 clinically related statements was consistently higher in the intervention group (20–48%), compared to the control group (6– 21%) (Table 4). | Qualitized: PTs that engaged in the con ed had signifcantly greater confidence intheir knowledge, skills and overall ability to manage patiets with RA.  1.11 |  |  |  |  |  |  |
| Furze 2019 | Residency and Fellowship | As part of their residency education, the residents were required to write reflective narratives across 4 time placements during their one-year residency.  Following completion of the clinical narrative by each individual resident, investigators collected this data for analysis. After the residents submitted their narrative for analysis, all mentors (2–4 depending upon the institu- tion) evaluated each resident narrative individually, providing feedback and questions for the resident to consider. These narratives with mentors’ comments were then used for group discussion with residents and faculty. This group interaction allowed for peer/member checking as well as a second level of analysis and reflection by the resident. This second level of reflection was not included in the data set of this study | Clinical Practice Leadership or Management | The data indicate that residents transitioned from singular to collaborative reasoning that is patient focused. In addition, as residents became more patient focused, they required less validation from peers and mentors about their abilities and professional roles, while being more sensitive to boundaries of caring and better adept to dealing with uncertainty in practice.  contextual decision making allowed them to try to make the best possible decision for an individual patient’s situation even when it might not be their ideal decision.   The residents’ focus gradually changed from singular reasoning that was self-focused to a collaborative reasoning process that focused on the values, needs, and goals of the patient  Eventually, the residents began to find their own internal sense of validation, which helped them to develop self- confidences in their clinical knowledge skills.  Ultimately, some of the residents started to become more comfortable with the uncertainty of clinical practice  the residents’ narratives revealed a gradual development of their ability to access knowledge in their specialty area.  The clinical narratives revealed evidence that residents were beginning to use pattern recognition | 1.2 1.4 1.5 1.6 1.11 | They recognized that their role may expand beyond regular patient care in the clinic to advocacy in a broader context. Components of this theme included: 1) mindful practice; and 2) boundaries of caring.  I immediately recognized that this patient needs some-one from the medical team to step up and be an advocate for them as they kept getting passed along and lost in the shuffle. (O5, narr 4)  I will learn from this situation the importance of education and advocacy for patients that may not have a strong voice in their own care. (O4, narr 4) | 2.10 2.11 |  |  |  |  |
| Green 2008 | Masters Level Program | The two-part study was questionnaire based and allowed for information and views of a large number of participants to be collected. The first section of the questionnaire asked about the participant’s current role and career pathway in chronological order since their initial physiotherapy qualification. The year of graduation from the Post- graduate Diploma/MSc was also identified. The second section explored Master’s education and its influence on their career. This section included: open- ended questions on how the MSc contributed to their current roles and about the people or factors that influenced or hindered the development of Master’s education, key aspects of their role that had changed because of the Master’s qualification and career aspirations.  The questionnaire was followed and complemented by the use of group interviews utilising focus group technique, whereby the largely factual findings from the questionnaire were explored in greater depth with the participants. | Clinical Practice Leadership Education Research | Improved confidence in self and confidence in practice (24) Updating clinical/manual skills (20) Improving clinical reasoning (15) Greater depth of knowledge (13) Development of their own clinical reasoning and the ability to facilitate the clinical reasoning of others (12) Advanced clinical skills (11) Enhanced professional profile (9)  Participants indicated that enhancement of self- confidence was a major contribution to their professional and personal development as a result of studying for this Master’s degree.  Other factors that were cited by participants in the current study were their enhanced knowledge and ability to engage in evidence based practice, enhanced clinical reasoning and their ability to evaluate research. | 1.4 1.6 1.7 1.10 1.11 | Enhanced professional profile (9) Eligible for current role (23)  The Consultant Therapists from this programme represented 14% of all musculoskeletal Consultant Therapists currently in post in England and Wales (Limb, 2005). The remainder were currently engaged in a range of new roles of ESP (14.5%)  Participants agreed that it was the status of the qualification that made them eligible for their new roles. | 2.5 2.7 2.11 | Being able to teach others (12) Involvement in teaching (14)  18.75% have pursued an academic career. | 3.2 3.7 3.8 | Ability to evaluate research and apply evidence based practice (12) Ability to undertake research (9)  Other factors that were cited by participants in the current study were their enhanced knowledge and ability to engage in evidence based practice, enhanced clinical reasoning and their ability to evaluate research. | 4.1 4.2 4.3 |
| Hansell 2023 | Single Encounter Course | The survey was designed to address the domains of user confidence, attaining competence, challenges and/or facilitators faced by course participants, strategies used by trainees to implement, and clinical use of LUS. The survey methodology and questionnaire were based on a previous survey of doctors by Rajamani et al. To test for clarity and ambiguity, the first draft questionnaire was given to three physiotherapists without LUS expertise and feedback was sought to maximise clarity of questions. The second draft questionnaire was administered to three respiratory and intensive care physiotherapists with LUS knowledge, who had attended the course previously, for validation and reliability. Since no changes were made to the survey after this, their responses were included in the final analysis. The survey (Appendix 1) was divided into six domains: demographics, qualifications, employment, course participation and implementation, LUS imaging competence and confidence, and LUS supervision, containing up to 21 questions. For the purposes of this study, we defined competence as having performed 40 supervised LUS sessions | Clinical Practice | LUS experience and attaining competence in LUS. Most physiotherapists surveyed did not achieve competence in LUS and therefore had not obtained accreditation through a governing body (38/39, 97.4%). There was no statistical significance in the relationship between years of clinical experience and gaining competence in LUS, accredited through a governing body (p ¼ 0.777). Only one participant (2.6%) reported achieving competence and thus accreditation in LUS through ASUM  Use of LUS in clinical practice: For the 20 of 39 physiotherapists whom had performed at least one scan following completion of coursework training, three physiotherapists reported performing greater than or equal to 40 scans, two reported completing between 10 and 40 scans, and 15 reported completing 10 scans (Table 3). In response to how often the physiotherapists surveyed performed LUS and interpreted images to inform clinical decision-making, most (29/39, 74%) responded with never. Some used it less than once a month (8/39, 21%), and a small number used it at least once a week (2/39, 5.1%) (Table 3). The physiotherapist who attained accreditation used LUS at least once a week to inform clinical decision-making and was supervised by doctors and fellow physiotherapists | 1.4 |  |  |  |  |  |  |
| Harrison 2022 | Multiple Encounter Course | The primary outcome was the Assessing Competency in Evidence-based Medicine scale (Ilic et al., 2014) to quantify knowledge and skills in evidence-based practice. The secondary outcome was the BARRIERS scale (Funk, Champagne, Wiese, and Tornquist, 1991; Kajermo et al., 2010) to quantify barriers and facilitators to implementation of evidence-based practice. Baseline data were used to quantify knowledge, skills and barriers to evidence- based practice (Aims 1 and 2). Change scores were used to evaluate the impact of the training program (Aim 3).Version B of the BARRIERS scale was used to quantify barriers and facilitators to implementing  evidence-based practice (Funk, Champagne, Wiese, and Tornquist, 1991), with the term “nurse” replaced  by “physical therapist.” It consists of 29 items which are rated on a 5-point Likert scale (1 = to no extent; 2 = to a little extent; 3 = to a moderate extent; 4 = to a great extent; and 5 = no opinion). Scoring of these 29 Likert-scale items is divided into four subscales: 1) Research (i.e. characteristics of how the research is conducted); 2)Healthcare Provider (i.e. characteristics of the physical therapist using the research); 3) Setting (i.e. characteristics of the organization in which the research will be used); and 4) Presentation (i.e. characteristics of how the research is communicated). The items loading into each subscale are listed in Appendix Table B1. The total score for each sub-scale was computed by calculating the average score for all responses in the subscale for each participant, with a missing value assigned when over half of the items are rated as ‘no opinion’ (Funk, Champagne, Wiese, and Tornquist, 1991; University of North Carolina Digital Commons Project, 2020). Scores range from 1 (not a barrier) to 4 (barrier to a great extent). There are also three open-ended questions (other things you think are barriers to research utilization, the three greatest barriers, and the things you think facilitate research utilization). Participants could identify up to four other barriers to research utilization and, if they nominated additional barriers, ranked them on the same 5-point Likert scale used for items 1 to 29. Participants nominated what they perceived to be the greatest barrier, second greatest barrier, and third greatest barrier. Responses to these open-ended questions on additional barriers and facilitators were reviewed independently by two authors to identify themes. | Clinical Practice |  |  |  |  |  |  | The Assessing Competency in Evidence-based Medicine scale total score increased by an average of 0.1 points  with training (95% confidence interval −0.2 to 0.5) (Table 2). This change was not clinically important or statistically significant. The post hoc subgroup analysis indicated that university training in evidence-based practice did not influence this result (Appendix Table D1). The pattern of correct responses after training was similar to the baseline data (Appendix Table A1). All of the BARRIERS subscale scores reduced after training (Table 2). The largest reduction occurred in the Healthcare Provider (mean difference −0.2, 95% confidence interval −0.3 to −0.1) and the smallest reduction occurred in the Setting (mean difference −0.1, 95% confidence interval −0.2 to 0.0). While these changes were statistically significant, it is difficult to interpret their clinical importance. The post hoc calculation of effect sizes ranged from −0.21 to −0.48 (Appendix Table D1) indicating small to medium effects. The post hoc sub-group analysis indicated that all of the BARRIERS sub-scale scores reduced with training for participants who had received evidence-based practice training during their entry-level or post-graduate qualification. However, among participants who had not received this university training, only the Healthcare Provider subscale score was reduced (Appendix Table D1). | PTs that underwent the training improved their score on the competency in evidence based medicine score, which attempts to quantify knowledge and skills in evidence-based practice.   4.3 |
| Heneghan 2022 | Mentorship | 1. Patient reported outcome measures (PROM) were collected: - Musculoskeletal Health Questionnaire (MSK-HQ) (Hill JC et al., 2016) - Patient Specific Functional Scale (Horn et al., 2012; Nicholas and H.C Tumilty, 2012) - Patient Enablement Instrument (PEI) (Howie JG et al., 1998)  - Consultation and Relational Empathy (CARE) measure (Mercer SW et al., 2004).  2. Mentees participated in a semi structured interview. The topic guide (Supplementary file 3) was informed by existing evidence and the core constructs of MSc level practice in MSK physiotherapy. (Rushton A, 2010). | Clinical Practice Leadership Education Research | mentees gained considerable confidence using clinical reasoning skills in practice  “… I think in fact I’ll be more confident this time because I know I’ve built up my clinical reasoning skills, and I can rely majorly on them …” Mentee 5  development of specific skills commensurate to advanced professional practice.  valued opportunties for reflection (subtheme: focus on use of reflective practice) Focus on use of reflective practice “… Now I am very present all the time, my thought analysis, like my weaknesses and bias I am trying to confront them and improve my weaknesses …. ” Mentee 1  Communication skills development:  “… But for now its helping to improve our communication skills and reasoning. You need to be very aware of everything the patient is telling you because everything could be important. I think after COVID our assessment and treatment is going to change in that sense …. ” Mentee 9  Quantitative results: - For pts seen on a 2nd occasion (n=52) the change in MSK-HQ score exceeded the MCID of 6, with a change in the median score of 11.  - Days physically active increased by a single point. - The median scores for PSFS 1 (n =48) and 2 (n =41) reduced by 4, >MCID of 2.7. - Both the CARE (n=47) and PEI (n =46) scores remained high across visits with a ceiling effect noted  From analysis of the CARE instrument free text responses, 2 main themes emerged, ‘experiences of telehealth MSK physiotherapy’ and ‘challenges with telehealth’. For ‘experiences of telehealth’ there were subthemes (surprisingly positive and effective experience, communication skills important, patient empowerment and self-management, therapeutic relationship and patient preference for telehealth).  Author conclusions: Unequivocally, the opportunities to develop and demonstrate advanced communication skills were appreciated by all participants. | Qualitized: meaningful change in health outcomes for patients that were followed up by PTs engaging in mentorship as demonstrated by multiple validated outcome measures.  1.2 1.3 1.4 1.5 1.6 1.7 1.11 | “ …. Before, I wasn’t very good at receiving feedback before my placement, now that’s changed, I’m open to receiving different opinions.… I felt confident to give feedback in a constructive way. They felt confident to tell me when I was being a bit rude and frank. I learned to say things in a better way and be more constructive …” Mentee 9 | 2.4 2.6 | a shift to patient empowerment (subtheme: focus on self-management).  the patient data where they stated a preference for telehealth, valued the focus on self-management leading to empowerment and further evidenced by improved MSK outcomes. | 3.3 3.8 | focus on evidence-based practice, (subtheme: consistency in applying evidence-based practice). Consistency in applying evidence based practice “… It’s changed a lot. It was a weakness. I’ve focused a lot on that. Trying to make it a constant practice. Share evidence and discuss. Before I had a tendency to look at what I want to know – confirmation bias, but now I’m trying to include everything …” Mentee 3 | 4.3 |
| Jones 2008 | Residency and Fellowship | A Web-based survey instrument was developed to gather data from the respondents regarding their professional development and leadership activities, as well as salary and wage information. This format was selected because it was the belief of the authors that the ease of documenting responses with a Web-based survey would facilitate a  high response rate.  The survey instrument had inquiries regarding: year of graduation (residency or entry-level), postgraduation educational advancements, board certification specialty designations, current patient-care activity, teaching experience/pursuits, professional speaking invitations, professional committee participation, manuscript contributions to the professional literature, and compensation levels. Survey questions utilized a closed-ended response format (ie, yes/no, multiple choice, multiple selection). | Education |  |  |  |  | Residency graduates participated in significantly greater numbers of postgraduate fellowship programs (P = .001), attained more board certifications in a physical therapy specialty (P < .001) spent a greater number of years as a primary clinical instructor of a physical therapist intern (P = .007). They also participated (significantly more frequently than non-graduates) as a guest lecturer/lab assistant in a professional or postprofessional PT education program (P = .01), a head instructor in a professional or postprofessional PT education program (P = .05), and a clinical faculty member in a PT residency or PT fellowship program (P = .003) (Table).  Comparisons between orthopedic PT residency graduates and orthopedic non-residency graduates demonstrate statistically significant differences between the 2 groups, with a tendency for the residency-trained group to become more involved in physical therapist education at the academic/university level and clinical residency/fellowship level.  Additionally, residency graduates demonstrate a greater frequency of ongoing pursuit of advanced clinical training by participating in a postprofessional fellowship program and of successfully achieving board certification in some area of physical therapy specialty | Qualitized: PTs with residency training tend to continue to pursure continuing education and tend to take on academic roles re teaching etc.   3.1 3.2 3.8 |  |  |
| Kafri 2023 | Single Encounter Course | 1) Physical therapists’ perceptions of Motor Learning (PTP-ML) questionnaire. ML-related self-efficacy and implementation were assessed by using the PTs’ perceptions with the PTP-ML questionnaire. - self-report questionnaire encompasses three subscales. The “ML self-efficacy” subscale (12 items) measures respondents’ confidence in their knowledge and ability to explain ML principles or terms. The “implementation” subscale (12 items) measures self-reported implementation of ML principles, and the “general perceptions and work environment” subscale (4 items) measures environmental factors that may support knowledge application - items are rated on a 5-point Likert-type scale ranging from “strongly agree” to “strongly disagree” in the “self-efficacy” subscale or from “very little” to “very much” in the “implementation” and “general perceptions and work environment” subscales. The “implementation” sub-scale contains an additional option to check “unaware of this ML principle” to indicate that the respondent was unaware of the concept in question.  - Higher scores indicate higher ML self-efficacy and implementation and the presence of more enablers of ML implementation in the workplace.  2) post-intervention feedback: consisted of nine statements concerning satisfaction with the intervention, changes in practice, and the usability of the intervention. Items were rated on a 5-point Likert scale. the post-intervention feedback also included five, open-ended questions about the application experience  3) follow-up feedback assessments: included nine statements concerning long-term changes in participants’ clinical practices and the perceived educational value of the educational methods | Clinical Practice | Total questionnaire score: Pre-post comparisons of the total score showed a significant increase in participants’ mean score following training: F(1,109)=289, p < .0001, effect size (η2)= 0.57. The mean group RCI was significant, with 69.4% of participants showing an increase in their total score. There were no group (i.e. setting) or interactions effects.  Pre–post comparison of the self-efficacy subscale score showed a significant increase in participants’ mean score following training: F(1,109) = 435, p < .0001, effect size η2 = 0.66. With 80% of participants showing an increase in self-efficacy. There were no group (i.e. setting) or interactions effects.  There were no significant differences in questionnaire subscales and total scores between the post-intervention and follow-up measurements. indicating that the change in scores between pre-and post-intervention was maintained in the long term. There were no setting differences or interactions  Participants felt that the intervention helped them to organize their knowledge in a structured manner. This in turn, allowed them to gain a better understanding of the new knowledge in an appropriate context and to link old and new knowledge.  b. Adding outcome measures. “I am currently planning more of the outcome measures that I will use to assess the patients’ learning process.“ (participant 329). “The model required me to focus on a specific goal and to measure my progress.” (participant 36). | Qualitized: after the education, PTs showed an incread in their confidence in their knowledge and ability to explain ML principles or terms and their self-reported implementation.  1.4 1.7 1.11 |  |  |  |  |  |  |
| Karas 2016 | Single Encounter Course | The survey assessed how often they utilized thoracic spine manipulation (one item), how comfortable they were using it (one item) and their familiarity with the peer-reviewed evidence citing the use of thoracic spine manipulation for neck pain (one item). We utilized a Likert scale that ranged from 1, which represented ‘never’, ‘not at all comfortable’ or ‘not at all familiar’, to 5, which represented ‘very often’, ‘very comfortable’ or ‘very familiar’. Finally, a series of content questions were asked regarding the evidence in the current literature (seven items)  Chart review: Before beginning the KT programme, retrospective chart reviews of six months’ duration were independently completed by a single reviewer to assess each individual PTs’ documented use of MT to the thoracic spine | Clinical Practice | Pre-test/Post-test The descriptive statistics for the pre-test and post-test items are presented in Table 1. The mode utilization score before the education was split between a three, ‘sometimes’, and a five, ‘very often’. However, after the education, the first mode was eliminated and the PTs most commonly reported that they used thoracic ‘very often’. Correspondingly, the mean utilization score increased by 0.31, from 3.77 before the education, to 4.08. The standard deviation dropped by 0.38, indicating that people’s responses were more uniform after the education. Respondents’ mode comfort level and mode familiarity level before the education were both very high (mode = 5 and mode = 4, respectively), indicating that most of these PTs were comfortable with their skills in manipulating the thoracic spine and familiar with the peer-reviewed evidence citing the use of thoracic spine manipulation for neck pain. After the education, the mode comfort level did not change and the mean increased slightly, by 0.15. By contrast, after the education, the mode familiarity score dropped to a three, ‘familiar’, instead of the ‘very familiar’ response that was most commonly selected before the training, and the mean also decreased by 0.15. Both standard deviations also decreased, indicating that people answered these items more uniformly after the education. Wilcoxon signed rank tests were completed on each of these three items and revealed no statistically significant differences between PTs’ responses before and after the treatment, (p = 0.28; 0.68; 0.53, respectively). The percentages of each response for these three items are shown in Figures 1–3. The PTs’ level of comfort in performing thoracic spine manipulation improved slightly after our intervention, from a mean of 3.62 to 3.77, which was not significant. The mode was 5, both before and after the educational intervention, indicating that the PTs felt extremely comfortable with their ability to manipulate the thoracic spine. Self-reports of the PTs’ utilization of thoracic spine manipulation increased from a mean of 3.77 to a mean of 4.08. The two most common responses before the intervention were 3 (‘sometimes’) and 5 (‘very often’). However, after the intervention, the mode was 5 (‘very often’), indicating an increased use of the techniques following the KT intervention. Those who may not have utilized thoracic spine manipulation prior to the KT intervention seemed to have increased their use, leading to a mode of 5 (‘very often’) (see Figure 1)  Treatment applications per patient The percentage of times that the PTs applied MT (mobilization and/or manipulation) was calculated from the total number of patients with neck pain, and corresponding ICD-9 codes, which each PT treated during the six-month time frames before and after the KT intervention. The use of thoracic spine mobilizations (grades 1–4) increased after the KT intervention. Before the PTs received the KT intervention, they performed mobilizations on 19.2% of their patients, whereas after the intervention they performed them on 42.3% of their patients (see Figure 4). The use of thoracic manipulation or grade 5 mobilization followed a similar pattern, although not as extreme. Before the intervention, the PTs performed manipulations on 9.9% of their patients with neck pain, whereas afterwards this increased to 14.8% of their patients (see Figure 5). Likewise, the percentages of patients who received both mobilization and manipulation increased from 7.5% to 15.7% (see Figure 6). The percentage of patients who did not receive either treatment decreased from 65.0% before the training to only 27.5% after the training | Qualitized: after the education, PTs in general increased how often they utilized thoracic spine manipulation, how comfortable they were using it and their familiarity with the peer-review evidence citing the use of thoracic spine manipulation for neck pain.   1.6 1.7 1.11 |  |  |  |  |  |  |
| Karvonen 2015 | Multiple Encounter Course | The study material was collected at a single Finnish health-care center and included the documentation for the treatment of 57 patients, which was written by six PTs, who had completed the continuing education training. Two experienced physiotherapy experts also analyzed those documents. They were blinded to the original PTs’ decisions. he work experience of the two reviewers, as both PTs and tutors, was about 40 years each. In addition, they each had 3 years of specialized PT training in the field of orthopedic manual physiotherapy (OMT). One of the PTs had a master’s degree in health care and the other had a doctoral degree in health sciences. | Research | The PTs and reviewer 1 agreed on 77% of all cases, with a K coefficient of 0.69 (Table 2). The PTs and reviewer 2 agreed on 72% of all cases, with a K coefficient of 0.64 (Table 3). The expert reviewers mutually agreed on 74% of the cases, with a K coefficient of 0.63 (Table 4). Comparing the two reviewers with the six PTs, the overall agreement was 74%, and the K coefficient was 0.63   our results are important and suggest that short, but intensive, continuing education training can transfer knowledge regarding LBP classification to PTs at a moderately good level.  the results suggest that PTs participating in this study benefitted from the continuing education training, in terms of clinical reasoning and diagnostic decision-making processes. Evidence of their enhanced preparation was seen in the quality of the patients’ clinical examination, in clinical reasoning and in diagnostic conclusions. | Qualitized: when compared to 'expert' PTs, those PTs who had undergone the education demonstrated good agreement with theis patient Assessments, suggesting that the education benefited the PTs in terms of clincial reasoning, ax and dx.   1.4 1.5 1.6 1.7 1.11 |  |  |  |  |  |  |
| Kerssens 1999 | Multiple Encounter Course | The physiotherapists recorded the instructions given to their patients in each session by means of a registration form.  Patients were asked to answer the same questionnaire on three separate occasions. These questionnaires contained, among others, questions about instructions, given by therapists, and their perceived effectiveness, possible discussion of (non)adherence, and solutions offered by therapists. All questions had a precoded format. | Clinical Practice | In both the pre-training and post-training phase, most instructions were spent on back care in daily activities and exercises. About 15% concerned pain management and 8% general fitness.  In the post-training phase more patients told their physiotherapists about their problems in the early stage of treatment (67% at T1). However, the differences are not statistically significant.  In a large majority of cases the physiotherapists were able to help their patients. As this was already the case before the training, the differ- ences between the measurement moments and pre-training or post-training phase are small and not statistically significant.  After the training the physiotherapists who participated in our study spread their patient education and instruction more equally across the different sessions in which they treated their patients. There were fewer instructions, avoiding instructional overload. The last session was utilized better after the training to recap the main points of instruction. | 1.4 1.5 1.11 |  |  |  |  |  |  |
| Lambrinos 2023 | Single Encounter Course | 2 separate visual analog scale answers about their confidence with MI-E: one for prescription and one for application of therapy on a scale from 0 (not confident at all) to 10 (extremely confident). The primary end point was the change from the baseline to the post-intervention confidence on the visual analog scale questions for both the prescription and application of MI-E between the intervention and control groups  Competency was assessed by using 10 multiple-choice questions that covered the key components of MI-E fundamentals. One point was awarded for each correct answer, with a total score of 10. | Clinical Practice | There was a greater improvement in the intervention group for the primary end point with self-reported confidence for prescription of MI-E improving by mean 3.6 (95% CI 4.5 to 2.7) more than the control group, with a final mean 6 SD visual analog scale score of 6 6 1.5. Likewise, confidence in the application of MI-E improved by mean 2.9 (95% CI 3.9 to 1.9) more in the intervention group than in the control group, to a final mean 6 SD vis- ual analog scale score of 6.9 6 1.6. There was also a significant difference between the groups in the secondary end point, the theoretical measure of MI-E competence, with a greater improvement in the aggregate multiple-choice questions score of mean 3.2 (95% CI 4.3 to 2) in the intervention group compared with the control group, to a final mean 6 SD score of 7.9 6 1.8 of 10 (Table 2). | Qualitized: PTs who underwent the MI-E training imrpoved their self confidence in prescription and application of the therapy. They also demonstrated significant improvement in their theoretical competence on assessment.   1.7 1.11 |  |  |  |  |  |  |
| Lane 2022 | Single Encounter Course | Baseline data were collected before a patient’s initial PT session. Data were collected at baseline, 2 weeks and 12 weeks remotely by Research Electronic Data Capture. At baseline, patients supplied demographic information including age, sex, race/ethnicity, employment status, general medical information and current history of symptoms, and fear avoidance beliefs measured with the Fear Avoidance Beliefs Questionnaire.  The primary outcome was the Patient-Reported Outcomes Measurement Information System (PROMIS) Physical Function (PF)-computer adaptive test (CAT) at 12 weeks. The PF-CAT assesses an individual’s capability to perform physical tasks and produces a T score with higher numbers indicating greater function. The scale that has excellent reliability and validity is highly responsive to change and able to detect a 1.2% difference at 80% power. .  Secondary outcomes included the PROMIS pain interference CAT, Numerical Pain Rating Scale assessing pain intensity, Pain Self-Efficacy Questionnaire (PSEQ) assessing patients’ confidence in their ability to do daily activities despite pain, Treatment Self-Regulation Questionnaire to measure the degree of autonomous motivation to follow  a treatment regimen and engage in healthy behaviors, Pain Catastrophizing Scale assessing negative pain cognitions, Working Alliance Theory of Change Inventory to measure therapeutic alliance between a patient and PT, and the NPQ to measure knowledge of pain physiology. In addition, all patients were asked at the 2-week assessment, “Did your therapist discuss your pain with you as an indicator of a perceived threat rather than an indicator of damage or injury to the tissues?” This question was meant to serve as a measure of fidelity that one of the key messages of PNE was delivered to the patient.  2.6. Physical therapist self-report measures At the time of training, general demographic and practice information were collected, including years of experience, advanced degrees or training, etc. We also collected the NPQ and Health Care Provider’s Pain and Impact Relationship Scale (HC-PAIRS) to assess PT’s attitudes and beliefs about pain. The HC-PAIRS measures healthcare providers’ beliefs about the relationship of a patient’s pain and their physical impairment | Clinical Practice | [Results from unadjusted models are shown in Table 3. For the primary outcome, we did not observe differences in PF-CAT by the treatment group at 12 weeks (mean difference = 1.05 [95% confidence interval [CI]: −0.73 to 2.83], P = 0.25) or at 2 weeks (PF-CAT mean difference = 0.29 [95% CI: −0.77 to 1.34], P = 0.59).  Evaluation of secondary outcome measures did not find any between-group differences with the exception of the PSEQ. At both 12 and 2 weeks, patients in the PNE group reported higher PSEQ scores than patients in the UC group (mean difference = 3.65 [95% CI: 0.00-7.29], P = 0.049 and = 3.08 [95% CI: 0.07 to −6.09], P = 0.045, respectively). Results of fully adjusted models were comparable with the unadjusted results (Appendix Table 2, available as supplemental digital content at  At 2 weeks, 108 of 254 overall patients (42.5%) responded “yes” to the fidelity measure. In the PNE group, 48 of 108 patients (44.4%) responded “yes,” whereas in the UC group, 60 of 146 patients (41.1%) responded “yes.”  We also used explored the potential influence of the number of visits received on our main outcome between groups. The mean number of physical therapy visits attended by enrolled patients was 10.0 visits (SD = 6.9, range 0-39). Patients in the UC group had a mean = 9.0  (SD = 6.0, range 1-37) visits. Patients in the PNE group had a mean = 11.4 (SD = 7.8, range 0-39) (mean difference 2.4,95% CI: 0.8-4.1, P = 0.004) visits. To explore if the number of visits may have impacted change in PF-CAT scores, the number of treatment sessions by  the percent change in PF-CAT (from baseline to 12 weeks) is graphed in Appendix Figure In both treatment groups, we did not observe an association of the number of treatment sessions with changes in PF-CAT scores (P = 0.94 for PNE, P = 0.79 for UC)](http://links.lww.com/PAIN/B458) | Qualitized: patients treated by PTs that had undergone the PNE course demonstrated improved Pain Self-Efficacy i.e patients’ confidence in their ability to do daily activities despite pain.   1.5 1.11 |  |  |  |  |  |  |
| Lawford 2018 | Multiple Encounter Course | [Semi-structured interviews. Interviews were conducted in the weeks prior to the first training day and repeated after the final training day. In accordance with a constructivist paradigm, interview topics were designed to explore physical therapists’ beliefs about their role managing patients with OA, as well as their perceptions about their training experiences including a description of their pre- and post-training beliefs and practices. For convenience and to facilitate participation in the research, interviews were conducted over the telephone. Interviews were conducted by the same investigator (BJL), a graduate research student trained in qualitative methodologies, who is not a clinician, and who also attended the training program with the physical therapists, but was otherwise unknown to them. Interviews were audio-recorded and transcribed verbatim, and pseudonyms assigned to each physical therapist for confidentiality purposes. Each interview lasted approximately 30 minutes. All data were de-identified and stored in digital format on a password-protected university server.](http://onlinelibrary/) | Clinical Practice | Post-training. Theme 1: defining person-centered care. After training, therapists did not change their description of person-centered care, but instead acknowledged a deeper and more complex understanding of it (Table 5). Importantly, therapists described an increased ability to integrate person-centered care within their patient consultations. Theme 2: sharing responsibilities. After training, therapists believed that they had a bigger role to play in supporting their patients to adhere to prescribed exercise and physical activity recommendations. This belief was particularly apparent in relation to creating strategies or plans to help patients move forward with their exercise program and assisting them to make a decision to take action and commence an exercise program. Therapists noted they should be less prescriptive with patients in clinical practice. Theme 3: changed conceptions of role. There was evidence of both acceptance, and resistance, to incorporating person-centered care methodology into usual clinical practices after training. Therapists believed that the training had positively impacted their communication style with patients in the clinic, with many spending more time discussing personal barriers and facilitators to exercise. Some acknowledged that it was difficult to change their practice habits, and George did not believe that a person-centered approach suited his personality or the way he liked to interact with patients, and did not intend to incorporate it into his practice. | 1.4 1.5 1.11 |  |  |  |  |  |  |
| Lawford 2019 | Single Encounter Course | Following training, each physiotherapist was randomly assigned four patients with knee OA, in order to practice implementing the person‐centred practice principles and techniques taught.  Using the audio recordings, physiotherapists were asked to self‐audit 50% (to minimize physiotherapist burden prior to starting the main trial) of their consultations (including any two initial and two follow‐up consultations), resulting in four audits each.  In addition, the training facilitator (C.B.) indepen-dently audited all telephone consultations using an identical audit form. Fidelity to person‐centred practice principles (scored from 0 to 10 as described above) was classified as “low” if scores were between 0 and 4, “moderate” if between 5 and 7, or “high” if between 8 and 10 | Clinical Practice | 3.1 \| Primary aim: Use of person‐centred practice principles Table 2 reports audit findings against the 10 person‐centred practice principles. Average physiotherapist audit scores ranged from a mean (SD) of 5.5 (1.7) (“use the wait ʼtil 8 technique to allow people time to think and respond to questions”) to 6.9 (1.2) (“using a person‐centred approach that promotes a person's choice and control”) out of 10. The average training facilitator audit scores ranged from 6.2 (1.2) (“address all four aspects of goal setting when discussing and setting goals with a person”) to 7.0 (1.4) (“using a person‐centred approach that promotes a person's choice and control”). As such, the physiotherapists and training facilitator alike believed that the therapists showed “moderate” fidelity to person‐centred principles, with mean scores for all 10 principles lying between 5 and 7 (out of 10)  3.2 \| Primary aim: Use of essential behaviour change techniques Figure 2 depicts the proportion of physiotherapists who were using each technique effectively, according to both self‐audit (n = 32 calls) and audit by the training facilitator (n = 61 calls). Both self‐ratings and training facilitator ratings indicated that physiotherapists were using three of seven (43%) techniques “effectively” during the majority (≥50%) of consultations, including: (a) tracking and monitoring strategies; (b) RICk (Readiness, Importance, Confidence, knowledge) radar (intuition); and (c) client first. Physiotherapists needed to “improve their skill level” using the techniques of changing thinking habits and menu of options in more than 50% of consultations. The technique of RICk‐focused decisional balance was rated as “not applicable” by the training facilitator in >75% of consultations | Qualitized: PTs that underwent the training were demonstrating person sentres principles with moderate fidelity and effectively using 3/7 techniques taught relating to behaviour change.   1.5 1.11 |  |  |  |  |  |  |
| Levsen 2001 | Multiple Encounter Course | The patients with Chronic Low Back Pain completed the Revised Disability Pain Questionnaire, also designated as the Oswestry; the patients with Chronic Shoulder Pain completed the Shoulder Rating Questionnaire. These two functional tools have been reported in the literature to be valid and reliable | Clinical Practice | The two-sample t-test also demonstrated therapists in Group 1 used significantly fewer visits than therapists in Group 2 (df=38, t=-4.06, p<0.001) as shown in Figure 2. Therapists in Group 1 also had a significantly larger efficiency ratio (df=38,t=4.82, p<0.001) compared to therapists in Group 2, which is shown in Figure 3.  improvement between initial vs. discharge scores displayed by patients in Group 1 (difference of means=26.2) was almost twice that of Group 2 (difference of means=13.4).  The ANOVA and post-hoc test results for the Chronic Low Back Pain sample supported our hypothesis that therapist training had a positive effect on functional tool scores. For this sample, patients treated by Group 1 therapists had a significantly greater improvement in functional tool scores and used significantly fewer visits than patients treated by Group 2 therapists. | Quantized: Patients treated by PTs who participated in the education utilized fewer visits and had better patient outcome scores at discharge compared to those treated by PTs without the education.  1.11 |  |  |  |  |  |  |
| Lonsdale 2017 | Single Encounter Course | We conducted participant assessments at baseline, 1 week, 4 weeks, 12 weeks, and 24 weeks after each participant’s first physiotherapy appointment. Patients self-reported their overall adherence to their physiotherapists’ recommendations by using 7- point rating scales. They also reported the proportion of specific rehabilitation exercise they completed during the previous week (ie, sessions completed/sessions prescribed) and their leisure time physical activity (ie, sessions completed/sessions prescribed). Physiotherapists rated patients’ in-clinic adherence by using 5-point rating scales | Clinical Practice | Overall, CONNECT training for physiotherapists had a weak positive effect on patients’ self-reported home-based adherence (dZ.28; PZ.01), with significant effects found at week 1 (dZ.32; P<.01), week 4 (dZ.30; P<.01), and week 12 (dZ.27; PZ.03). These differences were not maintained at week 24 (dZ.25; PZ.14), but effect sizes at week 12 and week 24 were not statistically different (P>.05). The CONNECT intervention had no significant effect on physiotherapists’ ratings of in-clinic adherence or on the proportion of specific back exercises that participants reported completing at home. There was also no significant effect on physical activity. CONNECT did not have a significant effect on any of the clinical outcomes (eg, pain, function, and satisfaction with treatment) or quality of life. CONNECT training had a moderately significant positive effect on patients’ perceptions of competence to follow their physiotherapists’ recommendations (dZ.66; P<.01). This effect was not observed immediately after the treatment (dZ.36; PZ.16), but was found at week 4, week 12, and week 24 (dZ.56 to dZ.97; P<.01). The CONNECT intervention also had a significant overall positive effect on patients’ amotivation (dZ .42; PZ.01). Once again, this effect was not observed immediately after the treatment (dZ .25; PZ.19), but was found at week 4, week 12, and week 24 (dZ .37 to dZ .59; P<.01). The effects of the CONNECT intervention on autonomous motivation were not observed, perhaps because of ceiling effects (ie, patients reported high scores at baseline on a 7-point scale; mean score, 6.6400.58 in the experimental arm and 6.6000.54 in the control arm). CONNECT training for physiotherapists also did not influence controlled motivation (PZ.71) or fear-avoidance beliefs (PZ.36). Similarly, patients’ ratings of their physiotherapists’ need-supportive behavior were not influenced by the CONNECT intervention, because both arms had scores that were near the scale maximum of 7 immediately after their first treatment session (mean score, 6.7000.68 in the experimental arm and 6.5500.77 in the control arm). | Qualitized: The educational intervention improved physiotherapists capacity to influence patient's adherence to their HEP, patient's perception of competence and also their motivation to participate in active therapy.   1.5 1.7 1.11 |  |  |  |  |  |  |
| Louw 2022 | Single Encounter Course | 1) change in pain at rest (NPRS); 2) change in pain with activity(NPRS) ; 3) change in disability; and, 4) residual disability (NDI and ODI)  2) The following 6 outcomes were analyzed for each of the completed patient cases before and after the PNE training program: 1) number of total treatment visits; 2) total duration of care in days; 3) number of total units billed for the entire case; 4) average number of units billed per visit; 5) percentage of “active” billing units relative to total units billed and 6) percentage of “active and manual” billing units relative to total billing units. “Active” billing units were considered therapeutic exercise (CPT code 97110), therapeutic activity (CPT code 97530), neuromuscular reeducation (CPT code 97112), and gait training (CPT code 97116). “Active and Manual” billing units were considered any active billing units as above plus man- ual therapy (CPT code 97140). | Clinical Practice | Post hoc analyses, with a Bonferroni correct alpha of .025 (comparison of pre- and post- for both regions) revealed that patients with LBP had greater improvement in disability scores with therapists after the PNE course than those treated by the same therapists before the PNE course (p = .004)  Post hoc analyses, with a Bonferroni correct alpha of .025 (comparison of pre- and post- for both regions) revealed that patients with LBP had greater residual disability improvement with therapists after the PNE course than those treated by the same therapists before the PNE course   patients with LBP received a higher percentage of active treatment than patients with neck pain regardless of time. Additionally, there was a statistically significant main effect for time (p < .001), indicating that patients received a higher percentage of active treatment after PNE regardless of body region. | Qualitized: PTs implemented effective Rx that resulted improved outcomes relating to best practice management of LBP (active Rx).  1.5 1.7 1.11 | There was not a statistically significant main effect for region (p = .656) but there was for time (p = .017) with fewer visits reported after PNE than before PNE, regardless of body region.  patients with LBP received a higher percentage of active treatment than patients with neck pain regardless of time. Additionally, there was a statistically significant main effect for time (p < .001), indicating that patients received a higher percentage of active treatment after PNE regardless of body region. | Qualitized: PT's enhanced quality and reduced unwarranted variation and in line with best practice in Rx after engaging in the eduation.  2.7 |  |  |  |  |
| Maas 2012 | Multiple Encounter Course | Participants completed an online test based on 4 clinical vignettes 1 week before the start of the program and within 2 weeks after completion of the program. Four clinical vignettes were based on upper extremity disorders in the context of direct physical therapy access.  At pretest, all participants were asked to formulate 3 learning goals. At posttest, they were asked to indicate the extent to which their goals were achieved on a 3-point scale (1not achieved, 2partly achieved, and 3achieved). It contains 3 sub-scales: the engagement with reflection, the need for reflection, and the insights obtained by reflection. Engagement and need refer to the practice of inspecting and evaluating one’s own thoughts, feelings, and behavior; insight refers to understanding them.  At posttest, participants were asked to indicate how much guideline knowledge they had at pretest and how much at posttest on a scale from 1 (no knowledge) to 5 (much knowledge) | Clinical Practice | mean pretest scores on vignettes were comparable between Peer Ax (PA) and Case Discussion(CD) groups. At posttest, the PA and CD groups showed significant improvement: PA groups 29.82 (SD 63.97), P.001, and CD groups 9.49 (SD 40.52), P.001. Percent improvement was 5.8% for the PA groups and 2.0% for the CD groups. Multilevel linear regression analysis, controlling for sex, showed that the difference between the PA and CD groups was statistically significant in favour of the PA groups (estimated effect 22.52 points; 95% CI2.38, 42.66; P.031).  At posttest, participants in the PA groups showed greater awareness of their professional performance. The correlation between perceived improvement and assessed improvement was r.36 (P.002) for the PA groups and r.08 (P.50) for the CD groups. The difference was statistically significant (estimated effect14.73; 95% CI2.78, 26.68; P.01). | Qualitized: patients in the PA group demonstrated improved clinical reasoning and also greater self awareness/ reflection of their capacity.   1.3 1.6 1.11 |  |  | the results related to attainment of personal goals showed that scores were significantly higher for the PA groups than for the CD groups (estimated effect0.50; 95% CI0.04, 0.96; P.03).  Self reflection and insight scal (SRIS) showed no difference between the PA and CD groups. At posttest, scores were significantly improved in both PA and CD groups: PA groups2.34 (SD8.69), P.001, and CD groups1.85 (SD7.05), P.001. Percent improvement was 2.8% for the PA groups and 2.2% for the CD groups. The difference between the PA and CD groups was not statistically significant | Qualitized: PTs in PA group appeared to attacin personal goals moreso than the CD group, as well as improved self-reflection and insight.   3.1 3.2 |  |  |
| MacPherson 2019 | Residency and Fellowship | The general interview questions and topics were predetermined through discussion and agreement among the research team. To clarify and expand participant responses, the interviewers were allowed to follow-up questions on the responses to the initial questions and encourage discussion of unexpected topics initiated by the participants in a semi-structured format using open-ended questions  Study participants were scheduled for a 30-minute web-based interview with one of two authors. Both interviewers hold American Physical Therapy Association Certifications as Orthopedic Specialists, hold academic faculty positions, and have completed fellowship or residency training. One of the interviewers had contributed to the targeted fellowship program in the past but did not receive his training from this program. The other interviewer had no association with the fellowship to enhance trustworthiness and credibility of present study findings. The two interviewers met to discuss and determine interview questions to meet the overall research questions and ensure consistency and pilot tested the questions and interview process with another author prior to conducting the interviews separately. Subjects provided verbal informed consent and answered demographic questions prior to initiating the interview. Interview audio were transcribed verbatim and transcripts were checked by an independent reviewer for accuracy | Clinical Practice Leadership Education | Time management The majority of participants (7 of 13) reported improvements in efficiency of clinical practice. The comments centered around speed, accuracy, and effectiveness of decision making, as well as basic organization both in clinical care and overall practice tasks.  Practice satisfaction Twelve of the thirteen participants indicated higher satisfaction with practice. Examples focused on enhanced passion and a state of greater happiness with their professional practice since going through the fellowship. Often, increased professional satisfaction was attributed to being connected with similar individuals who helped facilitate a positive outlook and also to a feeling that they were contributing to the profession, another theme discussed later in this manuscript. Participants attributed this outcome to improvements in clinical reasoning ability, broader skill sets to help patients, and enhanced confidence, and two other identified themes/subthemes noted to be interrelated.  Active and open listening Several participants (9 of 13) in the present study indicated enhanced communication skills subsequent to training that centered on active listening, or ‘listening to understand’ [13]. This extended beyond just listening to patients to being more open to other viewpoints, being able to reflect on those viewpoints, and accepting feedback both in clinical practice and non-clinical situations.  There were major changes perceived by the majority of subjects in their self-awareness and metacognition, noted by 12 of the 13 subjects. Interestingly, the changes in cognitive processing occurred both in the clinical and non-clinical settings, | 1.2 1.3 1.4 1.5 1.6 1.11 | Active and open listening Several participants (9 of 13) in the present study indicated enhanced communication skills subsequent to training that centered on active listening, or ‘listen- ing to understand’ [13]. This extended beyond just listening to patients to being more open to other viewpoints, being able to reflect on those viewpoints, and accepting feedback both in clinical practice and non-clinical situations.  The acceptance of new professional roles beyond previous daily clinical roles was perceived as positive for 11 of the 13 subjects. Common areas of positive professional evolution were as follows: (1) new roles in professional teaching, both entry-level and post- professionally, (2) new national and/or state association leadership roles, and (3) promotions or perceived beneficial changes in career not specifically associated with daily patient care. | 2.5 2.6 2.7 2.10 2.11 | The acceptance of new professional roles beyond previous daily clinical roles was perceived as positive for 11 of the 13 subjects. Common areas of positive professional evolution were as follows: (1) new roles in professional teaching, both entry-level and post- professionally, (2) new national and/or state associa- tion leadership roles, and (3) promotions or perceived beneficial changes in career not specifically associated with daily patient care. | 3.8 |  |  |
| Madi 2018 | Masters Level Program | Methods of data collection included quantitative measurement of clinical reasoning skills through the SCT and DTI, as well as qualitative documentary analysis, semi-structured interviews, focus groups and overt observations   Script concordance test (SCT) (Charlin et al., 1998) - Weighing the diagnostic significance of a piece of clinical information Diagnostic Thinking Inventory (DTI) (Bordage et al., 1990) - Assess students’ ability to recognize and interpret clinical cues, and the flexibility of moving between different working hypotheses  Semi-Structured Interviews Focus Groups  Overt Observation  • Recognising learners’ biographies and prior experiences augments their motivation to participate in professional learning. • Tailored and personalised programme pedagogy promotes students’ agency. • Personalised learning empowers students to be active contributors to the learning. • Recognising students’ ‘voices’ maximises their readiness for advanced practice roles. • Negotiated learning facilitates the construction of knowledge and skills that are relevant to practice. • The flexibility of programme structure in recognising different fields of interaction supports knowledge integration. | Clinical Practice Leadership Education Research | Changes in Clinical Reasoning Skills: The programme’s culture of convergence and synergy led to the successful advancement of clinical reasoning skills  The evidence from the qualitative and quantitative data indicated that this culture led advancement in multiple domains related to clinical reasoning skills. The data suggest that the advancement was gradual and progressive ateach phase of data collection. From the beginning of the programme to the point of completing all the modules, data indicated an increase of 28.8% and 13% of SCT and DTI structure in memory scores respectively (Table 6.1). The individual student’s scores are presented in Figures 6.2-5. In Figure 6.2, the data from all students, but Simon, demonstrate a steady increase in the total SCT scores through the three phases of data collection. This concurs with the increase in the DTI structure in memory scores between the start and the end of data collection (Figure 6.5). while the mean scores of DTI flexibility in thinking slightly dropped during the programme, scores of individual students demonstrate variable tendencies (Figure 6.4).  Advanced communication skills, “They made me listen to the things the patient is saying that I wasn’t putting an interest in. I wasn’t really listening because I didn’t think that they may have been important” (Simon, Interview3) Personalising management, “You got to spend much more time and effort in your subjective assessment, to try and really understand what is going on with the patient; because every patient is different. And really trying to tailor the specific treatment or advice or education or whatever you’ve chosen to do to fit that patient.” (Victoria, Interview3) Managing complexities, “I consider far more hypotheses now than I would have before. Just thinking about the knees now, I could think of so many potential different diagnoses and ways to test them.” (Danielle, Interview2) Advanced knowledge base, “I started using things like ‘hats’16 I never used before; like anxiety and depression. I am just like: I think there is something going on here, would you mind telling me more about it?” (Charlie, Focus Group1) Evidence from this study identified that the advancement in clinical reasoning skills start as early as the students are able to integrate M-level knowledge into clinical experience. Both qualitative and quantitative data indicate that the advancement of clinical reasoning skills was gradual and progressive throughout the lifespan of the programme. After being qualified for [a number of] years, you are quite confident in your ability; but I think on reflection, some of that is probably misplaced. If I look at what I know now and what I knew then, I thought that I knew quite a lot then, and in fact I knew absolutely nothing. And now, I know a lot more, but in the grand scheme you know even less because there is always more to know. (Ethan, Interview2) I think [the programme] made me look at things a little bit more. I never went and said that I’m a great physio. I know this and that, but I think it made me realise that there is so much to develop. There are different ways of looking at things that maybe I’ve missed [...] I think it changed the way I look at things, the way I read, the way I go about my practice and the way I re-assess. I consider things a little bit more, I keep things quite broad now, for longer time, and I always question myself [...] I am able to start the treatment in the first appointment. I give the patients more specific treatment, and also [I am] being able to explain to them specifically what’s going on. It is definitely beneficial for the patient. They are getting better quicker. (Interview2)  He advocated changes to workplace in-service training, which became more interactive. Due to his sense of empowerment, Charlie engaged more in collegial knowledge exchanges and challenges, which indicated a movement into the centre of the workplace environment. | 1.1 1.2 1.3 1.4 1.5 1.6 1.7 1.8 1.9 1.10 1.11 | Having a sense of self-efficacy, “I am confident that I am right, that I am able to add a real value. I am able to clinically reason decisions in my own mind which support the decisions that I made with the group. I am able to challenge the things that the group might suggest, and I am confident in doing that. I wouldn’t have been as so when I started the programme.” (Ethan, Interview2)  You grow a thick skin quickly. You don’t take it hard. You don’t get put down by it. You look at it as constructive criticism, rather than being upset with the feedback. You think: right, these are the areas that they [educators and peers] have seen that I need to work on. I am going to go away over the next couple of evenings and [I will] make sure I target those areas. Whether when I am talking to another classmate or reading some research or whether going back to the lecture notes, or ask other tutors. You improve on your errors or weaknesses. (Danielle, Interview3)  By the end of the programme, the students were confident and empowered to engage and in open and collaborative workplace environments  He advocated changes to workplace in-service training, which became more interactive. Due to his sense of empowerment, Charlie engaged more in collegial knowledge exchanges and challenges, which indicated a movement into the centre of the workplace environment.  Category 1: Raising awareness - The category raising awareness describes processes through which students were exposed to a new level of knowledge, thinking and model of practice that supported the advancement of their clinical reasoning skills. These processes started early and continued throughout the lifespan of the programme. Students became aware of the level at which they needed to work and therefore altered the way they approached their practice. As an outcome of raising awareness, the students began to reconcile and develop personal and professional identities. Within this conceptual category, three subcategories are encapsulated, namely: Promoting critical thinking and challenging beliefs, Promoting reflection and introspection, Ongoing feedback | 2.1 2.3 2.4 2.5 2.6 2.7 2.10 | Becoming self-evaluative and reflective, “The master [program] has provided me with the tools and skills to actually become aware of my mistakes and the ways that I can make them better” (Danielle, Interview2) Students also embraced collaborative learning and collective clinical reasoning in university and workplace environments The programme had an impact on Charlie’s professional learning, which indicates a continuity of the programme’s educational message. He changed the way he approached his professional development activities. He was motivated to engage in M-level modules as a source professional development because of the high level of criticality associated with it. He advocated changes to workplace in-service training, which became more interactive. Due to his sense of empowerment, Charlie engaged more in collegial knowledge exchanges and challenges, which indicated a movement into the centre of the workplace environment. We do quite a lot of in-service trainings at work. I slightly changed how we run them. It used to be led by one person. To get more people involved, we would take a topic, so for instance we are doing an [X topic]. Instead of someone leading that, everyone has a particular [area of the topic] to go and research the most relevant or recent information on that, and then present that back to the group. So rather than having one person to do it all, we are actually incorporating everyone. (Interview 3)  Category 2: Collegial knowledge exchange The category collegial knowledge exchange’ constitutes a conceptualisation of the social and interactive nature of the programme’s environment. It relates to the processes through which students share knowledge and externalise tacit knowledge as part of the programme. It suggests that collegial knowledge exchange is a critical component of advancing clinical reasoning skills, not only in terms of offering insights into peers’ experiences but also in facilitating knowledge transition, integration and a comprehensive understanding of patients’ presentations. Therefore, it is interconnected with the category of raising awareness. This category encapsulates three main subcategories, namely: Learning from peers, Collective knowledge construction, Continued learning  Personalising management, “You got to spend much more time and effort in your subjective assessment, to try and really understand what is going on with the patient; because every patient is different. And really trying to tailor the specific treatment or advice or education or whatever you’ve chosen to do to fit that patient.” (Victoria, Interview3) | 3.1 3.2 3.3 3.4 3.5 3.7 | They need to be able to justify it from an evidence-based perspective and to critically justify selection of the management approach within a biopsychosocial framework. (Educator)  We challenge everything. This week, we have been together [in an optional module]. I’d loved if you’ve watched us on a film [and see] how we were in the first week compared to how we are now. Because we just sat there and we were just like: ‘But why? That’s rubbish; that can’t be true.’ We do it to each other. We do it to [named educators] now. I think it shows that we are just a lot more critical in everything that we know. (Ethan, Focus Group2)  I would say that I changed; maybe the structure; I question myself earlier; why I am thinking what I am thinking, have I misdiagnosed, if I misdiagnosed let's re- assess, what is the sensitivity and specificity of the tests? (Interview3)  This facilitated transformative changes in becoming a critical practitioner who is able to justify his practice and drive his own learning.  Supporting evidence-based practice and practice-based evidence during reflection.  We do quite a lot of in-service trainings at work. I slightly changed how we run them. It used to be led by one person. To get more people involved, we would take a topic, so for instance we are doing an [X topic]. Instead of someone leading that, everyone has a particular [area of the topic] to go and research the most relevant or recent information on that, and then present that back to the group. So rather than having one person to do it all, we are actually incorporating everyone. (Interview 3) | 4.1 4.2 4.3 4.4 4.5 4.8 |
| Mansell 2020 | Multiple Encounter Course | Focus groups at the workplace were conducted within 3months of completion of the training programme, lasted approximately 1hour and were audio-recorded  The Association of Chartered Physiotherapists in Respiratory Care Acute Respiratory/On-call Physiotherapy Self-evaluation of Competence (ACPRC) questionnaire was developed to assist physiotherapists undertaking on-call duties to identify their self-perceived competence and confidence, thus facilitating identification of their learning needs.Each item of the ACPRC questionnaire wC50:C54as scored using a range of 0–4 (0 strongly disagree to 4 strongly agree). An ACPRC questionnaire score is calculated and reported as a percentage 0%–100%.19 Additionally, subsections of the ACPRC questionnaire can be calculated: assessment skills, treatment skills, treatment skills matrix, range, managing a call-out and confidence. A lower score indicates less and a higher score indicates more confidence in an on-call scenario | Clinical Practice Education | The median difference in overall ACPRC questionnaire scores pre- Simulation Based Education (SBE) and post-SBE was significant (median difference 5.5%, 95% CI 2% to 16%, p=0.034). The r score (0.57) suggests there was a large effect size  Participants reported their assessment skills were improved through use of the ABCDE approach,26 which they felt reduced the chance of errors or omissions. Participants reported the Situation, Background, Assessment, Recommendation cogntive aid improved their non-technical skills, by enhancing communication with the multidisciplinary team and reducing anxiety surrounding receiving a phone call while on-call.   Participants felt SBE improved both their clinical reasoning skills, and confidence in decision making.  The ability to self-assess, and thus identify learning needs, is essential in safeguarding patient safety by ensuring professionals perform tasks within their scope of practice. The task of self- assessment is a skill which is rarely formally taught | Qualitized: as per the questionnaire results, after the training, PTs reported improvement overall in: assessment skills, treatment skills, range, managing a call-out and confidence.  1.2 1.3 1.4 1.6 1.7 1.9 1.11 | ‘SBE provides coping strategies for dealing with on-call-related stress’. This theme suggests one benefit of SBE is its ability to reduce stress experienced by on-call physiotherapists which is a novel finding of this study. Using SBE techniques appears to have provided coping strategies, resulting in a calmer on-call experience, thus reducing stress.   “It’s those skills you can carry over perhaps not physio specific but dealing with stress” (Participant 3)  The ability to self-assess, and thus identify learning needs, is essential in safeguarding patient safety by ensuring professionals perform tasks within their scope of practice. The task of self- assessment is a skill which is rarely formally taught | 2.3 2.8 | All participants reported SBE assisted them to identify learning needs by facilitating recognition of their weaknesses and areas for improvement.   All participants identified SBE facilitated reflective practice, hence improving the learning experience and their ability to translate learning into changes in their practice.  The ability to self-assess, and thus identify learning needs, is essential in safeguarding patient safety by ensuring professionals perform tasks within their scope of practice. The task of self- assessment is a skill which is rarely formally taught | 3.1 3.2 3.6 |  |  |
| March 2024 | Single Encounter Course | Participant knowledge, confidence, behavioural intention, and simulation experience were assessed using an a priori questionnaire based on our learning outcomes. This survey was informed by Kirkpatrick’s evaluation framework stages one, two and three. These surveys explored agreement with eleven items, with responses based on a seven-point Likert scale. Responses for the knowledge survey explored agreement with each item, with options ranging from 1 =strongly agree, 4 =neither agree nor disagree, 7 =strongly disagree. Responses for confidence explored frequency and ranged from “almost never” (score of 1) to “almost always” (score of 7).  We assessed implementation of skills learnt during the workshop using the Normalization Measure Development (NoMAD) tool at T3-six weeks after the simulation-based workshop (Finch et al., 2015). The NoMAD is a 23-item questionnaire designed for tailoring based on the intervention being tested. We used 20-items in four categories, with responses ranging from ‘strongly agree’ (score of one) to ‘strongly disagree’ (score of five) on a five-point Likert scale. Three response options were also provided if participants considered the item not relevant. | Clinical Practice | data from self-reported confidence in learning outcomes. Baseline data for two outcomes was sufficiently high that the predicted improvement of two levels on our seven-point Likert scale was unachievable. Statistically significant responses were observed on repeated measures ANOVA for all outcomes (p=<0.001 to p =0.02). Statistically significant differences were noted for all outcomes between T1 and T2, and for five of six outcomes between T2 and T3. Responses were on a seven-point Likert scale ranging from “almost never” (score of 1) to “almost always” (score of 7).   A novel, simulation-based educational strategy improved confidence in patient-centred care in musculoskeletal physiotherapists, which was sustained six weeks after the workshop. Participants reported very high knowledge of learning outcomes at baseline, limiting the potential effect of our intervention to increase knowledge. Participants had very high behavioural intention to use the knowledge and skills after the workshop and reported a very positive experience with the simulation-based education workshop. Participant-reported data from six weeks after the workshop demonstrated that participants transferred their skills into the clinical setting, and reported that the intervention had high coherence, high cognitive participation, and high reflexive monitoring, with improvements needed in collective action. | Qualitized: PTs that underwent the SBE demonstrated improvemet in knowledge, confidence and behavioural intention changes re Rx OA based on the results of the questionnaire  1.7 1.11 |  |  |  |  |  |  |
| Murray 2015 | Single Encounter Course | Audio recordings were made of initial treatment sessions involving 24 physiotherapists, each with a different patient (ie, the patient’s first visit to the physiotherapist). Using a computer-based algorithm, an independent researcher randomly assigned audio recordings to the 3 raters. Raters each listened to 12 recordings and used the Health Care Climate Questionnaire (HCCQ) to assess physiotherapists’ needs-supportive communication. Thus, 12 randomly selected recordings were rated by a single rater, whereas a further 12 were double-rated and interrater reliability was assessed. The 6-item HCCQ is designed *to measure the extent to which a health care practitioner interacts with his or her patient in a needs-supportive manner,* and example items included “the physiotherapist listened carefully to how the participant wanted to do things” and “the physiotherapist tried to understand how the participant saw things before suggesting how to do things.” The scale includes 7-point Likert scales, anchored at 1 (not true at all), 4 (somewhat true), and 7 (very true).17 Previous scores derived from the HCCQ have demonstrated good interrater reliability and construct validity. | Clinical Practice | An independent samples t test demonstrated that there was a large between-arm difference in needs-support scores (dZ2.27; 95% confidence intervalZ1.18e 3.21; P<.001), with intervention arm physiotherapists (mean SD, 4.570.85) rated as significantly more supportive than control arm physiotherapists (mean SD, 2.780.72).  Analyses indicated that the intervention had a large positive influence on physiotherapists’ needs-supportive behavior with patients under experimental conditions, thus supporting the main study hypothesis | Qualitized: PTs who did the 8 hrs of communication skills demonstrated 'significantly' more supportive behaviours in their interaction with patients.   1.5 |  |  |  |  |  |  |
| Naidoo 2022 | Residency and Fellowship | Residents selected a patient presenting with region specific complaints and submitted deidentified patient objective data, and followed this prompt ‘Write a description of your clinical reasoning process, as best as you can recall, throughout your management of this patient, including the initial evaluation and 1–2 follow-up visits’. After submitting the first narrative, which served as a baseline, residents were introduced to the CRS model. Residents in this study, the stories of experience included a total of 20 resident narratives subjected to thematic analysis by the researchers. Three researchers established codes related to CRS a priori. These codes were labeled and operationally defined according to the reasoning procedure: diagnostic, narrative, intervention procedures, interactive, collaborative, reasoning about teaching, predictive, and ethical (Table 1). Researchers then independently coded an expert’s narrative to achieve an intercoder agreement. Researchers reached an 85% agreement on coding the expert narrative, which is within the acceptable range of agreement. | Clinical Practice Education | Evidence of diagnostic and intervention procedures reasoning was found in 100% of the narratives analyzed.Residents moved through a systematic process to eliminate red flags and identify the need to refer or seek consultation  Predictive reasoning involves envisioning the future with the patient, providing information which informed whether to proceed with PT, and potential outcomes. Predictive reasoning was present in 25% of the first narratives and increased to 75% in the final narratives  At the end of residency, 100% of residents were using collaborative reasoning. Residents highlighted strategies forminimizing the power differential between a patient and therapist  Analysis of the narratives in this study revealed the evidence of diagnostic reasoning and reasoning about intervention procedure in 100% of resident narratives analyzed. It is unsurprising that this type of reasoning is on the forefront of residents’ minds, given the focus on diagnosis and intervention during residency training | 1.2 1.4 1.6 1.7 1.8 1.11 |  |  | Reasoning about teaching encompasses patient education and assessing whether intended learning occurred. ­Residents reflected on how they leveraged multiple modalities of education to ensure patient understanding |  |  |  |
| Ntoumenopoulos 2017 | Single Encounter Course | At enrolment prior to course commencement, the participants were given an online questionnaire with 10 multiple-choice question.  A post-course questionnaire included 21 multiple-choice questions covering key aspects covered in the one-day course including the DTU findings for normal lungs, pleural pathology (pleural effusion, pneumothorax) and key acute pulmonary pathologies (pulmonary oedema, pneumonia, lung collapse  Half of the participants, who worked at the health institution where the course was conducted, also undertook a practical examination of their DTU skills. The skill test lasted 15 min, and the clinician was assessed on their ability for key thoracic and abdominal organ identification (normal lung/chest, lung aeration (A lines, B lines), pleura, ribs, heart, liver or spleen), image optimisation (depth/gain), calliper measurements, still image/video clip acquisition, image storage and image review utilising a normal male model (physiotherapy students). | Clinical Practice | The assessment scores (mean percentage, SD, 95% CI) increased from a mean of 73.3 15.5% (63.4–83.2) before the training (Figure 3) to 86.3 5.5% (82.8–89.8) after training.  All participants felt that this course would impact on their clinical practice and they felt confident that they would begin to use DTU in their clinical work. | Qualitized: PTs demonstrated improved knowledge and skills in the specific area of diagnostic thoracic ultrasound after attending the one day course based on a written and practical exam.   1.4 1.11 |  |  |  |  |  |  |
| Olsen 2015 | Multiple Encounter Course | The Adapted Fresno test (AFT). The AFT is a seven-item test developed for rehabilitation professionals, educators and researchers to measure change in the EBP skills and knowledge (EBP competence) following training in EBP [36]. The AFT measures EBP knowledge about: information sources, the hierarchy of evidence, the study design that best answers questions about effectiveness, keywords and limits to use when searching and methodological biases in study designs. The AFT measures EBP skills related to: the ability to write a focused clinical question, the ability to reflect upon advantages and disadvantages of information sources, the ability to describe an effective and efficient search strategy and the ability to interpret and critically appraise a published paper. The AFT is focused around different clinical scenarios relevant to rehabilitation professionals. There are three versions of the AFT that include identical items, but different sets of clinical scenarios to help minimize practice effects when AFT is used for pre-, post- and follow-up testing [36]. The total score range from 0–156, and the test takes 20 minutes to complete and 20 minutes to score using a scoring matrix developed by McCluskey and Bishop [36]. The AFT has been reported to have acceptable psychometric properties, with excellent inter-rater reliability for AFT total score (ICC > 0.9) and acceptable internal consistency (Cronbach’s alpha 0.74) [36]. Improvements of 10% (15.6 points) in the mean total score at post-workshop, and 15% (23.4 points) at follow-up are considered as educational- ly important change, when compared to baseline [37]. The AFT is most useful for evaluating change in novice learners [36].  The EBP beliefs scale. The EBP beliefs scale was “. . .designed to measure clinicians’ beliefs about the value of EBP and their beliefs/confidence in implementing it in practice” ([38], p. 209); and Melnyk et al. found that the scale was sensitive to a wide range of attitudes ([38], p. 214). Melnyk et al. ([38], p. 210]) defined EBP beliefs as “. . .endorsement of the premise that EBP improves clinical outcomes and confidence in one’s EBP knowledge/skills”. The test contains 16 statements addressing EBP beliefs on a continuum from 1 (strongly disagree) to 5 (strongly agree) (5-point Likert scale).   The EBP implementation scale. The EBP Implementation Scale is designed to measure clinicians’ implementation of essential components and steps of EBP [38]. Melnyk et al. ([38], p. 210) defines EBP implementation as “engaging in relevant behaviours, including*: (1) seeks and appraises scientific evidence, (2) shares evidence or data with colleagues or patients, (3) collects and evaluates outcome data, and (4) uses evidence to change practice.”* | Clinical Practice Leadership Education | The GEE regression analyses showed statistically significant differences in favor of the intervention for all three outcome measures at post-intervention. At follow-up, the group difference was statistically significant for two of the outcome measures: the AFT (mean difference = 37, 95% CI (15.9–58.1), P <0.001) and the EBP Beliefs scale (mean difference = 8.1, 95% CI (3.1– 13.2), P = 0.002)   At follow-up we found statistically significant between-group differences in favour of the intervention group with regard to EBP knowledge, skills and beliefs, but not for behaviour. When comparing measurement over time, a statistically significant increase in mean scores, with regard to knowledge, skills, beliefs and behaviour was found for the intervention group only.  In our study, we assessed all the typical outcome measures, including EBP behaviour and found statistically significant between-group differences at post-intervention in favour of the intervention group for all outcome measures, but changes related to behaviour were not sustained at six-month follow-up | Qualitzed: PTs that participated in the course demonstrated onboarding of evidence informed practice knowledge, skills, beliefs and behaviours.  1.6 |  |  |  |  | The GEE regression analyses showed statistically significant differences in favor of the intervention for all three outcome measures at post-intervention. At follow-up, the group difference was statistically significant for two of the outcome measures: the AFT (mean difference = 37, 95% CI (15.9–58.1), P <0.001) and the EBP Beliefs scale (mean difference = 8.1, 95% CI (3.1–13.2), P = 0.002) (Table 4) Comparing measurements over time within groups, we found a statistically significant increase in mean scores related to all outcome measures for the intervention group only. The GEE analysis was adjusted for gender and years of experience. Further adjustment for age, type of position, size of position and type of post-graduate education gave only marginal differences. Total observations from the participants included in the GEE analysis from the intervention group were 40 and 37 from the control group (Fig 1) | 4.3 |
| Overmeer 2009 | Multiple Encounter Course | Pain Attitudes and Beliefs Scale for physical therapists The PABS-PT consists of 36 items aimed at measuring the treatment orientation of physical therapists towards the treatment of back pain [14]. Therapists are asked to rate statements about the treatment of back pain on a 6-point Likert scale ranging from ‘totally disagree’ to ‘totally agree’. The PABS-PT consists of two factors, one measuring the biomedical treatment orientation (10 items) and one regarding the biopsychosocial treatment orientation (nine items).  Health Care Providers Pain and Impairment Relationship scale The HC-PAIRS is a questionnaire for assessing the attitudes and beliefs of health care providers in general in terms of functional expectations for patients with back pain. It consists of 15 statements that have to be rated on a 6-point Likert scale ranging from ‘totally disagree’ to ‘totally agree’. A high score on the HC-PAIRS r*eflects a belief in a strong relationship between pain and impairment indicating an attitude that pain justifies disability and limitation of activities.* Houben et al. found HC-PAIRS scores significantly correlated with all work and activity recommendations reported by therapists in the Netherlands, and a regression analysis found the HC-PAIRS to be the strongest predictor of these recommendations.  Patient vignettes: We included three patients vignettes constructed by Rainville [21]. By means of these vignettes we assessed whether patients’ characteristics such as severity of pain symptoms, severity of pathology and physical job demands would affect the course participants’ work and activity recommendations to their patients.  We chose to access the course participants’ knowledge with two separate questions about psychosocial prognostic factors: 1. When psychosocial factors are mentioned in the context of back pain, what factors do you think of?’  2. Select the three most characteristic psychosocial factors from a list of total 16 psychosocial factors, four of which were evidence-based psychosocial risk factors. We used a 10-minute video of the initial assessment of a fictional back patient to evaluate the physical therapists’ skills. The participants were instructed to: ‘Write down what you think is important in what this patient says in this video’ To measure the perceived change in the physical therapists’ behaviour and patient satisfaction we sent a questionnaire to their patients 6 weeks after the start of treatment. | Clinical Practice Research | Responses to the open knowledge question showed a significant increase (c2 = 42.74, P < 0.001) in knowledge of evidence-based psychosocial risk factors.  Physical therapists’ attitudes and beliefs - Before the course the course participants scored a mean of 41.4 (SD 4.8) on the biopsychosocial factor of the PABS-PT. After the course they scored a mean of 43.5 (SD 4.7) indicating a significant increase (Z = -2.06, P < 0.04) in biopsychosocial attitudes and beliefs. The scores on the biomedical factor of the PABS-PT changed from 25.9 (SD 7.6) before the course to 17.8 (SD 6.3) after the course indicating a significant decrease (Z = -5.09, P < 0.001) in biomedical attitudes and beliefs. The scores on the HC-PAIRS changed from 41.8 (SD 6.8) prior to the course to 38.0 (SD 6.3) after the course, indicating a significant decrease (Z = -2.89, P < 0.004) in belief in a strong relationship between pain and impairment and a decrease in the attitude that pain justifies disability and limitation of activities. The scores for the PABS-PT and the HC-PAIRS before and after the course are shown in Table 4. The pre–post scores on the patient vignettes showed a significant decrease (Z = -4.25, P < 0.001) in the stated influence on patient characteristics such as severity of pain symptoms, severity of pathology and physical job demands on the physical therapists' work and activity recommendations  Skills: When we compared before and after the course what the participants had written down from the video with our checklist it showed a significant increase (c2 = 17.52, P < 0.001). Before the course the therapists indicated a total of 684 items of which 291 corresponded with the checklist, accounting for 54% of the 546 identifiable remarks or cues. After the course they indicated a total of 748 items of which 401 corresponded with the checklist, accounting for 73% of the 546 identifiable remarks or cues. Because our instruction to the physical therapists was very general (‘Write down what you think is important in what this patient says’) they wrote down a number of other items they thought were important like pain level, radiation, site of complaint and so on accounting for the large number of items not on the checklist | Qualitized: PT's changed significantly on important process variables like attitudes, beliefs, knowledge and skills after a university course. Their attitudes and beliefs became more biopsychosocially and less biomedically orientated, they were less convinced that pain justifies disability and limitation of activities, and their knowledge and skills on psychosocial risk factors increased.   1.4 1.11 |  |  |  |  |  |  |
| Overmeer 2011 | Multiple Encounter Course | Questionnaires were sent by mail to all patients at the start of treatment and 6 month later. The patient questionnaire included: demographic questions, a question about pain intensity during the preceding week, the Quebec Back Pain Disability Scale: 20-item self-administered scale designed to assess the level of functional disability in people with back pain, the Pain Catastrophizing Scale (PCS): 13-item, self-administered questionnaire in which people are asked to reflect on past painful experiences and indicate the degree to which they experienced thoughts or feelings during the episode of pain, the Hospital Anxiety and Depression (HAD) Scale: 4-item, self-administered questionnaire designed to measure anxiety and depression with 2 sub-scales  Measured physical therapists’ attitudes and beliefs by: Pain Attitudes and Beliefs Scale for Physical Therapists (PABS-PT): 36 items aimed at measuring the attitudes of physical therapists about the treatment of back pain. the Health Care Providers Pain and Impairment Relationship Scale (HC-Pairs): a questionnaire for assessing the general attitudes and beliefs of health care providers in terms of functional expectations for patients with back pain | Clinical Practice Research | All groups of patients both before and after the course showed reductions in pain intensity and disability; however, there were no significant differences in pain (F0.85; df1,225; P.9) or disability (F1.1; df1,222; P.3)   Again, all groups of patients both before and after the course showed reductions in pain intensity and disability; however, there were no significant differences between patients of therapists who had participated in the course about psychosocial factors and patients of therapists who had not participated in such a course  The results are shown in Table 8. For disability, however, the results showed that patients who had higher levels of catastrophizing or higher levels of depression and were treated by therapists whose attitudes had changed toward a more biopsychosocial perspective had better outcomes than such patients who were treated by thera- pists whose attitudes had not changed in that direction  higher levels of depression seemed to show greater reductions in disability if the attitudes and beliefs of their physical therapists changed toward a more biopsychosocial perspective and a less biomedical perspective during the course | Qualitized: PTs who participated in the course appeared to have improved outcomes with patients who had higher levels of catastrophizing and depression.   1.7 1.11 |  |  |  |  |  |  |
| Perry 2011 | Masters Level Program | Focus group was held in an informal meeting room at Coventry University. All participants were informed about the study purpose and the nature of their involvement. Each participant signed a consent form and granted permission for the discussion to be recorded. At the commencement of the session, participants were given time to read the questions and the opportunity to make notes on their thoughts. The facilitator (KH), a co-author and an experienced focus group coordinator, introduced the purpose of the study, and the ground-rules, emphasising that all experiences are valid and legitimate. On completion of the focus group the facilitator summarised the main emergent issues for confirmation and clarification by the group thus aiding understanding and facilitating an initial identiication of tentative themes. Immediately following the focus group, the facilitator (KH) and the co-researcher (JP - a co-academic and an MSc manipulative therapy graduate) debriefed and recorded initial impressions of the group discussions and examined their notes of the proceedings. The tape-recorded narrative data obtained was transcribed and then verified by an independent qualitative research academic (AG) and the transcripts and the notes made by the facilitator (KH) and the co-researcher (JP) became the data for analysis | Clinical Practice Leadership Education Research | In the ‘cognitive domain’ participants described an increase in their confidence, personally at home; with career progression at work, and professionally in communication skills. In the ‘practical’ domain participants expressed enhanced confidence in taking on tasks that they would have previously considered beyond their capabilities. Participants also acknowledged an enhanced ability, and confidence, in interpreting and communicating at a higher level, and enhanced skills in ‘thinking’ and problem solving (musculoskeletally and generically) despite, in some cases, not having all the pieces of the ‘puzzle’. Participant 7 described this within a professional context: “I found that the course gives you confidence to think. You actually enjoy the complicated patient that comes through the door... you think “oh yes” where it used to be “oh no!”.  Within the professional arena participants described significant improvements in their musculoskeletal working practices. This was expressed as an enhancement in their ability to problem-solve complex clinical cases, to focus on multiple issues and to better reason especially where information was either absent or ill-defined: “You tolerate ambiguity much better, both clinically and personally. Clinically, I see quite complicated cases and a lot of information that patients give you is quite ambiguous and the [MSc] programme allows you to wade through the ambiguity to put it in a less ambiguous context. It allowed me to focus on several issues at once.” (P7).  They described how their professional standing within their departments was enhanced with an elevated level of professional acceptance and a respect (often unspoken) for their musculoskeletal clinical skills and opinion.One quote clearly illustrates this phenomenon:“I found that once you have your MSc people have a certain amount of respect for you | 1.2 1.3 1.5 1.6 1.7 1.8 1.10 1.11 | In the ‘cognitive domain’ participants described an increase in their confidence, personally at home; with career progression at work, and professionally in communication skills. In the ‘practical’ domain participants expressed enhanced confidence in taking on tasks that they would have previously considered beyond their capabilities. Participants also acknowledged an enhanced ability, and confidence, in interpreting and communicating at a higher level, and enhanced skills in ‘thinking’ and problem solving (musculoskeletally and generically) despite, in some cases, not having all the pieces of the ‘puzzle’. Participant 7 described this within a professional context: “I found that the course gives you confidence to think. You actually enjoy the complicated patient that comes through the door... you think “oh yes” where it used to be “oh no!”.  They described how their professional standing within their departments was enhanced with an elevated level of professional acceptance and a respect (often unspoken) for their musculoskeletal clinical skills and opinion. For some, this had resulted in grading enhancements. One quote clearly illustrates this phenomenon:“I found that once you have your MSc people have a certain amount of respect for you. Once I told them I had done some MSc modules people’s eyes lit up because they’ve done something similar and I think it’s an acceptable level of knowledge. I can imagine it will open doors and I’ve certainly used it with regard to Agenda For Change banding..”(P3) | 2.7 2.8 2.10 2.11 | This finding has not been reported in other articles but was universally acknowledged by the participants in this study. The participants described, within an academic context, a transient period of disregard for musculoskeletal research and a degree of scepticism and cynicism about learning received on other in-service manual and manipulative therapy training courses. Others perceived this exigent period within a career progression context, as a process of questioning their own and others practice. “I found I became very critical on courses because you go on a course and you re now expecting things to have some evidence base and people are still running courses where theyre using the Guru approach and they just expect you to sit down and take it and they don't like it when you start questioning. I think it's quite frustrating you pay a lot of money to go on a course, and you think this isn't how it should be running at all and that's quite negative.(P4) Others had a more positive experience of management support, claiming that this support had enhanced their career progression and skills further and encouraged actualisation of their abilities beyond the levels achieved by the Masters programme: “My managers have been supportive all the way through the programme. I think it's because she [the manager] has just done her Masters degree and she understands what it takes. You give a lot back as a result, which I think works both ways" (P4) Participants also described actualisation within their personal lives as illustrated by participant 5' s comments: “Doing the programme made me feel more alive. Before I felt like I was just doing work and treading water and my brain had turned into mush, but doing the Master's made me feel kind of stimulated again. I also felt I developed a kind of more reasoned approach to the rest of my life. Others seeing changes in knowledge-base, clinical skills and ability to apply to practice. Ability and confidence to take students & keep up! Using and developing acquired skills; Supporting of others; Confidence; clinically, academically, educationally | 3.1 3.2 3.3 3.7 3.8 | “I found I became very critical on courses. because you go on a course and you’re now expecting things to have some evidence base and people are still running courses where they’re using the Guru approach and they just expect you to sit down and take it and they don’t like it when you start questioning.I think it’s quite frustrating you pay a lot of money to go on a course, and you think ‘this isn’t how it should be running at all’..and that’s quite negative.” (P4)  This finding has not been reported in other articles but was universally acknowledged by the participants in this study. The participants described, within an academic context, a transient period of disregard for musculoskeletal research and a degree of scepticism and cynicism about learning received on other in-service manual and manipulative therapy training courses. Others perceived this exigent period within a career progression context, as a process of questioning their own and others practice. | 4.2 4.3 4.4 4.8 |
| Peter 2013 | Single Encounter Course | evaluation included online questionnaires among PTs participating in the educational courses. All participating PTs were sent a hyperlink to an electronic questionnaire by e-mail before the educational course (T0), immediately afterwards (T1) and 3 months thereafter (T2). The questionnaires consisted of measures of satisfaction with the educational course, knowledge on hip and knee OA and its treatment and self-reported adherence to the guideline. Satisfaction Survey:  all rated on a point scale of 010 (higher score means more satisfaction): (i) How do you rate the content of the educational course? (ii) How do you rate the gained know- ledge? (iii) How do you rate the applicability of the educa- tional course to your daily practice? Knowledge Knowledge was measured using a self-developed know- ledge questionnaire with 19 questions that were directly derived from the guideline. Ten items concerned theoretical knowledge (seven on initial assessment, one on treatment and two on evaluation). The knowledge questionnaire comprised multiple choice and multiple response questions. In the case of a multiple choice question, a correct answer yielded 4 points. In a multiple response question, the score range depended on the number of correct answers: 4 points in the case of the maximum of three correct answers, 2 points in the case of two correct answers and 1 point for one correct answer. This yielded a total score range of 076, with a higher score indicating more knowledge. Adherence The participants were given a questionnaire concerning adherence to the recommendations in the updated KNGF guideline on hip and knee OA: Quality Indicators for Physical Therapy in Hip and Knee Osteoarthritis (QIP-HKOA) [18]. This questionnaire contained 18 process indicators. The 18 items were scored using a 5-point Likert scale: 0 = never; 1 = seldom; 2 = sometimes; 3 = generally; and 4=always. The total score range was 072, with a higher score meaning greater adherence to recommendations. | Clinical Practice | The mean knowledge score increased after the educational course at T1 in both groups, but decreased slightly between T1 and T2 (Table 2 and Fig. 2). Results from the linear mixed model showed a slightly greater change of the knowledge score in the IW group compared with the CE course group, with the difference persisting over time; however, the difference was not statistically significant (P = 0.278).  in both groups the mean adherence score improved between baseline and directly after the educational course at T1, as well as between T1 and T2. Taking into account all time points, a statistically significantly greater improvement of the adherence score over time for the IW group compared with the CE course group was seen (P = 0.024). | Qualitized: both groups of PTs that engaged in a continuing ed course on most recent clinical practice guidelines pertaining to OA, demonstrated increase in knowledge after the course, with the interactive workshop group showing a significant improvement in adherence to the guidlines overtime compared to the conventional group.  1.11 |  |  |  |  |  |  |
| Peter 2015 | Single Encounter Course | The primary outcome was self-reported adherence with the recommendations in the guideline, while knowledge about the contents of the guideline and perceived barriers in using the guideline were the secondary outcomes. The questionnaire to assess the effect of the intervention consisted of three parts: self- reported adherence, knowledge, and perceived barriers to use the guideline. Additionally, participants were asked to score their satisfaction with the course after completing it. Self-reported adherence was measured using the 6 items that were found to discriminate between expert and general physiotherapists in an 18-item questionnaire developed in a previous study. Knowledge on the contents of the guideline was measured by means of a self-developed knowledge questionnaire [20], with 19 items reflecting the content of the guideline recommendations. The score range of each question was 0–4, yielding a total score range of 0–76, with a higher score meaning greater knowledge. | Clinical Practice | Self-reported adherence with process indicators: Table 2 shows that compared to baseline the mean adherence score in both groups improved directly after the educational course at T1 and 3 months thereafter. The improvements at T1 and T2 in the intervention group (both p values50.001) and at T2 in the control group (p value50.001) reached statistical significance. The improvement was however statistically significantly greater in the intervention group than in the control group at both T1 and T2 (p value50.001 and 0.004, respectively). When taking into account all time points with the linear mixed model, a statistically significant difference of the change in adherence score over time was seen in the intervention group as compared to the control group (p ¼ 0.006)  Knowledge: The mean knowledge score increased in both groups, with the improvement from baseline in the intervention group reaching statistical significance at both T1 and T2 (both p values 50.001) (Table 2). The difference in improvement was statistically significantly greater in the intervention group than in the control group at both T1 and T2 (p value50.001 and 0.004, respectively). When taking into account all time points with the linear mixed model, a statistically significant difference of the change in adherence score over time was seen in the intervention group as compared to the control group (p = 0.006). | Qualitized: PTs that engaged in a interactive course on most recent clinical practice guidelines pertaining to OA, demonstrated increase in knowledge and a significant improvement in adherence to the guidelines.  1.11 |  |  |  |  |  |  |
| Petty 2011 | Masters Level Program | Round 1 interview agenda - In what ways, if any, has your clinical practice been affected by the course? - In what ways, if any, has the course affected your career/professional development? - Has the course had an impact on you personally in any way? - Have there been any negative effects of completing the course? - Is there anything else?  Round 2 interview agenda - Pre-course experience - Expectations of the course experience of the course - Impact of the course - Post course activities and development - Is there something else we should discuss?  Round 3 interview agenda - Influence of previous learning on perceived self efficacy to succeed - Perceived self efficacy and identity - Influences on perceived self efficacy - Current learning needs and perceived self efficacy | Clinical Practice Leadership Education Research | Participants enhanced their practice in terms of three interrelated aspects: they gained a critical understanding of practice knowledge that facilitated more patient centred practice, which led to a capability to learn in and from practice. For most participants their enhanced understanding of their practice enabled them to communicate more effectively with patients, ‘You have to have the evidence to know why you are doing that and then you’ve got to then sell that to the patient.. the more you feel you’re confident about something, then the better you’re going to impart that knowledge’(P4). They thus gained enhanced self efficacy in their practice knowledge. Participants’ practice became more deliberate and individualised,‘I used to think all shoulders are the same and all hips are the same. I then saw each patient more as an individual’(P1). Participants enhanced their practice in terms of three inter-related aspects: they gained a critical understanding of practice knowledge that facilitated more patient centred practice, which led to a capability to learn in and from practice. These three aspects of clinical expertise are depicted in Fig. 1 While the large arrowhead indicates the overall direction of development, the small arrow-heads highlight that patient centred practice enhanced understanding, and learning from practice enhanced patient centred practice.  3.2.1. Critical understanding of practice knowledge. Participants gained a deeper understanding of practice knowledge, I (came) to look at everything I did in a much deeper way (P1). They understood why they were asking the questions in the subjective examination and why they were performing the tests in the physical examination, You know exactly why you've done each test and for what reason (P2). Participants demonstrated more overt metacognition in their practice, became less dogmatic and more open to alternative ways to practice, helped me understand there are two or three ways to do something, before I used to think there was, only one way to do something (P10). This suggests participants thinking movedfrom dualism towards relativism (Perry,1970). Enhanced understanding impacted on their treatment and management of patients. They became more deliberate and creative with a greater ability to justify their decisions, I can move logically, but still quite creatively, and can justify what I am actually doing (P7). Once you understand the anatomy, biomechanics, and pathologies, you can then treat anything in any situation (P1). Practice thus became contingent and was viewed as professional artistry (Fish and Coles, 1998). Deep conceptual understanding with integration of all types of knowledge and an ability to critically evaluate knowledge is considered characteristic of expertise (Sandberg, 2000; Benner 2004; Higgs and Jones, 2008) and enabled them to become more patient centred. 3.3. Patient centred practice Patient centred practice is used here to refer to the conscious, deliberate creative and individualised clinical care of patients. The main focus of the course was developing practitioners diagnostic reasoning for patients with physical impairments and this was borne out in the data. Assessment findings were now used to guide treatment choice and gave me the freedom to create treatment techniques to suit me and the patient (P6). Greater depth of understanding and knowledge of the principles underpinning practice enabled them to better manage patients with more complex and recurrent presentations | 1.2 1.3 1.4 1.5 1.6 1.7 1.8 1.11 | Participants questioned and evaluated their practice, no longer assuming they were effective and this then enabled them to learn in, and from, their practice, ‘Each patient is like an individual piece of research, each one adds to your knowledge and experience’ (P5); ‘(the course) teaches you how to learn. You have the basics and you have the patients and then you've got to learn from that.why did that work, why did this not work?’(P6) Over time, this process enhanced their prognostic judgement, ‘I know who I can and can’t help’(P7).  Greater depth of understanding and knowledge of the principles underpinning practice enabled them to better manage patients with more complex and recurrent presentations.  Participants demonstrated more overt metacognition in their practice, became less dogmatic and more open to alternative ways to practice, ‘helped me understand there are two or three ways to do something, before I used to think there was only one way to do something’(P10). This suggests participants’ thinking moved from dualism towards relativism  Participants enhanced their practice in terms of three interrelated aspects: they gained a critical understanding of practice knowledge that facilitated more patient centred practice, which led to a capability to learn in and from practice. | 2.2 2.3 2.7 2.8 2.10 2.11 | The majority of participants commented on a greater ability to learn on their own and this reduced their attendance at weekend courses. Almost all talked about their continued thirst to learn after the MSc and drive to improve their practice, ‘I can always do better and improve myself as a clinician’(P10). Participants became more autonomous learners ‘I do far less weekend courses because I don’t think there are any courses out there that can teach me anything more than I can learn on my own’(P1).  They were able to not only operate in the messy and unpredictable world of clinical practice, but were able to learn from it. This capability to learn in, and from, practice is considered essential not only to develop but also to maintain clinical expertise | 3.1 3.2 | Participants gained enhanced criticality towards research, ‘able to review new literature in a much better way’(P4), ‘I don’t just read the journal and think everything’s absolutely right, I question everything’(P9) and toward their own clinical practice and that of others, they became ‘more confident to challenge myself and other people on diagnosis or progression’(P3). They had become more critically evaluative of research and used research evidence more judiciously so that ‘decisions around assessment and treatment are more accurate and informed, based around the evidence’(P10). Participants demonstrated a shift from uncritical acceptance to critical evaluation and application of propositional knowledge. | 4.2 4.3 4.4 |
| Petty 2011 | Masters Level Program | the primary data were collected through individual interviews. A total of 28 audio-recorded, semi-structured interviews (amounting to 19 hours) were conducted face to face or via the telephone between January 2005 and April 2007. All but one of the participants was interviewed two or three times  The first round of interviews explored their experience completing the MSc course and its influence on their clinical practice. Data analysis was facilitated by role transition theory. | Clinical Practice Leadership Education Research | ‘I found the placements really, really helpful and even though it was very, very difficult, it was probably the most positive thing to come out of the course. In my practice, I’m still using aspects of the clinical reasoning and the key criteria that I learnt on placement and use it in my undergraduate and postgraduate teaching.’ (P7)  ‘I’ve improved my thought processes behind what I’m doing and why I’m doing it; my ability to reflect, my ability to deduce what information that test has given me and the value of that information in terms of the evidence base.’  Participants gained an enhanced understanding of the subjective and physical examination process, and how to make sense and assess the findings from an individual patient examination. They were better able to choose and apply appropriate treatment and management strategies for their patients  Participants adapted their examination, treatment and management strategies to the individual patient, becoming more deliberate and creative. This enhanced their ability to manage people with more complex presentations. They used information from the patient to guide their management. They were critically reflective of their practice, habitually evaluating the effect. | 1.2 1.3 1.4 1.5 1.6 1.7 1.8 1.11 | ‘With my first patient, I was watched and I thought ‘oh no, what are they going to think about me’, but that feeling very quickly disappeared. . .I thought ‘I’m here to learn, I’m here to get something out of this placement, and therefore I want them to look at what I’m doing and say, ‘don’t do it like that, perhaps do it like this’. . .Within 3 weeks, my practice had completely altered, it was such a fantastic experience. In the final week and a half, I was asking my educator to come and watch me. . .and give me their input and feedback.’   Engagement in the process of critical evaluation may also foster a tentative, experimental and sceptical attitude towards practice knowledge [19]. Thus, critical evaluation may enhance both understanding of, and criticality towards,practice knowledge.  Participants adapted their examination, treatment and management strategies to the individual patient, becoming more deliberate and creative. This enhanced their ability to manage people with more complex presentations. They used information from the patient to guide their management. They were critically reflective of their practice, habitually evaluating the effectiveness. | 2.4 2.6 2.7 | ‘I found the placements really, really helpful and even though it was very, very difficult, it was probably the most positive thing to come out of the course. In my practice, I’m still using aspects of the clinical reasoning and the key criteria that I learnt on placement and use it in my undergraduate and postgraduate teaching.’ (P7)  This enabled them to evaluate the effectiveness of their clinical decisions and learn from their patients; in this way, they were able to learn in, and from, their clinical practice: | 3.2 3.8 | They became more critically evaluative of their practice knowledge and that of others, and were better able to justify their clinical decisions to themselves, their patients and other health professionals.  ‘I’ve improved my thought processes behind what I’m doing and why I’m doing it; my ability to reflect, my ability to deduce what information that test has given me and the value of that information in terms of the evidence base.’ | 4.2 4.3 |
| Prizinski 2021 | Residency and Fellowship | Each novice physical therapist (4 participants) was interviewed 2 times (8 total interviews): Beginning (month 1-2) prior to beginning formal mentorship and at the end of program (month 11-12) following completion of mentorship. Each interview was conducted remotely and used an audio and visual recording platform.  two survey instruments that will be used in this study will assist with informing the overall qualitative data collected. Each resident will be administered: (1) the DISC Assessment at the beginning of the program which is a curriculum prerequisite at Nxt Gen Institute to the “coaching” session between Nxt Gen Faculty and the resident-mentor; and (2) the Clinical Reflection and Reasoning (SACRR) at the beginning of the program and at the end of the program. The SACRR has been found to be a valid measure of a physical therapy and occupational therapy student’s development of self-reflection and reasoning skills in a clinical environment  a reflective narrative of each component of the modified coaching program. This included the development and collaboration of the learning modules on Human Engagement and Effective Communication  the results on the SACRR instrument along with interview findings from the mentors to address the development of clinical reflection and reasoning skills toward becoming more patient centered as a result of the coaching program | Clinical Practice Leadership or Management | yes, I do believe that the residency program has definitely built my confidence as when I was a new grad there. I was unsure about a lot of things and I was uncomfortable in that uncertainty. (A.W. page 4)  I feel like I engage them with a lot more confidence on my side of things. I remember that was probably like maybe two or three weeks ago I was starting to actually feel like not like a new grad anymore. I was like, "I got this. I know what I'm doing." (K.B. page 7)  The residents were able to identify their perception of developing clinical reasoning skills through the structure of a residency program which involves communication between a mentor, patients, and reflecting on those interaction  The theme that emerged as Effective Communication was integral to the development of a NPT. This was first observed through the residents perception of how coaching shaped their mentoring interactions, patient interactions, and overall development toward being patient centered.  Our participants were able to demonstrate improvements in the mean pre and post SACRR scores for reflection and reasoning and more specifically the cluster items found by Willis and Colleagues (2018) specifically for “decision-making on experience and evidence, as well as reflection and reasoning” which applied to our residents learning post professionally and not as students in physical therapy school.  results found that 24 of 26 questions on the instrument demonstrated a positive improvement. | 1.3 1.4 1.5 1.6 1.11 | The residents and their mentors identified the importance of Effective Communication through the mentorship experience and understanding how another person’s perspective may be different from their own which aligns with positive professional performance | 2.3 2.8 |  |  |  |  |
| Rebbeck 2006 | Single Encounter Course | Physiotherapist outcomes Physiotherapist knowledge of the guidelines was measured using a custom-made questionnaire developed for this study. Questions included: self-rating of knowledge of the guidelines, treatments currently used to manage whiplash, treatments understood to be evidence-based, when and why physiotherapists refer to other disciplines, treatment goals set for whiplash patients, reporting responsibilities, and understanding of yellow flags (see Appendix 1 which appears as an eAddendum on the journal website). The questionnaire yields a score ranging from 0 to 28, with higher scores indicating greater knowledge of the guidelines.  Physiotherapist clinical practice was measured as the percentage of participating physiotherapists prescribing guideline recommendations taken before and after the trial (from responses to the questionnaire) and during the trial (audited from patient notes). | Clinical Practice | Effect of intervention on patients: there was no significant difference between the implementation patients or the dissemination patients at any follow up for the Functional Rating Index (Figure 2), the Core Outcome Measure (Whiplash), or Global Perceived Effect (Table 3). A breakdown of responses to the separate items of the Core Outcome Measure (Whiplash) is presented in Table 4. In addition, both groups of patients were equally satisfied with care provided by their general practitioner (p = 0.69), their physiotherapist (p = 0.87), and with the consumer version of the guidelines (p = 0.93) (Table 5)  Effect of intervention on physiotherapists: Physiotherapists in the implementation group increased their knowledge of the guidelines by 5.5 points (95% CI 2.5 to 8.4) more than physiotherapists in the dissemination group (p = 0.001). Their self-rated understanding of the guidelines increased by 1.5 points (95% CI 0.7 to 2.3) more than the dissemination group (p = 0.001). Their ability to identify yellow flags (p= 0.02) and their self-reported use of functional outcome measures (p = 0.01) also increased significantly more than the dissemination group (Table 6). Two out of five guideline recommendations were identified by more physiotherapists in the implementation group than the dissemination group at the end of the trial – ‘reassure patient’ (p = 0.05) and 'advise to act as usual’ (p = 0.02) (Table 7). Furthermore, these recommendations were actually prescribed more by the implementation physiotherapists during the trial (p = 0.04 and 0.02) as measured by audit of patient notes (Table 7) | Qualitized: PTs in the implementation education group increased their knowledge of current guidelines, their understanding, ability to identify yellow flags, use of outcome measures and actual implimentation of the guidelines.   1.7 1.8 1.11 |  |  |  |  |  |  |
| Resnik 2004 | Accredited Area of Practice Training | patients completed self-report health status surveys prior to their initial evaluation and following discharge from their rehabilitation episode - Focus On Therapeutic Outcomes, Inc. (FOTO) database  Three HRQL measures were utilized in this study: the FOTO overall health status measure (OHS)16, ,71the SF-12 Physical Component Summary (PCS)', and SF-36 physical functioning scale (PF-10)'. These measures were calculated from 24 HRQL items, which have been fully described yb Hart1 | Clinical Practice | Mean intake and discharge HRQL scores for therapists with and without advanced orthopaedic certification are shown in Table 5. Results of analyses of linear mixed models demonstrate that therapists with MTC had higher outcomes on al three dependent measures (Table 6). There was insufficient data to estimate the effect of dual (AAOMPT/OCS) certification.  Two therapists who had both an OCS and manual training had higher mean patient outcomes in all measures. The effect fo this dual credential was not evaluated in our model due to the small sample size of patients treated by therapists with more than one method of advanced training. The effect of dual credentials should be investigated ni future studies involving a larger sample size. | Qualitized: PTs with AoP training demonstrated higher mean patient outcomes compared to those that did not have the same level of training. 1.7 1.11 |  |  |  |  |  |  |
| Rodeghero 2015 | Residency and Fellowship | Focus On Therapeutic Outcomes is a national patient outcome assessment system (FOTO)  Patients enter demographic information into the system and complete a baseline FS measure prior to their evaluation and receiving intervention. The FS measure requires patients to answer questions about their level of difficulty with various functional activities, as related to the body region requiring treatment.   The final FS score represents an estimate of the patient’s functional level on a scale from 0 to 100, with higher measures representing higher functioning.  Physical therapists using FOTO were surveyed via e-mail to identify level of education and personal demographics.  1) residency program completion, (2) fellowship program completion, or (3) no residency or fellowship training. There was uncertainty about clarity of dual program completion, as “residency” and “fellowship” have not always been clearly defined.36 Any therapist who had reported completing both types of programs (n = 4) was classified into the fellowship-trained group, as this reflects a higher level of post-professional clinical education.  To compare the effectiveness between clinical groups, both patient outcomes and clinical efficiency were dependent variables. Patient outcomes were assessed using the FS change score, which was calculated by subtracting the intake FS score from the FS score at discharge. Clinical efficiency was calculated by dividing the FS change score by the number of treatment sessions. | Clinical Practice Leadership | Overall, patients treated by therapists in the fellowship group showed statistically greater improvements than those treated by the therapists in the groups with residency training or without residency or fellowship training (P<.001)  Overall, therapists in the fellowship group provided more efficient care (greater improvements per number of treatment sessions) than the therapists in the group without residency or fellowship training, followed by the residency group (P = .06 to P<.001).  Last, chi-square analysis revealed a statistically significant association between clinical groups and achieving the MCID at each level of change (χ210 = 28.4, P = .002). This indicates that there was a significant association between the clinical group and the magnitude of the MCID achieved. Patients treated by physical therapists with fellowship training had a tendency to achieve treatment effect sizes of greater magnitude (3 to 4 times the MCID; standardized residual, 1.9), whereas those treated by the residency-trained group did not (standardized residual, –2.5).  The results of this preliminary study suggest that physical therapists with fellowship training may provide better patient outcomes in fewer treatment sessions compared to other physical therapists. Fellowship graduates achieved the greatest amount of FS change in the fewest number of treatment sessions, resulting in a significantly higher efficiency rating than those with residency training or no training | Qualitized: The results of this study appear to support the positive influence that formal postprofessional education in the form of fellowship training may have on clinical practice in the way of greater improvements in outcome scores and greater efficiency of care.  1.7 1.11 | The results of this preliminary study suggest that physical therapists with fellowship training may provide better patient outcomes in fewer treatment sessions compared to other physical therapists. Fellowship graduates achieved the greatest amount of FS change in the fewest number of treatment sessions, resulting in a significantly higher efficiency rating than those with residency training or no training | 2.7 |  |  |  |  |
| De Rooij 2020 | Multiple Encounter Course | Knowledge was tested by a multiple-choice questionnaire which was based on the content of the e-learning topics. comprised 60 multiple-choice (three answer options) questions, which required approximately 60 min of PTs time. Blooms' taxonomy was used to formulate questions on various levels from fac- tual knowledge up to comprehension levels (Stanny, 2016). A correct answer yielded one point with a total range score of 0–60, with a higher score indicating more knowledge.  For measuring clinical reasoning skills, a vignette of a patient with knee OA and comorbidity (diabetes type 2 and coronary dis- ease) was described with nine open questions mainly based on the application – analysis level of knowledge (Stanny, 2016). Vignettes are a valid way to measure clinical reasoning skills by PTs (van Dulmen et al., 2014). The questions focused on clinical decision mak- ing in diagnostics and treatment and contained the following topics: history taking and physical examination of the comorbid disease, interpretation of test results, and adaptations of the OA exercise therapy during treatment. For each question a maximum of five points could be achieved (total range score of 0–45, with higher scores indicating a higher level of skills for clinical reasoning). A stan- dard checklist of predefined answer options was used to assess the output. Two reviewers assessed the answers independently (MROO and MTER). In case of differences in scores, mutual agreement was achieved by discussion. | Clinical Practice | Statistically significant improvements were found for the total group (n = 34), both for knowledge levels between baseline (T0) (mean = 38.65 SD 4.58) and T1 (mean = 43.09 SD 5.69) (p < 0.01), and for clinical reasoning baseline (mean = 23.50 SD 6.37) and T2 (mean = 30.25 SD 6.53) (p < 0.01). | Qualitized: PTs who engaged in an educational course on competence (knowledge and clinical reasoning) demonstrated significant improvements in both fields at time point 2.   1.6 1.11 |  |  |  |  |  |  |
| Rushton 2010 | Masters Level Program | Semi-structured interviews with students and clinical tutors were guided by documentary analysis of course documentation and a survey of current practice regarding clinical placement experience completed two years earlier (Rushton and Lindsay, 2007).  Participant observation of clinical examinations enabled direct engagement of the researchers in the activities of the group being observed (Pope and Mays, 2000), and allowed exploration of the assessment of clinical practice performance; where the construct was used implicitly | Clinical Practice Leadership Education Research | A key component of reasoning was the ability to make decisions regarding further information required from the patient history and/or physical examination to complete the picture for individual patients. The students described a move away from routine collection of all information.  the importance of differential diagnosis to confirm a hypothesis was identified. ‘My knowledge of conditions has actually increased so I am more able to pick up certain patterns’’. (Student L)  Ongoing reasoning was seen as important throughout patient management, and was characterised as a continuous process of prioritisation of issues for the individual patient, based on the available clinical and empirical evidence. Prioritisation was emphasised as a higher-level skill. The ability to think laterally was seen as essential. The component of ‘high level of self-analysis’ assisted the recognition of personal limitations and effective metacognitive skills, both being characteristic of experts  The integration of knowledge with practice was seen as essential emphasising propositional knowledge, and some reference to process knowledge. All aspects of the QAA and JQI descriptors are reflected in the findings, including: dealing effectively with complex issues, the making of sound judgements, communicating conclusions, decision-making in complex situations through the components of clinical reasoning/justification of decisions; originality and creativity in the component of creative practice; commitment to Continuous Professional Development(CPD); and independent learning ability and self-direction implicit through multiple components, particularly ‘high level of self-analysis’. a patient-centred approach with a confident and self-analytical approach to practice. ‘‘Being more adaptive to the person and much more sensitive to their needs., the subjective picking up on how it is impacting upon their life and how that then drives your objective and.management’’. (Student K) | 1.2 1.3 1.4 1.5 1.6 1.8 1.11 | Personal characteristics The importance of evaluative and reflective use of experience (Schon, 1983), was illustrated by the component of ‘high level of self- analysis’ and the constant cognitive functioning required for the component of ‘adaptability’ and the subcomponents of ‘prioritisation’, ‘lateral thinking’, ‘ability to deal with multiple issues at once’, ‘ongoing process of reasoning’, ‘ongoing process of evaluation’ and ‘adaptability of skills’; where the ability to reflect-in-action is implicit.   The component of ‘high level of self-analysis’ assisted the recognition of personal limitations and effective metacognitive skills, both being characteristic of experts   Practitioner independence was implicit throughout the case study as was, ‘high level of confidence’ | 2.3 2.6 2.7 2.8 | Personal characteristics The importance of evaluative and reflective use of experience (Schon, 1983), was illustrated by the component of ‘high level of self- analysis’ and the constant cognitive functioning required for the component of ‘adaptability’ and the subcomponents of ‘prioritisation’, ‘lateral thinking’, ‘ability to deal with multiple issues at once’, ‘ongoing process of reasoning’, ‘ongoing process of evaluation’ and ‘adaptability of skills’; where the ability to reflect-in-action is implicit.   All aspects of the QAA and JQI descriptors are reflected in the findings, including: dealing effectively with complex issues, the making of sound judgements, communicating conclusions, decision-making in complex situations through the components of clinical reasoning/justification of decisions; originality and creativity in the component of creative practice; commitment to Continuous Professional Development(CPD); and independent learning ability and self-direction implicit through multiple components, particularly ‘high level of self-analysis’. | 3.1 3.2 | in addition, the importance of evidence from research to inform propositional knowledge was emphasised.  ‘‘Masters level – up to date awareness of all evidence based information relevant to area of speciality’’. (Clinical Tutor R)  justification of decisions as important to the construct. The stronger students voluntarily linked to the literature when answering questions, articulating justification of their decision-making. ‘‘The literature suggests the deep neck flexors have a positive effect on headache according to Jull, 2002’’. (Participant Observation 2)  Ongoing reasoning was seen as important throughout patient management, and was characterised as a continuous process of prioritisation of issues for the individual patient, based on the available clinical and empirical evidence. | 4.2 4.3 |
| Schreiber 2012 | Multiple Encounter Course | All attendees at the conference completed a survey on knowledge and frequency of use of pediatric tests and measures.  The survey was reviewed for face validity by several experienced pediatric PTs prior to the start of the project. Information on measures of impairments of body structure and function, activity, and participation was presented at the conference, and the survey was designed to gather information about knowledge and frequency of use of measures in each area.  At the end of the 16-week time frame, the individuals completed the survey again and also had the opportunity to provide additional narrative comments about the implementation of the KT plan. | Clinical Practice | Statistically significant differences were noted in the knowledge for selecting (P = .000), administrating (P = .024), interpreting (P = .000), and sharing (P = .000) standardized tests and measures. When analyzing changes in the frequency for which these participants did each of these elements, only frequency of interpreting tests and measures was statistically different from baseline to post- survey (P = .026). | Qualitized: pediatric PTs who engaged in continuing education conference on knowledge and frequency of use of tests and measures demonstrated significant improvements in knowledge of selecting, adminsitering and understanding standardized tests and measures in pediatrics.   1.4 1.11 |  |  |  |  |  |  |
| Seif 2019 | Multiple Encounter Course | to assess the first objective, the therapists who enrolled in the course series were administered an anonymous and voluntary pre-course and post-course online survey that assessed their comfort and confidence in working with patients with primary spinal dysfunctions. The pre and post-surveys each included two questions. The first question assessed participants’ comfort level working with patients with spinal dysfunctions prior to (pre-survey) or after (post-survey) attending the course series. The second question assessed participants’ confidence level working with patients with spinal dysfunctions prior to (pre-survey) or after (post-survey) attending the Manual Therapy Course series. Responses were on a Likert scale (very comfortable/confident, comfortable/confident, not comfortable/confident). Participants also had the opportunity to enter free text comments for each question. To assess the second objective, we utilized data that were already being compiled at Mercy on the PTs utilization of standardized outcome meausres. The data were tracked in Microsoft Excel and organized using a pivot table. We obtained de-identified data (i.e., patient identifiers removed) from Mercy for the 6 months prior to beginning the course series and for the 6 months following the final course of the series. We used the data to calculate pre- and post-course series utilization of standardized patient outcome measures. We also calculated how many patients overall had outcomes collected at both the initiation and conclusion of the episode of care (completed discharges). | Clinical Practice | We had a response rate of 58% on the pre and post-surveys. Prior to the start of the course series, 28.57% of respondents were ‘not comfortable,’ 50.0% ‘comfortable,’ and 21.43% were ‘very comfortable’ working with patients with primary spinal dysfunctions. When assessed 6 months after the course series, 57.14% of the respondents were ‘very comfortable’ and 42.86% were ‘comfortable’ (Fig. 1). When asked about their confidence in working with patients with primary spinal dysfunctions, 71.43% of the respondents were ‘confident’ and 28.47% were ‘not confident’ before taking the course series. After the course series, 64.29% of the respondents were ‘confident’ and 35.71% were ‘very confident’ (Fig. 2).  The number of PTs tracking outcomes pre- and post-course series went from 6 to 19 of the 24 clinicians in the course series, and the number of completed discharges went from 32 to 68. PTs in this quality improvement study increased their utilization of standardized outcome measures which is one component of current practice guidelines. Additionally, the PTs in this pilot study demonstrated an increase in their self-perceived comfort and confidence in their comfort and confidence in working with patients with spinal dysfunctions. | Qualitized: the PTs in this study demonstrated an increase in their self confidence in their comfort and confidence in working with patients with spinal dysfunctions, they also increased their utilization of standardized outcome measures.   1.11 |  |  |  |  |  |  |
| Shalabi 2024 | Single Encounter Course | Data were collected using a questionnaire-based survey that was divided into four sections. The first section gath ered demographic information about the participants, including age, gender, nationality, residency, marital status, highest medical degree, number of years of PT practice (work experience), number of months they had been  enrolled in an OCME program, and their computer skill level. The second, third, and fourth sections measured  the participants’ attitudes toward OCME programs, their satisfaction with the OCME programs, and the impact  of these programs on their clinical practice, respectively. Participant satisfaction was assessed via nine questions  pertaining to their satisfaction with the OCME programs’ overall quality, time, schedule flexibility, content, inter actions, tutor support, answering of questions, practical utility, and overall satisfaction. The participants’ attitudes  towards OCME were assessed via seven questions related to their perceptions about the flexibility and difficulty of hese programs compared to traditional CME programs, the possibility that these OCME programs might fully or partially replace face-to-face traditional CME programs, whether they wished to be enrolled in similar OCME programs, whether they would recommend these programs to their colleagues, and whether they preferred face-to-face programs. The impact of the OCME program on their clinical practice was assessed via four questions about their perceptions about the extent of contribution of the program they are/were enrolled for in improving their medical knowledge, clinical skills, patient outcomes, and their confidence in managing patients. For statements related to attitudes and impact of the OCME programs, participants were asked to indicate their level of agreement on a Likert scale of 1 to 5 (“strongly disagree,”“strongly agree,” “neither agree nor disagree,” “disagree,” or “agree”). For the statements related to satisfaction, they also indicated their response on a Likert scale of 1 to 5 (“strongly dissatisfied,” “strongly satisfied,” “neither satisfied nor dissatisfied,” “dissatisfied,” or “strongly dissatisfied”). | Clinical Practice | Over 70% of the PTs “strongly agreed” and “agreed” with the statement “attending an OCME program improved my medical knowledge,” while 62.2% and 59.1% “strongly agreed” and “agreed“ with the statements “attending an OCME program improved my patient outcomes” and after attending an OCME program, I feel confident to manage my patients,” respectively. With regard to the remaining item (“attending an OCME program improved my clinical skills”), only 46.5% reported that they “strongly agreed” and “agreed.” These responses indicate that the participants felt that their practice was mostly positively impacted by OCME (Table ) | Qualitized: PTs who attended OCME tended to agree with statements that OCME improved medical knowlegde, improved patients outcomes and overall positively impacted their practice.   1.11 |  |  |  |  |  |  |
| SjodahlHammarlund 2013 | Masters Level Program | Written Self Reflection analysis  Data triangulation included the self-reflections and the course evaluation surveys from the three courses (74% response rate). Each course evaluation was filled out anonymously after course completion, and contained ratings and possibility for free text comments. Only the free text comments were included in the analyses, which entailed the participants’ views on learning and progress, course structure, educational and global relevance. | Clinical Practice Leadership Education Research | Autonomy emerged as the main theme. Autonomy emerged as the latent meaning of the categories. It can be said that self-development includes the ability to take charge of one’s own learning and the use of effective learning strategies (18). In the context of our study, we interpreted the sense of increased self-efficacy and the construction of knowledge, and understanding and skills related to clinical practice, to constitute important parts of autonomy as an extended, in-depth professional competence.  Developing understanding, knowledge and skills The participants described having gained a further under-standing of their prior knowledge. The working structure was perceived as being enhanced by their new skills and experiences. Some also expressed a new dimension of insight when communicating with others.  Recognizing increased self-confidence and self-efficacy. The participants expressed increased self-confidence and self-efficacy during the learning process. They described increased abilities in making judgments leading to a broader perspective and a sense achievement with the process, steps and actions taken. "I feel more secure now ..."  Interacting with other health professionals in clinical practice. The participants expressed that they wanted to share their new insights with colleagues and other health professionals by initiating discussions and to make changes in new direction | 1.1 1.3 1.5 1.6 1.9 1.11 | Some participants choose to challenge themselves further by deliberately stepping out of their comfort zone. "I choose this assignment in order to challenge myself ... I also wanted to stand in front of my fellow students and talk about something that I find difficult."  Transfer and implementation of knowledge and skills. The participants expressed that working on their assignments inspired them to transfer their knowledge into the clinic and suggest their ideas to other health professionals.  For an active strategy to succeed it is necessary to involve the leaders and the colleagues. Interacting with other health professionals in clinical practice. The participants expressed that they wanted to share their new insights with colleagues and other health professionals by initiating discussions and to make changes in new direction  Autonomy emerged as the main theme. In the context of our study, we interpreted the sense of increased self-efficacy and the construction of knowledge, and understanding and skills related to clinical practice, to constitute important parts of autonomy as an extended, in-depth professional competence. | 2.4 2.6 2.7 2.10 2.11 | The participants specifically stressed the value of learning from and with peers when discussing articles and methodological issues.  Their prior experiences were mentioned and highly valued. They were also able to identify their own progress and learning needs.  This extended conversation was recognized as challenging in motivating and supporting colleagues. "A great challenge for my future work will be: how do I get my colleagues in the clinic to be curious. How do I get them to read articles, be willing to discuss their doubts in an open and learning environment and to reflect and implement new strategies? How do I create an open and learning environment?"  So in the future I hope that we can inspire and learn from each other. | 3.1 3.4 3.5 3.7 3.8 | Creating a structure.The participants expressed having realized the importance of being specific regarding the objectives of the assignment and defining the purpose when structuring the process.The process contained small steps that were identified and where minor changes could make a difference. When looking back on how they had planned and structured their assignments, they became aware that if the initial steps were planned in detail it would enhance the quality and efficacy of their research. If I would do this again I would be more specific about my objectives. [I would] ...break down the question even more. More focus.  I have also got a greater understanding for the importance of validity as a concept and its different approaches. | 4.1 4.2 4.3 4.5 4.8 |
| Smith 1999 | Residency and Fellowship | An outcomes questionnaire was developed in 1993 by a research team to gather data on graduates from the Kaiser residency program. This questionnaire was pilot tested utilizing graduates of yearlong Australian residencies in manual therapy. In the present study, our questionnaire was modified to improve clarity of questioning and to better assess the program's influence on clinical practice | Clinical Practice Leadership Education Research | Graduates spend 75% of work time in patient care  Influence of residency training on clinical skills received high major positive ratings on the abilities to examine thoroughly (95%), reason logically (94%), treat effectively (83%), treat efficiently (79%), "diagnose" (85%),treat complex patients (86%),and on overall patient management (68%)  graduates ranked "ability to use a logical clinical reasoning process" (29%) as the single greatest benefit of residency training on their career. | Qualitized: Residency graduates attribute their training to major improvements in patient examination, clinical reasoning, treating effectively/efficiently and overall patient management.   1.4 1.5 1.6 1.7 1.8 1.11 | A positive influence (major positive plus some positive) on number of patient referrals (61%) and number of professionals who refer patients for care (70%) was reported  Most graduates reported a positive influence (major positive plus some positive) of residency training on job opportunity (80%), promotion (60%), and salary (76%)  A positive influence (major positive plus some positive) was reported on communication with health personnel (90%) and on communication with patients (95%  Since completing residency education, presentations had been made by 15% of graduates at the national level and by 30% at the district or sectional level.  Kaiser residency graduates report a positive influence of residency training on their ability to communicate with patients and other health personnel (Table 3). The positive influence on referrals suggests that other health professionals value the clinical expertise of graduates  Residency graduates appear to place value on professional organization membership and involvement. Eighty percent of graduates are APTA members, whereas only 48% of physical therapists nationally are member | Qualitized: Residency training demonstrates a positive influecne on patient referrals, greater job opportunities and promotions. As well as ability to communictae with other HCPs and confident to speak publically.   2.2 2.5 2.7 | 26% are clinical educators  The one-on-one clinical mentoring experience with expert clinicians provides a strong role model for graduates to emulate. From the results of this survey it appears that graduates maintain a high level of involvement in teaching and consultation, spending 23% of their time in this area. Also, many (60%) have secondary positions, 40% of which are in areas of clinical education or clinical consultation (as clinical specialists). | 3.8 | Graduates reported a positive influence (major positive plus some positive) of residency training on their ability to use scientific literature as a rationale for physical therapy interventions (94%) and to read and evaluate reports of research critically (86%) (Ta- ble 4). A positive influence in publications (writing case studies, experimental studies, peer-reviewed arti- cles, book chapters, and articles on clinical topics) since completing residency training was reported by 31%  36% of graduates read and discuss reports of research; 11% are involved to some extent in the design, conducting, and reporting of research; 7% reported "other" involvement  36% report an increase in publication (write case studies, experimental stud- ies, peer reviewed articles, book chapters, articles on | 4.1 4.2 4.3 |
| Souter 2019 | Residency and Fellowship | [Five case-based surveys were developed by 2 licensed PTs with residency and fellowship post-professional training, manual therapy certifications, American Board of Physical erapy Specialities (ABPTS) board certifications in orthopedics (OCS), 15 years of combined residency teaching experience, and 8 years of combined academic teaching experience. Common clinical diagnoses that are found in outpatient orthopedic practice were used as the basis for each case.  Each survey consisted of 12–14 items that provided the participants with progressively more subjective and objective information. With each item, the participants were asked to select the most likely diagnosis from a list of diagnoses provided (Appendix 1, Supplemental Digital Content 1, http://links.lww. com/JOPTE/A62 to view case 1). If unable to make a definitive choice at any point in the survey, participants were able to select as many diagnoses as the participants deemed necessary. Instructions informed participants that the purpose of the study was for the participant to determine a single diagnosis as soon as the participant felt comfortable doing so. At the end of the survey, participants had all information pertaining to the case to assist in choosing a final diagnosis or diagnoses.](http://links.lww/) | Clinical Practice | Associations Between American Board of Physical Therapy Specialities and Diagnostic Reasoning Participants with any type of ABPTS demonstrated significantly greater diagnostic accuracy (Chi square, P < .05) and efficiency (in-dependent t test, P < .05) in 3 of the 5 cases when compared to participants with no specialty (Tables 2 and 3). Participants’ results in cases 2 and 3 did not reflect a significant difference in diagnostic accuracy and efficiency between those with and without a specialty. Participants with a specialty demonstrated a significantly (Chi square, P < .05) higher incidence of funneling in cases 1 and 2 (Table 4). Participants with an OCS demonstrated significantly greater diagnostic accuracy (Chi square, P < .05) and efficiency (independent t test, P < .05) in 4 of the 5 cases when compared to participants without an OCS (Tables 2 and 3). ose with OCS and without OCS did not demonstrate a significant difference in diagnostic accuracy (Chi square, P < .05) and efficiency (independent t test, P < .05) in case 2. Participants with an OCS demonstrated a significantly (Chi square, P < .05) higher incidence of funneling in case 1 (Table 4). Associations Between Residency and Fellowship and Diagnostic Reasoning Participants with residency training demonstrated significantly greater diagnostic accuracy (Chi square, P < .05) in 3 of the 5 cases (Table 2). Participants with residency training demonstrated significantly higher diagnostic efficiency (independent t test, P < .05) in 2 of the 5 cases (Table 3). Participants with residency training showed no difference in the frequency of funneling (Table 4). Participants with fellowship training demonstrated significantly greater diagnostic accuracy (Chi square, P < .05) in 2 of the 5 cases (Table 2). Participants with fellowship training demonstrated significantly greater diagnostic efficiency (independent t test, P <.05) and a significantly (Chi square, P < .05) higher incidence of funneling in only 1 of the 5 cases (Tables 3 and 4). Associations Between Other Factors and Diagnostic Reasoning Participants with a Doctor of Physical erapy (DPT) degree demonstrated significantly greater diagnostic accuracy (Chi square, P < .05) in only 1 of the 5 cases and demonstrated significantly greater diagnostic efficiency (in-dependent t test, P < .05) in only 2 of the 5 cases when compared to other PT degree levels (Tables 2 and 3). Participants with a DPT degree showed no difference in the amount of funneling (Table 4). Participants with >10 years of experience, >50% workload in direct patient care, with APTA Clinical Instructor certification, and participation in higher education did not demonstrate significant difference in diagnostic accuracy (Chi square, P > .05), diagnostic efficiency (in-dependent t test, >.05), or incidence of funneling (Chi square, P > .05) in any of the5 cases (Tables 2, 3, and 4). | Qualitized: according to this study PTs with residency or fellowship training demonstrate significantly greater diagnostic accuracy compared to those without the further training.   1.4 1.6 1.11 |  |  |  |  |  |  |
| Stathopoulos 2003 | Masters Level Program | Discussion focus: An exploration of the impact of undertaking study at master’s level for practising physiotherapists. Key areas for discussion to include: 1. Impact of master’s level study on your career. 2. Impact of master’s level study on clinical practice. 3. Impact of master’s level study on other areas of your life | Clinical Practice Leadership Education Research | The acquisition and utilisation of new skills, as well as the refinement and development of already existing skills, was articulated as a major influential effect of having undertaken study at master’s level. The development of skills, such as criticality, analysis, communication and research related skills, made participants feel more confident, more able to deal with complex situations within the work-place and better able to adopt a strategic approach to the development of clinical and organisational practice.  Development of Expertise ‘My practice has changed as a result of the master’s .... it has become much more critical and much more analytical then it was before.’  ‘... Not just listening to the patient’s viewpoint, but acknowledging that as a central starting point really. To become an expert as a physiotherapist isn’t just about developing more and more skills or techniques, ... it is about really empathising with the patients, to be able to deliver for them what it is that they need, from their perspective.’ The study’s findings revealed the immense impact of master’s-level study on the development of participants’ expertise.  Participants clearly demonstrated the development of expertise in their areas of practice. Although the broad scope of the study did not allow for deeper exploration in this respect, the perceptions of participants and the articulation of their experiences showed that they had achieved a level of practice above that of competence and proficiency. They demonstrated the adoption of a patient-centred approach in their practice, where the patients’ needs, wants and expectations were of central importance at all stages of the patient-therapist interaction.   The participants of this study also articulated elements of their clinical reasoning strategies that demonstrate similarities with models of expert reasoning. In addition, the detailed accounts and the emphasis placed by participants on the enhancement of specific skills, such as evidence-based practice and expert reasoning, demonstrates direct ways in which their clinical practice had developed as a result of undertaking master’s level study.  This increase in confidence was mainly attributed to their enhanced ability in the workplace, their increased credibility in front of others and the sense of achievement that was developed as a result of their master’s level study. | 1.2 1.4 1.5 1.6 1.7 1.10 1.11 | Life-long Learning and Career Progression: ‘It made me much more confident that I could do things ... which I never would have dreamt of doing prior to the master’s.’ ‘It has opened so many doors.’ These were also skills that made participants more able and better qualified for higher posts either within or outside the clinical setting, thus enhancing their career progression.  This increase in confidence was mainly attributed to their enhanced ability in the workplace, their increased credibility in front of others and the sense of achievement that was developed as a result of their master’s level study. Participants also perceived this confidence as providing motivation for development, and contributing to a general feeling of well-being in their professional and personal lives, which should not be underestimated A Driver for Change ‘It’s a big challenge doing a master’s ... and you realise that you can go on to other challenges and cope with those as well.’ ‘You are looked up to for a wise opinion, and people have increased expectations of you.’ The physiotherapists of the present study demonstrated an ability to adapt to changing situations and felt confident in the future of the profession. Although they had been through ‘a loss of faith in the profession’, they articulated that their master’s study helped them to regain this faith. They did not perceive physiotherapy as a ‘threatened profession’ any more, but instead viewed it as a ‘changing profession’. The master’s programme not only made them aware of this change but it also made them aware of the need for their active contribution to this change. Thus the restoration of confidence and the reduction of threat facilitate the development of effective practice in a multi-disciplinary environment. They expressed the desire to implement the changes that they had been learning about during the course of their master’s study and to develop practice on an individual and organ- isational level, always within the clinical context. Even the inclusion of research and teaching in their scope of practice was related to their clinical role. They articulated a constant process of questioning of their own and others’ practice, and furthermore were self-directed and self-motivated in looking at how to develop and achieve best practice. | 2.2 2.3 2.4 2.5 2.7 2.8 2.9 2.10 2.11 | Life-long Learning and Career Progression  The participants of the present study clearly showed a deep lifelong approach towards learning. They articulated a constant process of questioning of their own and others’ practice, and furthermore were self-directed and self-motivated in looking at how to develop and achieve best practice.  They expressed the desire to implement the changes that they had been learning about during the course of their master’s study and to develop practice on an individual and organisational level, always within the clinical context. Even the inclusion of research and teaching in their scope of practice was related to their clinical role.  They also were able to asses their learning needs, and perceived learning as being a life-long process that is something to be disseminated and shared with others in a multi-disciplinary context. | 3.1 3.2 3.3 3.4 3.5 3.6 3.7 3.8 | The acquisition and utilisation of new skills, as well as the refinement and development of already existing skills, was articulated as a major influential effect of having undertaken study at master’s level. The development of skills, such as criticality, analysis, communication and research related skills, made participants feel more confident, more able to deal with complex situations within the work- place and better able to adopt a strategic approach to the development of clinical and organisational practice. ‘I felt that I was able to explore evidence-based practice ... and that was something that terrified me before.’  In addition, the detailed accounts and the emphasis placed by participants on the enhancement of specific skills, such as evidence- based practice and expert reasoning, demonstrates direct ways in which their clinical practice had developed as a result of undertaking master’s level study.  They expressed the desire to implement the changes that they had been learning about during the course of their master’s study and to develop practice on an individual and organisational level, always within the clinical context. Even the inclusion of research and teaching in their scope of practice was related to their clinical role.  ‘I felt that I was able to explore evidence-based practice ... and that was something that terrified me before.  ‘My practice has changed as a result of the master’s .... it has become much more critical and much more analytical then it was before. My faith in the clinical effectiveness of physiotherapy, that was lost for various reasons, was regained as a result of the master’s.’ | 4.2 4.3 4.4 |
| Stevenson 2006 | Single Encounter Course | The primary outcome measure for this study was change in physiotherapists’ clinical practice. This was measured using a standardized ‘discharge summary’ questionnaire, which was completed at the end of the episode of patient care by the intervention and control groups  The questionnaire was developed using a modified delphi technique (Robson 1997). Piloting was undertaken by secondary care physiotherapists and the questionnaire was refined as appropriate to ensure ease of completion and eliminate item ambiguity: which approaches do you use to manage patients with acute and chronic low back pain?’ ‘what factors do you feel affect recovery?’ ‘what other skills do you use in managing patients with low back pain?’. | Clinical Practice | ‘Time spent’ Table 2 summarizes the data for ‘time spent’. Prior to training the most popular therapies used by the physiotherapists were ‘home exercise programme’, ‘postural advice’ and ‘hands-off manual therapy’. Psychosocial therapies concerning advice on returning to usual activity and increasing activity levels were also quite commonly used. There was infrequent reported use of ‘muscle imbalance exercises’, ‘paced exercise progression’ and ‘taping’ before training (see Table 2). Post training, there was little change in reported use of therapies. The most common therapies in use were still ‘home exercise programme’, ‘postural advice’ and ‘hands-off manual therapy’. In the control group more use was made of ‘encourage to undertake activities themselves’ and ‘hands-off manual therapy’ and less reported use of ‘home exercise programme’ and ‘change attitudes/beliefs about pain’. The control group was significantly more likely to use ‘acupuncture’, ‘encourage to undertake activities themselves’ and give ‘postural advice’ compared with the intervention group. In terms of the psychosocial therapies, the intervention group was more likely than the control group to give ‘advice to increase activity level’ and ‘change attitudes/beliefs about pain’ but was less likely to ‘encourage to undertake activities themselves’. Figure 1 illustrates the reported percentage ‘time spent’ using the different therapies.  ‘Importance’ Table 3 and Fig. 1 summarize and illustrate the data for ‘Importance’. Results prior to and post training were very similar to those for ‘time spent’ in both study groups. Therapies considered by the physiotherapists to be most important were ‘home exercise programme’, ‘postural advice’ and ‘hands-off manual therapy’. Modalities included in the educational sessions showed little change following the intervention. The control group were significantly more likely than the intervention group to perceive that ‘acupuncture’, ‘encourage to undertake activities themselves’ and ‘postural advice’ were more important modalities post training. ‘Paced exercise progression’ was considered to be more important by the intervention group than the control group post training, though this result was not statistically significant (seeTable 3) | Qualitized: time spent and importance placed on evidence informed practice was more likely in the intervention group.   1.11 |  |  |  |  |  |  |
| Stevenson 2020 | Residency and Fellowship | The three trainees were encouraged to keep a reflective diary of their experiences throughout the course of the 12-month programme. Towards the end of the programme, each trainee was then asked to write an individual ‘case history’ that captured their experiences, challenges and observations of undertaking the programme. No word limits were applied. The case histories were then explored by a member of the development team (KS) for common themes using content analysis (Colaizzi, 1978). These were independently verified by a senior clinician and experienced qualitative researcher (SR). The results of this analysis could be reflected upon by those who developed the programme and used to further strengthen and enhance its content and delivery in the future | Clinical Practice Leadership Education Research | eme 2- Learning new skills Each of the trainees recognized the need for and the challenge of developing the news skills required for this role. This focused primarily on injection skills: ‘I have found learning how to do injections more of a difficult process than I initially thought I would. We started to get exposure to this earlier than the development post timescale had originally stated, which was good in some ways but for me personally, in hindsight, I feel I should have maybe waited until I had got a firm rooting on the clinic appointment timings and content first.’ T1Trainee 2 also identified injection therapy as a new skill to be developed: ‘A clear competency pack was developed, training and supervised sessions organized. Something that was unnerving at the start became very familiar, very quickly. Constructive, non-threatening feedback and support being key.’ Image interpretation was also highlighted as an area for development: ‘We have regular meetings with the musculoskeletal radiologists and a couple of training sessions have been arranged. This has helped improve my confidence’ T2. Trainee 3 identified that linking ‘subjective, clinical and radiological findings in complex cases to guide a patient through the most appropriate management plan’, was an area that improved over the 12 months  emes 3 Working and communicating in a different way Trainees acknowledge the need for a change in the way they worked and also in the way they communicated. The context in which they worked required a change: ‘I found it difficult initially going from working with a physiotherapy treatment mind-set to more of a triage focussed mind-set. There is a considerable difference in the way that I used to work to the way that I now work and this has taken some time to get used to.’ T1. This quote illustrates that there is a difference in the critical thinking required when in treatment focused post to that required in a diagnostic one. Trainees also felt they were communicating in a different way to patients, illustrated by Trainee 2,’ ‘It is not only doctors we communicate to differently within these roles. Discussing findings with patients face to face is much easier. You get an idea of the patients understanding and can judge the pace of the conversation and the complexity of the content.’ They also recognized the importance of different styles of communication: ‘One area that was particularly apparent was the importance of considered language, both spoken and written, and while this was daunting, it also provided an opportunity to truly influence patient care and understanding.’ T3 | 1.2 1.3 1.4 1.5 1.6 1.7 1.9 1.11 | ‘Personally I found the change in role engaging. Moving from a position of seniority, holding the solutions to problems, to a trainee role where I possessed more questions than answers, was a big change, but a refreshing one that allowed a period of reflection on past experience and realization of new development needs’. T3.   Working and communicating in a different way Trainees acknowledge the need for a change in the way they worked and also in the way they communicated. The context in which they worked required a change: ‘I found it difficult initially going from working with a physiotherapy treatment mind-set to more of a triage focussed mind-set. There is a considerable difference in the way that I used to work to the way that I now work and this has taken some time to get used to.’ T1. This quote illustrates that there is a difference in the critical thinking required when in treatment focused post to that required in a diagnostic one. Trainees also felt they were communicating in a different way to patients, illustrated by Trainee 2,’ ‘It is not only doctors we communicate to differently within these roles. Discussing findings with patients face to face is much easier. You get an idea of the patients understanding and can judge the pace of the conversation and the complexity of the content.’ They also recognized the importance of different styles of communication: ‘One area that was particularly apparent was the importance of considered language, both spoken and written, and while this was daunting, it also provided an opportunity to truly influence patient care and understanding.’ T3 I have had involvement in audit, best practice reviews, updating clinical algorithms, teaching and shadowing’ | 2.3 2.4 2.5 2.7 2.10 2.11 | They also recognized the importance of different styles of communication: ‘One area that was particularly apparent was the importance of considered language, both spoken and written, and while this was daunting, it also provided an opportunity to truly influence patient care and understanding.’ T3.   T1; ‘the opportunities available within this (programme) are endless. There are links with academia and research, as well as opportunity for shadowing specialities and teaching roles. I have had involvement in audit, best practice reviews, updating clinical algorithms, teaching and shadowing’. Our team has a number of staff who have joint appointments with Keele University and the opportunities that arise from these is clear;’ ‘The service has strong links with a local university; this university is involved in world leading medical research. Therapists and academics developed a model known as critically appraised topics. These aim to formulate new research questions directly from front line clinicians and answer those questions based on current best evidence. Participation within these groups is very useful.’ | 3.3 3.8 | eme 6 The Future and opportunities The trainees experienced a wide variety of opportunities within their 12 months and could see the value of these experiences, illustrated by T1; ‘the opportunities available within this (programme) are endless. There are links with academia and research, as well as opportunity for shadowing specialities and teaching roles. I have had involvement in audit, best practice reviews, updating clinical algorithms, teaching and shadowing’. Our team has a number of staff who have joint appointments with Keele University and the opportunities that arise from these is clear;’ ‘The service has strong links with a local university; this university is involved in world leading medical research. Therapists and academics developed a model known as critically appraised topics. These aim to formulate new research questions directly from front line clinicians and answer those questions based on current best evidence. Participation within these groups is very useful | 4.1 4.2 4.3 4.4 4.5 |
| Swinkels 2015 | Multiple Encounter Course | The first two outcome variables for the study—(1) physiotherapists’ general attitude toward measurement instruments and (2) their ability to choose measurement instruments—were measured using a five-point Likert scale (ranging from 1 ¼ strongly agree, indicating positive attitude and knowledge, to 5 ¼ strongly disagree, indicating negative attitude and lack of knowledge). The third outcome variable, participants’ use of measurement instruments, was determined by asking participants to estimate with what percentage of their clients they used measurement instruments (none of every five patients [0%], one of every five patients [20%], two of every five patients [40%], three of every five patients [60%], four of every five patients [80%], or five of every five patients [100%]). Participants in the intervention group were asked about the applicability of the tailored educational programme and changes achieved in physical therapy practice; the data were quantified in terms of the percentage of respondents who agreed and disagreed with survey items relating to these outcomes. | Clinical Practice | After completing the educational programme, the intervention group scores were significantly more positive on all aspects of the post-measurement survey, and the control group showed no change.  Was the content of the educational programme useful? Yes - 164 (91%) Did you change your physiotherapy management? Yes - 149 (82%) | Qualitized: Overall, there was a significant increase in the consistent use of outcomemeasures (from 26% at baseline to 41% at follow-up) in the PTs that did the course.  1.4 1.11 |  |  |  |  |  |  |
| Synnott 2016 | Multiple Encounter Course | Semi-structured telephone and Skype interviews were completed by a researcher (AS) who was unknown to the participants and was guided by a flexible question route. The questioning route covered: changes in practice as a result of CFT training; the participant’s confidence and competence in identifying, discussing and addressing cognitive, psychological and social factors with patients; and the participant’s confidence in establishing a strong patient-therapist alliance. Interviews lasted from 45 minutes to 1 hour in length. Interviews were recorded using computer audio softwarea and audio taped with a voice recorder.  During the interviews the researcher took notes, as needed, and statements of relevance and contextual field notes were written verbatim. This aided in the identification of the point of data saturation, as it was evident when no new material or concepts arose.27 Data saturation was achieved after the completion of 11 interviews, with 13 conducted in total. At the conclusion of each interview, the researcher debriefed the participant on the main content of the interview, and time was permitted for any additional commentary to facilitate the emergence of new unanticipated information. | Clinical Practice Leadership | Theme 1: Self-reported change in understanding and attitudes New understanding of the multidimensional nature of pain. Many participants stated that CFT training improved their understanding of the multidimensional nature of pain, as prior to training, a biomedical approach to treatment dominated their practice. But the cognitive part has been the greatest change... understanding the influence of sleeping poorly, being stressed... I mean back then [before training] I probably realised it somewhere in the back of my head but I didn’t act on it. (P6) In recognition of the multifactorial nature of pain, participants reported a change in practice where they now consistently explored cognitive, psychological and social dimensions of a patient’s pain and were cognisant of the importance of promoting the patient’s understanding of pain. Previously I didn’t have an awareness of the psychosocial factors...Now I systematically explore stress, fear, catastrophising, worrying about life, belief in the future, readiness to change. (P4) Heightened awareness of the influence of patient beliefs and expectations. In several interviews, participants acknowledged the influence of patient beliefs that often made the identification and management of cognitive, psychological and social factors challenging, including rigid biomedical belief systems among patients. Specific participant codes, eg, (P1, P2) have been omitted from the table of demographics to ensure confidentiality and anonymity due to the small pool of specialised physiotherapists available for recruitment. It should not be assumed that order of appearance in the table relates to participant numbering.  Themes and categories constituting each theme: Self-reported changes in understanding and attitudes; Self-reported changes in professional practice; Scope of practice; Increased confidence and satisfaction. New understanding of the multidimensional nature of pain; Heightened awareness of the influence of patient beliefs and expectations; Increased awareness of the importance of the therapeuticalliance; Adoption of new screening tools; Altered communication style; Adoption of a functional behavioural approach; Expanded role of the physiotherapist; Role boundaries; Increased confidence; Perceived patient and therapist satisfaction; Research   There is a belief that manipulating their back is the only thing that can help, and then it’s quite difficult to introduce this biopsychosocial model because they kind of deny the presence of these psychosocial factors. (P13) Patient expectations, reflecting patient beliefs, were frequently cited as fuelling these difficulties. Some people want a quicker fix... for them their back is just another problem in their life and they want you as a therapist to deal with that, not them as a patient to deal with that. (P5) This heightened awareness of the role that beliefs of the patient play when implementing CFT provided participants with confidence to address those beliefs. In turn, many of the participants were not fazed by the limitation the negative beliefs posed, but instead were happy to address them. Sometimes they don’t want to hear what you have to say. They’ll just say ‘yeah, my disc is the problem, I just know I have a prolapse’... that’s a barrier that’s hard to move... but one I’m happy to start to change. (P2) Increased awareness of the importance of the therapeutic alliance. All participants in this study regarded a strong therapeutic alliance as an intrinsic ingredient for addressing cognitive, sychological and social factors. Well the relationship I think... I do believe that it creates a more open environment for the patient to feel heard... If they don’t feel there’s an alliance there, you can ask all the questions in the world but they won’t tell you anything. (P1) Participants described how an individualised approach to treatment aided in the development and maintenance of rapport, and how this facilitated a deeper insight into the individual cognitive and psychological drivers of pain for each patient. I think that individual interaction is highly important... rather than take them as being just another person with a low back pain problem... show that you are understanding of their viewpoint and individual pain... suss out what really makes them tick. Then I believe you make it work. (P10)  Theme 2: Self-reported changes in professional practice: Adoption of new screening tools; Several participants reported regularly using validated psycho-social screening tools after completing the CFT training. Tools used included the Orebro Musculoskeletal Pain Questionnaire and the STarTback Tool31 for the identification of individuals’ barriers to recovery. So we get a score from 0 to 10 on how depressed are you, how much fear avoidance do you have... It makes you think if you score between 7 and 10 on some of these questions, it’s of course relevant. (P6) For many participants, the results of these screening tools informed their route of questioning during patient interviews. I might use the question to explore a particular problem... so you can go ‘Well look you answered this in such a way, tell me a little bit more about it.’ (P1) Altered communication style; After completing the CFT training, participants reported a shift in their communication style from a rigid structured approach to an open and unrestrictive style. Participants identified how a open communication style promoted an easiness and fluidity in the exploration of the cognitive, psychological and social dimensions with patients. Now I think I’m much more open-ended, so I kind of ask open-ended questions like ‘What’s your story?’ you know, or ‘What brings you here?’ (P1) Adoption of a functional behavioural approach; The majority of participants described observing functional behaviours to gain insight into relevant cognitive and psychological factors (such as distress, anxiety, and fear avoidance) since completing CFT training. The observation of functional behaviours may vary between individuals, but will be likely to include targeting activities such as rolling in bed, sitting, standing up from sitting, walking, bending and lifting. Participants described this information as guiding the physiotherapy assessment process. Sometimes you get people in that don’t wear laces on shoes because they won’t bend down to their laces... and it gives you an idea of ‘Right, well, I better ask this person to do some bending and see what they look like.’... You have a look at how they move on a day-to-day basis as opposed to just from a clinical basis. (P1) Since completing CFT training, participants reported assessing and changing functional behaviours – such as assessing and changing bending or sitting, if reported as painful by the patient to increase the patient’s awareness of the relevance of cognitive and psychological factors in their pain experience. If you [as a patient] think your disc is vulnerable and you get extreme pain from forward bending, and within minutes you can actually move into a forward bending position pain free, most people would be ready to change that belief. (P6)  Theme 4: Increased confidence and satisfaction: Most participants described increased confidence in their ability to identify and address these factors in practice. I feel I’ve got enough grounding in research and training to say I feel completely confident in doing it. (P11) More specifically, many participants described how they were now confident to challenge the patient’s belief system, even if this led to some conflict. Now I’m more inclined to say ‘Listen, hold on a minute. Anyway I’ve just got to re-examine your point of view on this’ and that can sometimes lead to conflict... but I think you sometimes need conflict for conceptual change. (P1)  Role boundaries; Alongside this new understanding of their role, participants articulated an understanding of the limits or boundaries of their role. For example, participants acknowledged that addressing social factors (eg, workplace interventions) was an area that prompted participants to consider the boundaries of their role. I personally find interacting with people’s workplaces really tough, partly because I think there isn’t necessarily a relationship between me as a therapist and their workplace... We can’t give people a new job if they get fired because of their back pain. (P10) | 1.1 1.2 1.3 1.4 1.5 1.6 1.7 1.11 | Theme 3: Scope of practice Expanded role of the physiotherapist; Following the CFT training, participants described a clear understanding of how traditional hands-on approaches could be used in combination with newly developed skills to address cognitive, psychological and social factors. All participants commented on their ideal professional positioning as physiotherapists to combine their hands-on skills with the newly learned biopsychosocially orientated approach to successfully address the cognitive, psychological and social dimensions of pain. As physios we can put our hands on patients and assure them nothing is physically wrong... and with the training we can complement our hands-on and exercise expertise to treat things like anxiety. (P12) Role boundaries; Alongside this new understanding of their role, participants articulated an understanding of the limits or boundaries of their role. For example, participants acknowledged that addressing social factors (eg, workplace interventions) was an area that prompted participants to consider the boundaries of their role. I personally find interacting with people’s workplaces really tough, partly because I think there isn’t necessarily a relationship between me as a therapist and their workplace... We can’t give people a new job if they get fired because of their back pain. (P10) Additionally, all participants identified situations in which addressing certain psychological factors, particularly those associated with severe psychological trauma, were beyond their scope of practice. If someone has a post-traumatic stress disorder or had been abused, some of those instances are extremely depressing for 218 Synnott et al: Cognitive Functional Therapy training in back pain patients and may be beyond our professional boundaries to be managing... so I would refer onto someone with more specialised training. (P12) In considering their scope of practice in these situations, participants acknowledged these issues and their relevance for the patient’s pain, yet described their understanding that they were not appropriately qualified to treat these issues in practice. I can identify it, I can talk with the patient and help them consider the relevance of it to their pain, but when it is really traumatic for the patient then I am not capable enough of addressing thisproblem... It is because I am not trained in it. (P13) | 2.1 2.7 2.8 2.10 2.11 |  |  |  |  |
| Tilson 2014 | Multiple Encounter Course | participants completed four standardized assessments collated into one computer-based survey immediately before and after participating in the educational program. the 16-item EBP Beliefs Scale measures EBP attitudes and self-efficacy. the Evidence-based Practice Confidence (EPIC) Scale was used to assess self-efficacy for EBP [21]. EPIC consists of 11 items with responses ranging from 0 to 100% confidence (in 10 percentile increments). 13-item modified Fresno Test (mFT) which has demonstrated reliability and content and construct validity among physical therapists. Self-reported EBP behavior was assessed using the EBP Implementation Scale which has demonstrated construct and criterion validity among nurses  Qualitative assessment - Participants attended either a face-to-face semi-structured interview (1 participant:1 interviewer) or focus group (3-4 participants:1 interviewer) within 2 weeks of completing the PEAK program They were then facilitated to describe the impact of the program on their EBP attitudes, self- efficacy, knowledge, skills and practice behaviors. They were also asked to consider, from their professional experience, whether the program provided a benefit to patients. | Clinical Practice Research | The PEAK program was associated with a significant increase in self-efficacy for EBP (p < 0.001; Table 2). Mean EPIC (evidence informed practice confidence) scale scores improved from 65.3% to 82.9%.  Participants described integrating the research evidence more directly in patient care and in discussions with patients. “I find more that I’m incorporating it in my education of the patients. I’m always talking to them. This is why we’re doing this and so on. And I feel like they really appreciate that. Or I’ll use it to ask them, ‘these are the recommendations, what do you really prefer?’” (F5) | 1.5 1.6 |  |  |  |  | Participants described improved confidence for accurately and efficiently searching for the best available research evidence. They expressed new found confidence using a variety of tools and had increased expectations that their searches would yield useful information. “And I feel like I can better access the literature very efficiently with those patients. I'll do it on my phone or very, very quickly. And so I feel like I do that more because I'm more efficient. Whereas before I would go, oh, well, that might take me a little while.” (P5) Participants also reported new confidence making decisions about how to integrate research evidence into patient care. This seemed to come from a combination of personal success in identifying relevant research and from opportunities to discuss the integration of research evidence with their peers. Participants also described feeling more confident sharing study results with patients.  Participants also described developing their skills of critical appraisal as they expressed new insights into the strengths and weaknesses associated with different study designs. | 4.2 4.3 |
| Westervelt 2020 | Mentorship | Approximately 1 week before the initiation of the online mentoring sessions, both mentors and mentees completed a pre-participation survey. is survey was composed of questions about demographics, work environment, and beliefs and experiences regarding clinical mentoring. mentee survey also included the Clinician-Specific Outcome Scale (CSOS) and the Clinician Confidence Questionnaire for Patients with Spinal Pain (CCQPSP).   Six days after the completion of the first survey but before the initiation of the video-conferencing mentoring sessions, mentees completed a second preparticipation survey. is was used to provide a precursory, small- sample test-–retest reliability assessment of the outcome scales, consisting only of the CSOS and the CCQPSP  After completion of all mentoring sessions, both mentors and mentees completed a post-participation survey. is survey was composed of questions about their experience with this mentoring program. Mentees were again asked to complete the CSOS and the CCQPSP. For both the second preparticipation survey and the postparticipation survey, mentees were provided with the goals they had previously identified on the CSOS and asked only to rate them again. In order to assess the effect of the mentoring intervention on participants, the post-intervention administration of the CCQPSP and CSOS was compared with the first pre-intervention administration of the CCQPSP and CSOS via paired t test. All surveys were administered via LimeSurvey, a password-protected, secure survey tool.24 Participants were again emailed a closed hyperlink that was only accessible by its recipient. | Clinical Practice Leadership Education Research | Analysis also indicated that participants improved significantly in their ability to deal with the range of conditions seen in patients with spinal pain on their caseload. All goals set by participants on the CSOS also showed significant improvement (Table 3); however, the limited reliability previously shown for this measurement tool should be considered when interpreting these results Perspective: Defined by key phrases such as innovative ways of thinking, generation of new ideas, diverse communication outside work setting, and collaboration: "one thing that is important about mentorship, is getting you to think in a way that you would not normally think, and that was something I definitely appreciated" Perceived clinical benefits: Defined as clinicians’ belief that this study improved their confidence and clinical decision making: "The communication was great ... you can develop the clinical decision making over an internet connection just as well as you can in person and I thought it was ... everything I needed" Additionally, participants reported that hearing diverse perspectives was both confirmatory and confidence building as well as an opportunity to integrate new ideas and ways of thinking, this opportunity for new perspective and connection directly relates to the collaboration component of the APTA’s vision statement. 5 of 8 mentees reported that gaining new ideas for treatment was one of the main benefits they received from the mentoring intervention. One of the strongest themes that emerged during qualitative analysis of the mentee’s data was the perceived clinical benefits of the mentoring model. e clinical benefits most strongly and frequently reported by the mentees included increased confidence, improved clinical decision-making and reasoning skills, and confirmation of practice patterns.  These qualitative findings were supported by survey results, in which 6 mentees reported increased confidence as one of the main benefits received from the intervention. the theme of perceived clinical benefits also encompassed improved clinical decision making and reasoning | 1.3 1.5 1.6 1.7 1.9 1.11 | Reflection: Defined as the opportunity for contemplation about self and practice and return to foundational knowledge "It helped me realize what some of those personal biases are that I started following instead of versus what’s kind of been researched and demonstrated to work well" Mentees frequently noted that this mentoring model allowed them the opportunity to dedicate time for reflection and expressed the rarity of this time, Mentees emphasized the importance of reflecting on their own practice stating, “if you start to get complacent and think you know everything, then you start getting into trouble” | 2.3 2.6 | Reflection: Defined as the opportunity for contemplation about self and practice and return to foundational knowledge "It helped me realize what some of those personal biases are that I started following instead of versus what’s kind of been researched and demonstrated to work well" Mentees frequently noted that this mentoring model allowed them the opportunity to dedicate time for reflection and expressed the rarity of this time,  Mentees emphasized the importance of reflecting on their own practice stating, “if you start to get com- placent and think you know everything, then you start getting into trouble”   Survey data revealed that 5 mentees reported an increased interest in postprofessional education after reflecting upon their participation in this intervention, potentially indicating a desire for more opportunities to build upon the foundations of their clinical practice. Furthermore, combination with survey data indicated that mentee participants improved significantly on their perception of their ability to evaluate their treatments. | 3.1 3.2 | Reflection: Defined as the opportunity for contemplation about self and practice and return to foundational knowledge "It helped me realize what some of those personal biases are that I started following instead of versus what’s kind of been researched and demonstrated to work well" | 4.2 |
| Whitman 2020 | Residency and Fellowship | [Survey For the current study, we specifically created an online survey based on studies evaluating outcomes of clinical residency programs [10,12] and on expert recommendation [7]. The survey included questions about the impact of FT on professional attributes and skills, self-perception of professionalism, scholarly activities, and professional involvement. Participants also reported on the impact of training on salaries and annual gross income. Survey items asking about the impact of FT on professional attributes, skills, self-perception, and income were assessed on an 11-point Likert-like scale, where −5 was significant negative influence, 0 was no influence, and 5 was significant positive influence. We also assessed access to FT, including access to Fellow of the American Academy of Orthopedic Manual Physical Therapists (AAOMPT)-credentialed mentors and in-residence OMPT programs and whether the graduate would have pursued FT without access to a hybrid-model program. Optional open-ended questions asked how training impacted graduates professionally and personally. They also asked about perceived barriers to FT and about life situations and responsibilities that had to be balanced alongside the program.](http://www.surveymonkey.com/) | Clinical Practice Leadership Education Research | Mean scores for survey items related to perceived impact on professionalism, application of knowledge, and impact of FT on patient outcomes ranged from 3.7 to 4.7 points  Mean scores for impact on clinical reasoning skills in all phases of the care process ranged from 3.9 to 4.7 points (Table 4). Impact on technical skills varied from 3.6 to 4.4 points,  Metacognition and clinical skills were identified as characteristics of expertise. Graduates identified FT as having a positive impact on clinical reasoning, including both critical thinking and decision-making, and on confidence with teaching.  Graduates indicated improved communication skills in their professional and personal lives.  Enhanced humility, commitment, confidence, and life-long learning skills were traits and values identified as positively impacting graduates professionally and personally. | Qualitized: PTs reported that there was a positive impact of FT on professional attributes and skills, self-perception of professionalism, confidence, patient outcomes, clinical reasoning, communication and metacognition.  1.1 1.5 1.6 1.11 | The highest mean (SD) scores were reported for viewing oneself as a professional (4.7 [0.7] points)  Almost all graduates were members of the American Physical Therapy Association (APTA) (72/75, 96.0%) and the AAOMPT (73/75, 97.3%), and 22.7% (17/75) had held official positions in these organizations (board or committee member) (Table 5). Additionally, 24.0% (18/75) had served in their state chapter or district as a state delegate, board member, or committee member.   For professionalism and collaboration, graduates indicated they had a broader view of the profession after training and that they had developed a network of colleagues to consult and collaborate with. Graduates indicated improved communication skills in their professional and personal lives. Enhanced humility, commitment, confidence, and life-long learning skills were traits and values identified as positively impacting graduates professionally and personally. | 2.2 2.5 2.7 | Mean (SD) impact on confidence in mentoring ranged from 3.4 (3.2) points for mentoring fellows-in-training to 4.3 (2.0) points for mentoring peers in the clinic or community.  Respondents were involved as members and leaders of professional associations and in scholarly work and educational activities  Graduate involvement in education was a common finding in this study. After FT, 42.7% (32/75) were lead instructors in entry-level postprofessional PT programs, and 53.3% (40/75) were lead instructors for continuing education seminars (Table 6). About half had developed curricular content for entry-level (40.0%, 30/75) or postprofessional PT education online and onsite lab courses (57.3%, 44/75). Clinical mentorship was a reported for many; 89.3% (67/75) reported involvement as mentors for peers in clinical practice, entry-level DPT students (from 65 programs), residents (from 23 credentialed programs), or fellows-in-training (from 9 credentialed programs) (Table 5). Most (53/75, 70%) reported that FT positively influenced their confidence in teaching and mentoring.  having a positive impact on clinical reasoning, including both critical thinking and decision-making, and on confidence with teaching. and life-long learning skills were traits and values identified as positively impacting graduates professionally and personally. | 3.1 3.2 3.8 | Respondents were involved as members and leaders of professional associations and in scholarly work and educational activities.   Most (57/75, 76.0%) were involved in research during fellowship or since graduation (Table 6). More than a quarter (21/75, 28.0%) had published in peer-reviewed journals and professional texts, and conducted professional platform presentations (24/75, 32.0%), presented posters (23/75, 30.7%), or were invited speakers at national (15/75, 20.0%) and district or state level (28/75, 37.3%) professional meetings. | 4.1 4.8 |
| Williams 2019 | Mentorship | Patient level outcomes. The primary outcome measure was the proportion of patients achieving clinically significant improvements on the Patient Specific Functional Scale (PSFS), a patient-specific tool for measuring health-related quality of life (HRQL). These outcomes were collected in collaboration with the patient’s physiotherapist (which is the advised method for use of the tool [73]) and entered on to the patient’s question- naire containing the other outcome measures. Clinical significance was set at a level using established data for the minimal clinically important difference (MCID) being reported to be 3 points for each single identified activity and 2 points for aggregate activities. The proportion of patients achieving clinically significant outcomes (i.e. achieving the level for MCID) was calculated.  The secondary outcome measures at patient-level were EQ-5D-5L, the Patient Activation Measure and the MedRisk instrument. The EQ-5D-5L is a two-section generic measure of HRQL [74], the descriptive section yielding a 5 digit health state profile which was then con- verted into a single index value and the visual analogue scale giving respondent’s self-rated health on a scale of 0–100; higher scores in both sections indicate higher levels indices of health.   The Patient Activation Measure (PAM) is a measurement of the knowledge, skills and confidence a person has in managing their own health and healthcare [68], delivering a patient activation score of between 0 and 100, higher scores indicating greater levels of activation.   The MedRisk instrument for Measuring Patient Satisfaction with Physical Therapy Care (MRPS) is a measure of patient satisfaction in the musculoskeletal physiotherapy context [72], mean scores being used to assess satisfaction from 1.0 (highly dissatisfied) to 5.0 (highly satisfied).  Physiotherapist level outcomes. Physiotherapist performance was assessed by each physiotherapist being observed with a new patient and a follow-up patient and then discussing their practice. This was performed by an assessor who was blinded (to whether the participant had received the intervention), independent (from outside the organisation) and experienced (familiar with using the performance criteria). Two physiotherapist-level outcomes were used; both derived from the assessment of Master’s level post-graduate student performance in the musculoskeletal context [32]. One outcome was the score and the other outcome was the score divided into 10% bands (e.g. 40–49%; 50–59%). The specific tool selected for measurement of performance was selected on the basis of its supporting empirical data where the construct of the assessment tool was supported by triangulation of data from multiple methods and studies | Clinical Practice Leadership | Primary outcome measure Using the established data for the minimal clinically important difference (MCID) of 3 points for each single identified activity and 2 points for aggregate activities, the proportion of patients achieving the level for MDC and MCID was 80.0% of the patients in the intervention arm compared with 63.8% of patients in the control arm (Fig 4). Using logistic regression analysis, in the minimally adjusted model the difference in the proportion of patients achieving the MCID between the intervention and control arm gives an odds ratio of 4.14 and a p value of 0.009. In the fully adjusted model, the odds ratio strengthens to 4.24 with a p value of 0.023  Secondary outcome measures Physiotherapist performance. The physiotherapists’ performances improved as can be seen in Fig 5. Fig 5A shows the mean score of the physiotherapists’ performance as 47.8 prior to the intervention, and 55.1 post intervention (post I). A subsequent measure of performance post-intervention shows a further mean increase to 56.9 (post II). Fig 5B captures the clinical and educational relevance of these score changes. Displaying performance by banding, 75% of physiotherapists prior to intensive mentoring would have been below the mark of 50, which is the pass mark for Masters level performance on the University assessment criteria. This reduces to only 6.7% of physiotherapists post-intervention, with 53.3% of physiotherapists achieving a score in a higher band of between 60 and 70, which none achieved prior to the intervention.  Facilitating physiotherapists’ clinical reasoning through work-based mentoring significantly increases the proportion of their patients achieving clinically significant patient-specific HRQL outcomes. While the statistical evidence demonstrated is important, the increase from just under 2 in 3 patients attending the physiotherapy service achieving the MCID on PSFS to 4 in 5 patients achieving this is a welcome increase for physiotherapists, referring clini- cians, managers and not least of all, patients. This is especially important given that this out- come measure is set for the functional activities that patients themselves have identified as being most affected by their musculoskeletal condition and therefore the most relevant to address in the rehabilitation process. | Qualitized: Patients treated by PTS that underwent the mentorship had clinically significant improvement compared to before engaging in the mentorship and PTs performed significantly better on an outcome measure derived from the assessment of Master’s level post-graduate student performance in the musculoskeletal contex after engaging in the mentorship.   1.5 1.6 1.11 | Facilitating physiotherapists’ clinical reasoning through work-based mentoring significantly increases the proportion of their patients achieving clinically significant patient-specific HRQL outcomes. While the statistical evidence demonstrated is important, the increase from just under 2 in 3 patients attending the physiotherapy service achieving the MCID on PSFS to 4 in 5 patients achieving this is a welcome increase for physiotherapists, referring clini-cians, managers and not least of all, patients. This is especially important given that this outcome measure is set for the functional activities that patients themselves have identified as being most affected by their musculoskeletal condition and therefore the most relevant to address in the rehabilitation process. | 2.7 |  |  |  |  |
