# Supplementary material for: The educational pathway to Advanced Practice for the physiotherapist: A systematic mixed studies review
Source: PLoS One. 2025 May 12;20(5):e0322626. doi: 10.1371/journal.pone.0322626 (PMC12068731; doi:10.1371/journal.pone.0322626)
Supplement: S5 Table — (DOCX) [file pone.0322626.s005.docx]

| **CERQual Qualitative Evidence Profile** | | | | | | |  |  |
| --- | --- | --- | --- | --- | --- | --- | --- | --- |
|  | **Review Finding** | **Studies** | **ML** | **Relevance** | **Coherence** | **Adequacy** | **Overall CERQual Ax of Confidence** | **Explanation of Judgement** |
| Textual descriptions of post-licensure educational pathways that are distinct from one another and engaged in by PTs to advance their level of practice (see findings section) | Masters Level Education | 1-9 | No or very minor concerns | No or very minor concerns | No or very minor concerns | Minor Concerns | High Confidence | Minor concerns in 1/4 domains re adequacy of data, 3/9 studies contributing to this review finding had low |
|  | Residency and Fellowship Programs | 10-25 | No or very minor concerns | No or very minor concerns | No or very minor concerns | No or very minor concerns | High Confidence | No or very minor concerns in all four domains, with 16 studies contributing to this review finding. |
|  | Accredited Area of Practice Education | 26-29 | Moderate Concerns | Minor Concerns | No or very minor concerns | Minor Concerns | Low Confidence | Moderate concerns re methodological limitations, 2/4 studies had combined moderate to low ratings on the QUADs, as well as minor concerns re low total number (4) studies contributing to the finding |
|  | Mentorship | 30-33 | No or very minor concerns | No or very minor concerns | No or very minor concerns | Minor Concerns | High Confidence | Only minor concerns in 1/4 domains re adequacy of data due to a low total number (4) of studies contributing to the review findings. |
|  | Multiple Encounter Courses | 34-55 | Minor Concerns | Minor Concerns | No or very minor concerns | Minor Concerns | Moderate Confidence | Minor concerns in 3/4 domains due to combined low to moderate ratings on components of the QUADs relating to these domains, with 22 studies contributing to this review finding. |
|  | Single Encounter Courses | 56-81 | Minor Concerns | Minor Concerns | No or very minor concerns | Minor Concerns | Moderate Confidence | Minor concerns in 3/4 domains due to combined low to moderate ratings on components of the QUADs relating to these domains, with 26 studies contributing to this review finding. |
|  |  |  |  |  |  |  |  |  |
| Pillars of Advanced Practice demonstrated by PTs after traversing each educational pathway | **Masters Level Education:** Consistent demonstration (9 of 9 studies) of all four pillars. Frequency of individual competency demonstration overall ranged moderate to high. | 1-9 | No or very minor concerns | No or very minor concerns | No or very minor concerns | No or very minor concerns | High Confidence | No or very minor concerns in all four domains, with 9 studies contributing to this review finding. |
|  | **Residency and Fellowship Programs:** Inconsistent demonstration (4 of 16 studies) of all four pillars. Frequency of individual competency demonstration overall ranged low to high. | 10-25 | No or very minor concerns | No or very minor concerns | No or very minor concerns | Minor Concerns | High Confidence | Minor concerns in 1/4 domains, with 16 studies contributing to this review finding, |
|  | **Accredited Area of Practice Education:** no demonstration (0 of 4 studies) of all four pillars. Frequency of individual competency demonstration overall was low. | 26-29 | Moderate Concerns | Minor Concerns | No or very minor concerns | Minor Concerns | Low Confidence | Moderate concerns re methodological limitations, 2/4 studies had combined moderate to low ratings on the QUADs, as well as minor concerns re low total number (4) studies contributing to the finding |
|  | **Mentorship:** somewhat consistent demonstration (2 of 4 studies) of all four pillars. Frequency of individual competency demonstration overall ranged low to moderate. | 30-33 | No or very minor concerns | No or very minor concerns | No or very minor concerns | Minor Concerns | High Confidence | Minor concerns in 1/4 domains re adequacy of data due to a low total number (4) of studies contributing to the review findings. |
|  | **Multiple Encounter Courses:** no demonstration (0 of 22 studies) of all four pillars. Frequency of individual competency demonstration overall ranged low to moderate. | 34-55 | No or very minor concerns | Minor Concerns | No or very minor concerns | Minor Concerns | Moderate Confidence | Minor concerns in 3/4 domains due to combined low to moderate ratings on components of the QUADs relating to these domains, with 22 studies contributing to this review finding. |
|  | **Single Encounter Courses:** no demonstration (0 of 26 studies) of all four pillars. Frequency of individual competency demonstration overall ranged low to moderate | 56-81 | Minor Concerns | Minor Concerns | No or very minor concerns | Minor Concerns | Moderate Confidence | Minor concerns in 3/4 domains due to combined low to moderate ratings on components of the QUADs relating to these domains, with 26 studies contributing to this review finding. |
