# Supplementary material for: The educational pathway to Advanced Practice for the physiotherapist: A systematic mixed studies review
Source: PLoS One. 2025 May 12;20(5):e0322626. doi: 10.1371/journal.pone.0322626 (PMC12068731; doi:10.1371/journal.pone.0322626)
Supplement: S6 Table — (DOCX) [file pone.0322626.s006.docx]

**Multiprofessional Framework for Advanced Practice in England. Health Education England; 2017.**

| Clinical Practice |
| --- |
| 1.1 Practice in compliance with their respective code of professional conduct and within their scope of practice, being responsible and accountable for their decisions, actions and omissions at this level of practice. |
| 1.2 Demonstrate a critical understanding of their broadened level of responsibility and autonomy and the limits of own competence and professional scope of practice, including when working with complexity, risk, uncertainty and incomplete information. |
| 1.3 Act on professional judgement about when to seek help, demonstrating critical reflection on own practice, self-awareness, emotional intelligence, and openness to change. |
| 1.4 Work in partnership with individuals, families and carers, using a range of assessment methods as appropriate (e.g. of history-taking; holistic assessment; identifying risk factors; mental health assessments; requesting, undertaking and/or interpreting diagnostic tests; and conducting health needs assessments). |
| 1.5 Demonstrate effective communication skills, supporting people in making decisions, planning care or seeking to make positive changes, using Health Education England’s framework to promote person-centred approaches in health and care |
| 1.6 Use expertise and decision-making skills to inform clinical reasoning approaches when dealing with differentiated and undifferentiated individual presentations and complex situations, synthesizing information from multiple sources to make appropriate, evidence-based judgements and/or diagnoses. |
| 1.7 Initiate, evaluate and modify a range of interventions which may include prescribing medicines, therapies, life style advice and care. |
| 1.8 Exercise professional judgement to manage risk appropriately, especially where there may be complex and unpredictable events and supporting teams to do likewise to ensure safety of individuals, families and carers. |
| 1.9 Work collaboratively with an appropriate range of multi-agency and interprofessional resources, developing, maintaining and evaluating links to manage risk and issues across organizations and settings. |
| 1.10 Act as a clinical role model/advocate for developing and delivering care that is responsive to changing requirements, informed by an understanding of local population health needs, agencies and networks. |
| 1.11 Evidence the underpinning subject-specific competencies i.e. knowledge, skills and behaviours relevant to the role setting and scope, and demonstrate application of the capabilities to these, in an approach that is appropriate to the individual role, setting and scope. |

| Leadership and Management |
| --- |
| 2.1 Pro-actively initiate and develop effective relationships, fostering clarity of roles within teams, to encourage productive working. |
| 2.2 Role model the values of their organization/place of work, demonstrating a personcentred approach to service delivery and development. |
| 2.3 Evaluate own practice, and participate in multi-disciplinary service and team evaluation, demonstrating the impact of advanced clinical practice on service function and effectiveness, and quality (i.e. outcomes of care, experience and safety). |
| 2.4 Actively engage in peer review to inform own and other’s practice, formulating and implementing strategies to act on learning and make improvements. |
| 2.5 Lead new practice and service redesign solutions in response to feedback, evaluation and need, working across boundaries and broadening sphere of influence. |
| 2.6 Actively seek feedback and involvement from individuals, families, carers, communities and colleagues in the co-production of service improvements. |
| 2.7 Critically apply advanced clinical expertise in appropriate faciliatory ways to provide consultancy across professional and service boundaries, influencing clinical practice to enhance quality, reduce unwarranted variation and promote the sharing and adoption of best practice. |
| 2.8 Demonstrate team leadership, resilience and determination, managing situations that are unfamiliar, complex or unpredictable and seeking to build confidence in others. |
| 2.9 Continually develop practice in response to changing population health need, engaging in horizon scanning for future developments (e.g. impacts of genomics, new treatments and changing social challenges). |
| 2.10 Demonstrate receptiveness to challenge and preparedness to constructively challenge others, escalating concerns that affect individuals’, families’, carers’, communities’ and colleagues’ safety and well-being when necessary. |
| 2.11 Negotiate an individual scope of practice within legal, ethical, professional and organizational policies, governance and procedures, with a focus on managing risk and upholding safety. |

| Education |
| --- |
| 3.1 Critically assess and address own learning needs, negotiating a personal development plan that reflects the breadth of ongoing professional development across the four pillars of advanced clinical practice. |
| 3.2 Engage in self-directed learning, critically reflecting to maximize clinical skills and knowledge, as well as own potential to lead and develop both care and services. |
| 3.3 Engage with, appraise and respond to individuals’ motivation, development stage and capacity, working collaboratively to support health literacy and empower individuals to participate in decisions about their care and to maximize their health and well-being. |
| 3.4 Advocate for and contribute to a culture of organizational learning to inspire future and existing staff. |
| 3.5 Facilitate collaboration of the wider team and support peer review processes to identify individual and team learning. |
| 3.6 Identify further developmental needs for the individual and the wider team and supporting them to address these. |
| 3.7 Supporting the wider team to build capacity and capability through work-based and inter-professional learning, and the application of learning to practice |
| 3.8 Act as a role model, educator, supervisor, coach and mentor, seeking to instill and develop the confidence of others. |

| Research |
| --- |
| 4.1 Critically engage in research activity, adhering to good research practice guidance, so that evidence-based strategies are developed and applied to enhance quality, safety, productivity and value for money |
| 4.2 Evaluate and audit own and others’ clinical practice, selecting and applying valid, reliable methods, then acting on the findings. |
| 4.3 Critically appraise and synthesize the outcome of relevant research, evaluation and audit, using the results to underpin own practice and to inform that of others. |
| 4.4 Take a critical approach to identify gaps in the evidence base and its application to practice, alerting appropriate individuals and organizations to these and how they might be addressed in a safe and pragmatic way. |
| 4.5 Actively identify potential need for further research to strengthen evidence for best practice. This may involve acting as an educator, leader, innovator and contributor to research activity and/or seeking out and applying for research funding. |
| 4.6 Develop and implement robust governance systems and systematic documentation processes, keeping the need for modifications under critical review. |
| 4.7 Disseminate best practice research findings and quality improvement projects through appropriate media and fora (e.g. presentations and peer review research publications). |
| 4.8 Facilitate collaborative links between clinical practice and research through proactive engagement, networking with academic, clinical and other active researchers. |
