# Supplementary material for: The educational pathway to Advanced Practice for the physiotherapist: A systematic mixed studies review
Source: PLoS One. 2025 May 12;20(5):e0322626. doi: 10.1371/journal.pone.0322626 (PMC12068731; doi:10.1371/journal.pone.0322626)
Supplement: S7 Table — (DOCX) [file pone.0322626.s007.docx]

|  | | | |  |  |
| --- | --- | --- | --- | --- | --- |
| **Title** | **Authors** | **Yr** | **Journal** | **In- or Excluded** | **Exclusion Justification** |
| **Influence of a Post-Graduate Physical Therapy Residency Program on Clinical Reasoning, Professional Development, and Career Advancement in Nairobi, Kenya** | Cunningham, Shala | 2018 | ProQuest Dissertations and Theses | Excluded | Exclusion reason: Not primary research |
| **Relatório de estágio em terapia manual ortopédica** | da Costa Marques, Rui Emanuel Ferreira | 2010 | PQDT - Global | Excluded | Exclusion reason: Wrong outcomes; |
| **Continuing professional development for physiotherapists: exploring their choices in career long learning** | Johnson, Helena Claire | 2008 | PQDT - UK & Ireland | Excluded | Exclusion reason: Wrong outcomes; |
| **Portfolios in practice: Developing advancing practice within a musculoskeletal competency-based model.** | Langridge, N; Welch, H; Jones, D; Small, C; Lynch, G; Ganatra, B | 2023 | Musculoskeletal science & practice | Excluded | Exclusion reason: Not primary research; |
| **Exploration of mentor and mentee perspectives of a mentored clinical practice programme to improve patient outcomes in musculoskeletal physiotherapy.** | Williams, Aled; Phillips, Ceri J; Rushton, Alison | 2022 | PloS one | Excluded | Exclusion reason: Wrong outcomes; |
| **Accreditation of advanced clinical practice of musculoskeletal physiotherapy in England: a qualitative two-phase study to inform implementation.** | Noblet, Timothy; Heneghan, Nicola R; Hindle, Jacqueline; Rushton, Alison | 2021 | Physiotherapy | Excluded | Exclusion reason: Wrong intervention; |
| **[Impact of a continuous education program on the quality of assistance offered by intensive care physiotherapy].** | Pinto, Walkyria Araujo Macedo; Rossetti, Heloisa Baccaro; Araujo, Abigail; Sposito, Jose Jonas Jr; Salomao, Hellen; Mattos, Simone Siqueira; Rabelo, Melina Vieira; Machado, Flavia Ribeiro | 2014 | Revista Brasileira de terapia intensiva | Excluded | Exclusion reason: Wrong intervention; |
| **Evaluation of perceived collaborative behaviour amongst stakeholders and clinicians of a continuing education programme in arthritis care.** | Lundon, Katie; Kennedy, Carol; Rozmovits, Linda; Sinclair, Lynne; Shupak, Rachel; Warmington, Kelly; Passalent, Laura; Brooks, Sydney; Schneider, Rayfel; Soever, Leslie | 2013 | Journal of interprofessional care | Excluded | Exclusion reason: Population is not exclusively PT; |
| **Shouldering the Burden of Evidence-Based Practice: The Experiences of Physiotherapists Partaking in a Community of Practice.** | McCreesh, Karen; Larkin, Louise; Lewis, Jeremy | 2016 | Rehabilitation research and practice | Excluded | Exclusion reason: Wrong intervention; |
| **Impact and feasibility of the Allied Health Professional Enhancement Program placements - experiences from rural and remote Queensland.** | Martin, Priya; Kumar, Saravana; Stone, Melinda; Abernathy, LuJuana; Burge, Vanessa; Lizarondo, Lucylynn | 2016 | Advances in medical education and practice | Excluded | Exclusion reason: Population is not exclusively PT; |
| **Continuing professional development is associated with increasing physical therapists' roles in arthritis management in Canada and the Netherlands.** | Li, Linda C; Hurkmans, Emalie J; Sayre, Eric C; Vliet Vlieland, Thea P M | 2010 | Physical therapy | Excluded | Exclusion reason: Wrong intervention; |
| **Factors that influence the clinical decision-making of rehabilitation professionals in long-term care settings.** | Wainwright, Susan Flannery; McGinnis, Patricia Quinn | 2009 | Journal of allied health | Excluded | Exclusion reason: Population is not exclusively PT; |
| **Continuing Professional Development of physiotherapists based in community primary care trusts: a qualitative study investigating perceptions, experiences and outcomes.** | Gunn, Hilary; Goding, Lois | 2009 | Physiotherapy | Excluded | Exclusion reason: Wrong intervention; |
| **Using clinical outcomes to explore the theory of expert practice in physical therapy.** | Resnik, Linda; Jensen, Gail M | 2003 | Physical therapy | Excluded | Exclusion reason: Wrong intervention; |
| **A programme for developing clinical reasoning skills in graduate physiotherapists.** | Higgs, J | 1993 | Medical teacher | Excluded | Exclusion reason: Wrong outcomes; |
| **Clinical education model for staff training in orthopedic manual therapy.** | Yamada, J K; Montague, E C | 1984 | Physical therapy | Excluded | Exclusion reason: Not primary research |
| **Assessing the change of practice by physical therapists after a continuing education program.** | Mays, M J | 1984 | Physical therapy | Excluded | Exclusion reason: Wrong outcomes; |
| **The 2022 Massive Open Online Course (MOOC) to train physiotherapists in the management of people with spinal cord injuries: a qualitative and quantitative analysis of learners' experiences and its impact** | Glinsky J.V.; Ilha J.; Xiong Y.; Gomez G.; Rostagnor S.; Martin-Manjarres S.; Tranter K.; Muldoon S.; Weerts E.; Harvey L.A. | 2023 | Spinal Cord | Excluded | Exclusion reason: Population is not exclusively PT; |
| **Evaluating a Muscle Ultrasound Education Program: Theoretical Knowledge, Hands-on Skills, Reliability, and Satisfaction of Critical Care Physiotherapists** | Gonzalez-Seguel F.; Pinto-Concha J.J.; Rios-Castro F.; Silva-Gutierrez A.; Camus-Molina A.; Mayer K.P.; Parry S.M. | 2021 | Archives of Rehabilitation Research and Clinical Translation | Excluded | Exclusion reason: Wrong outcomes; |
| **Effectiveness of a virtual physiotherapy for Parkinson's course using a mixed on-demand and live model: Lessons learned from Malta** | Clark V.; Domingos J.; Masalha V. | 2023 | Journal of Parkinson's Disease | Excluded | Exclusion reason: Not primary research |
| **High-Fidelity Emergency On-Call Patient Simulation in Postgraduate Physiotherapy Education** | Shetty S.; Hazleton C.; Willingham W.; Jones H.; Malhan N. | 2022 | Resuscitation | Excluded | Exclusion reason: Not primary research; |
| **Light-touch mentorship of physiotherapists in face to face and digital consultations supports development of clinical expertise** | McLean L.; Johnson W. | 2022 | Physiotherapy (United Kingdom) | Excluded | Exclusion reason: Not primary research; |
| **Clinical effectiveness of a physiotherapy work-based mentoring clinical reasoning intervention: a stepped wedge cluster randomised controlled trial** | Williams A.; Rushton A.; Lewis J.; Phillips C. | 2020 | Physiotherapy (United Kingdom) | Excluded | Exclusion reason: Not primary research; |
| **Physical therapists' experiences of learning and delivering a complex behavioral medicine intervention to adolescents with pain** | Frygner-Holm S.; Asenlof P.; Ljungman G.; Soderlund A. | 2021 | Physiotherapy theory and practice | Excluded | Exclusion reason: Wrong outcomes |
| **The efficacy of advanced practice physiotherapy assessment for cervical and lumbar spine pathologies** | Motyka E.; Banaszek D.; Inglis T.; Street J. | 2019 | CMAJ. Canadian Medical Association Journal | Excluded | Exclusion reason: Not primary research; |
| **Advancing physiotherapy competence in dementia care.a service development initiative** | Jones C. | 2019 | Age and Ageing | Excluded | Exclusion reason: Not primary research; |
| **Pilot of an advanced practitioner physiotherapist to improve the triage process and assess patients with non-inflammatory conditions in rheumatology** | Fish S. | 2019 | Physiotherapy (United Kingdom) | Excluded | Exclusion reason: Not primary research; |
| **Developing an evidence-based Making Every Contact Count (MECC) model of practice within MSK physiotherapy services** | Moss C.; Bancroft D. | 2019 | Physiotherapy (United Kingdom) | Excluded | Exclusion reason: Not primary research; |
| **Using simulation to enhance the skills of paediatric cardiorespiratory physiotherapists: A collaborative approach** | Broughton E.; Akers E.; Mercer H.; Wright S.; Balls J.; Griffin S. | 2018 | Archives of Disease in Childhood | Excluded | Exclusion reason: Not primary research; Kaitlyn Maddigan |
| **Changes in physical therapist attitudes, beliefs, and confidence about low back pain management following participation in a Psychologically Informed Physical Therapy (PIPT) training course** | Beneciuk J.; George S.; Greco C.; Schneider M.; Wegener S.; Delitto A. | 2017 | Journal of Pain | Excluded | Exclusion reason: Not primary research; |
| **Physiotalk: connectedness and constructive change-a qualitative study** | Thomas J.; McVey N.; Bulley C. | 2017 | European Journal of Physiotherapy | Excluded | Exclusion reason: Not primary research; |
| **Evidence-based Practice Intentions and Long-term Behaviours of Physiotherapy Graduates Following an Intensive Education Programme** | Perraton L.; Machotka Z.; Gibbs C.; Mahar C.; Kennedy K.; Grimmer K. | 2017 | Physiotherapy research international : the journal for researchers and clinicians in physical therapy | Excluded | Exclusion reason: Population is not exclusively PT |
| **Embedding Evidence-based Practice Education into a Post-graduate Physiotherapy Program: Eight Years of pre-Post Course Evaluations** | Perraton L.; Machotka Z.; Grimmer K.; Gibbs C.; Mahar C.; Kennedy K. | 2017 | Physiotherapy research international : the journal for researchers and clinicians in physical therapy | Excluded | Exclusion reason: Entry Level Education; |
| **Creating a learning culture for developing clinical reasoning through postgraduate manipulative physiotherapy education** | Madi M.; Griffiths M.; Rushton A.; Heneghan N. | 2016 | Manual Therapy | Excluded | Exclusion reason: Not primary research; |
| **Graduating residents' perspectives of the influence of orthopedic residency training on professional development in Nairobi, Kenya** | Cunningham S.; McFelea J. | 2016 | Manual Therapy | Excluded | Exclusion reason: Not primary research; |
| **Peer assessment as an effective strategy for implementing a physical therapy low back pain guideline** | Van Dulmen S.A.; Maas M.; Staal J.B.; Rutten G.; Kiers H.; Nijhuis-Van Der Sanden M.W.G.; Van Der Wees P.J. | 2015 | Physiotherapy (United Kingdom) | Excluded | Exclusion reason: Not primary research; |
| **Promoting clinical reasoning using the international classification of function, disability and health (ICF) framework for continuing education development in Rwanda** | Sander A.; Urimubenshi G.; Chevan J.; Mann M.; Dunleavy K. | 2015 | Physiotherapy (United Kingdom) | Excluded | Exclusion reason: Not primary research; |
| **Fiseo, spot and ispot-full immersion simulation workshops for post graduate physiotherapy learning in acute care** | Thomas A.J.; Gill R. | 2015 | Physiotherapy (United Kingdom) | Excluded | Exclusion reason: Not primary research; |
| **Supporting physiotherapy professional development through skill assessment within a mentoring process: An experience in Nepal** | Pokhrel S.; Retis C. | 2015 | Physiotherapy (United Kingdom) | Excluded | Exclusion reason: Not primary research; |
| **A project expanding the scope of practice for physiotherapists in the emergency department: A lead site's report** | Harding P.A.; Mathew J. | 2015 | Physiotherapy (United Kingdom) | Excluded | Exclusion reason: Not primary research; |
| **Collegial mentoring through routine patient consultation-a new learning strategy in outpatient physical therapy settings** | Gottlieb D.; Ariel I.; Deutscher D.; Moreno S. | 2015 | Physiotherapy (United Kingdom) | Excluded | Exclusion reason: Not primary research; |
| **PT graduates' perceptions of a higher education early intervention training program on employment and practice** | Golub-Victor A.C.; Dumas H. | 2015 | Pediatric physical therapy : the official publication of the Section on Pediatrics of the American Physical Therapy Association | Excluded | Exclusion reason: Entry Level Education; |
| **Effective communication and teaching skills training equips physiotherapy mentors' for their educator roles** | Soh T.S.; Loy F.L.; Teo S.Y.; Shen L.J.M. | 2014 | Annals of the Academy of Medicine Singapore | Excluded | Exclusion reason: Not primary research; |
| **Learning and using behavior change techniques to promote physical activity in rheumatoid arthritis** | Demmelmaier I. | 2014 | Annals of the Rheumatic Diseases | Excluded | Exclusion reason: Not primary research; |
| **An evaluation of health-related quality of life of patients aroused from prolonged coma when treated by physiotherapists with or without training in the 'academy of life' programme** | Tomaszewski W.; Manko G.; Ziolkowski A.; Pachalska M. | 2013 | Annals of Agricultural and Environmental Medicine | Excluded | Exclusion reason: Wrong Population; |
| **Patient satisfaction with ACPAC program trained extended role practitioners: A multi-centre study** | Warmington K.; Kennedy C.; Lineker S.; Soever L.; Passalent L.; Lundon K.; Shupak R.; Schneider R. | 2012 | Journal of Rheumatology | Excluded | Exclusion reason: Population is not exclusively PT; |
| **Testing the sports physiotherapy competency 'rehabilitation' in formal learning in Bulgaria** | Tasheva R.; Donaghy M.; Gigova V. | 2011 | Physiotherapy (United Kingdom) | Excluded | Exclusion reason: Not primary research; |
| **Master program in neurological physiotherapy-a way to promote development of both academic knowledge and advanced clinical skills** | Normann B. | 2011 | Physiotherapy (United Kingdom) | Excluded | Exclusion reason: Not primary research; |
| **Spanning boundaries in an online continuing education course to support evidence based physiotherapy practice in manual therapy across social networks** | Evans C.; Yeung E.; Guilcher S. | 2011 | Physiotherapy (United Kingdom) | Excluded | Exclusion reason: Not primary research; |
| **Advanced clinician practitioner in arthritis care (ACPAC) program-trained therapists in Ontario: Impact on system integration and change** | Kennedy C.; Warmington K.; Soever L.; Passalent L.; Lineker S.; Thomas R.; Lundon K.; Shupak R.; Schneider R. | 2011 | Physiotherapy (United Kingdom) | Excluded | Exclusion reason: Not primary research; |
| **Feasibility and impact of a multi-component education intervention on improving self-efficacy to implement evidence-based practice among physiotherapists in stroke rehabilitation** | Salbach N.; Jaglal S.; Rappolt S.; Bayley M.; Burnett D.; Judd M.; Evans C. | 2011 | Physiotherapy (United Kingdom) | Excluded | Exclusion reason: Not primary research; |
| **The experiences of physiotherapists learning inter-professionally on a specialist rheumatology programme** | Ryan S.-J.; Wright J. | 2011 | Physiotherapy (United Kingdom) | Excluded | Exclusion reason: Not primary research; |
| **Clinically focused masters study: Moving towards clinical expertise** | Petty N. | 2011 | Physiotherapy (United Kingdom) | Excluded | Exclusion reason: Not primary research; |
| **Factors that influence development and integration of professional core values into physical therapy practice** | McGinnis P.; Guenther L.A.; Romen M.; Wainwright S. | 2011 | Physiotherapy (United Kingdom) | Excluded | Exclusion reason: Not primary research; |
| **Effects of an educational program for physical therapists to enhance the use of outcome measures in daily practice** | Custers J.; Swinkels R.; Van Peppen R.; Wittink H.; Schenkeveld C.; Beurskens S. | 2011 | Physiotherapy (United Kingdom) | Excluded | Exclusion reason: Not primary research; |
| **Advancing communication, assessment and clinical skills of physiotherapists through simulation: Outcomes of a state-wide education program for intensive care** | Thomas P.; Caruana L.; Chapman M.; Paratz J.; Rego P.; Schonfeldt-Roy S.; Tooth A.; Van Haeringen K. | 2010 | Anaesthesia and Intensive Care | Excluded | Exclusion reason: Not primary research |
| **Examination of clinician perceptions following continuing education on treatment-based classification for cervical pain** | Denninger T.D.; Whitman J.M. | 2010 | Journal of Manual and Manipulative Therapy | Excluded | Exclusion reason: Not primary research; |
| **A preliminary analysis of osteoporosis education and its effect on clinician treatment choices** | Joshi R.K.; Brolly M.; McArdle K.D.; Buckley J. | 2010 | Journal of Clinical Densitometry | Excluded | Exclusion reason: Not primary research; |
| **Learning to reason: A journey of professional socialisation** | Ajjawi R.; Higgs J. | 2008 | Advances in Health Sciences Education | Excluded | Exclusion reason: Wrong intervention; |
| **Knowledge in managing musculoskeletal conditions and educational preparation of physical therapists in the uniformed services** | Childs J.D.; Whitman J.M.; Pugia M.L.; Sizer Jr. P.S.; Flynn T.W.; Delitto A. | 2007 | Military Medicine | Excluded | Exclusion reason: Entry Level Education; |
| **Evidence based practice: a survey of physiotherapists' current practice** | Iles R.; Davidson M. | 2006 | Physiotherapy research international : the journal for researchers and clinicians in physical therapy | Excluded | Exclusion reason: Wrong intervention; |
| **Do physiotherapists' attitudes towards evidence-based practice change as a result of an evidence-based educational programme?** | Stevenson K.; Lewis M.; Hay E. | 2004 | Journal of Evaluation in Clinical Practice | Excluded | Exclusion reason: Wrong outcomes; |
| **Evaluation of an education program on the management of rheumatic diseases for physical therapists** | Stross J.K.; Banwell B.F.; Wolf F.M.; Becker M.C. | 1986 | Journal of Rheumatology | Excluded | Exclusion reason: Wrong outcomes; |
| **Physiotherapists' training in oncology rehabilitation from entry‐level to advanced education: A qualitative study.** | Bertoni, Gianluca; Conti, Valentina; Testa, Marco; Coppola, Ilaria; Costi, Stefania; Battista, Simone | 2024 | Physiotherapy Research International | Excluded | Exclusion reason: Wrong intervention; |
| **Musculoskeletal physiotherapists reasons for treatment selection and continuous professional development practices in the United Kingdom: A cross-sectional survey.** | Chesterton, Paul; Skidmore, Nathan | 2023 | Physiotherapy Practice & Research | Excluded | Exclusion reason: Wrong intervention; |
| **Elements of Learning and Integration of Diagnostic Musculoskeletal Ultrasound Imaging Into Practice: Physical Therapists' Educational Journeys.** | Hayward, Lorna M.; Markowski, Alycia; Watkins, Maureen K.; Maitland, Murray E.; Manske, Robert; Beneck, George J. | 2022 | Journal of Physical Therapy Education (Lippincott Williams & Wilkins) | Excluded | Exclusion reason: Wrong intervention; |
| **The professional development and career journey into musculoskeletal first contact physiotherapy: a telephone interview study.** | Bassett, Andrew Mark; Jackson, Jo | 2022 | Physiotherapy Theory & Practice | Excluded | Exclusion reason: Wrong intervention; |
| **Experiences of advanced clinical practitioners in training and their supervisors in primary care using a hub and spoke model.** | Gloster, Annabella Satu; Tomlins, Lucy; Murphy, Neil | 2020 | Practice Nursing | Excluded | Exclusion reason: Population is not exclusively PT |
| **LA COLLABORAZIONE DEL FISIOTERAPISTA DELL'AREA PEDIATRICA CON IL PEDIATRA DI FAMIGLIA: Studio osservazionale sullo stage formativo nel Master di Fisioterapia Pediatrica dell'Università degli Studi di Firenze a.a. 2006 - 2014.** | Scorpiniti, Silvia; Pierattelli, Monica; Paoli, Silvia; Davidson, Adrienne | 2019 | Scienza Riabilitativa | Excluded | Exclusion reason: Wrong outcomes; |
| **Physiotherapists Prefer Clinical Supervision to Focus on Professional Skill Development: A Qualitative Study.** | Snowdon, David A.; Cooke, Shae; Lawler, Katherine; Scroggie, Grant; Williams, Kimberley; Taylor, Nicholas F. | 2020 | Physiotherapy Canada | Excluded | Exclusion reason: Wrong outcomes; |
| **Implementation of a Hospital-Based Orthopaedic Physical Therapy Residency Program: A Case Report Describing Clinical Outcomes, Productivity, and Perceived Benefits.** | Winslow, John; Costello, Michael | 2019 | Journal of Physical Therapy Education (Lippincott Williams & Wilkins) | Excluded | Exclusion reason: Wrong Population; |
| **Employer Perceptions of Physical Therapists' Residency and Fellowship Training: Insights for Career Development Planning.** | Briggs, Matthew S.; Whitman, Julie; Olson-Kellogg, Becky; Farrell, Joseph; Glaws, Kathryn R.; Walker, Joann M.; Clutter, Jill; Tichenor, Carol Jo | 2019 | Journal of Physical Therapy Education (Lippincott Williams & Wilkins) | Excluded | Exclusion reason: Population is not exclusively PT |
| **How physiotherapists acquire management skills as they transition into a managerial role.** | Dallimore, Rachel Kim; Fiddler, Helen | 2018 | British Journal of Healthcare Management | Excluded | Exclusion reason: Wrong intervention; |
| **MACP e- portfolio platform: Proud to be providing a portfolio route for advanced practice.** |  | 2022 | Frontline (20454910) | Excluded | Exclusion reason: Not primary research; |
| **Light-touch mentorship of physiotherapists in face to face and digital consultations supports development of clinical expertise...Physiotherapy UK Virtual Conference, November 5-6, 2021** | McLean, L.; Johnson, W. |  | Physiotherapy | Excluded | Exclusion reason: Not primary research; |
| **A leadership development programme for regional clinical lead (RCL) physiotherapists: Evaluation of impact...Virtual Physiotherapy UK Conference, November 13-14, 2020.** | Gill, J.; Breach, J.; Davis, C. |  | Physiotherapy | Excluded | Exclusion reason: Not primary research; |
| **Building Passion Develops Meaningful Mentoring Relationships among Canadian Physiotherapists.** | Ezzat, Allison M.; Maly, Monica R. | 2012 | Physiotherapy Canada | Excluded | Exclusion reason: Population is not exclusively PT; |
| **Electronic mentoring: an innovative approach to providing clinical support...including commentary by Stewart S and Paterson M** | Stewart S; Carpenter C | 2009 | International Journal of Therapy & Rehabilitation | Excluded | Exclusion reason: Wrong outcomes; Kaitlyn Maddigan (2024-05-30 03:40:40) |
| **The evolution of a state-wide continuing education programme for allied health professionals...including commentary by White E** | Schoo AMM; Stagnitti KE; McNamara KP | 2008 | International Journal of Therapy & Rehabilitation | Excluded | Exclusion reason: Wrong intervention; |
| **The physical therapy practitioner: an expanded role for physical therapy in pediatric rheumatology.** | Campos AA; Graveline C; Ferguson JM; Lundon K; Schneider R; Laxer RM | 2001 | Physiotherapy Canada | Excluded | Exclusion reason: Not primary research; |
| **Developing clinical expertise through clinical placement at masters level.** | Rushton A; Lindsay G | 2007 | International Journal of Therapy & Rehabilitation | Excluded | Exclusion reason: Population is not exclusively PT; |
| **Professional doctorate: combining professional practice with scholarly inquiry.** | Morley M; Petty NJ | 2010 | British Journal of Occupational Therapy | Excluded | Exclusion reason: Population is not exclusively PT; |
| **Critical appraisal of research literature by expert and inexperienced physical therapy researchers.** | Domholdt E; Flaherty JL; Phillips JM | 1994 | Physical Therapy | Excluded | Exclusion reason: Wrong intervention |
| **The role of higher education in the career paths of board-certified clinical specialists in geriatric physical therapy: implications for professional and postprofessional education.** | Thompson M | 2001 | Journal of Physical Therapy Education (American Physical Therapy Association, Education Section) | Excluded | Exclusion reason: Wrong outcomes; |
| **The influence of experience and specialty certifications on clinical outcomes for patients with low back pain treated with a standardized physical therapy management program...including commentary by Resnik L with author response** | Whitman JM; Fritz JM; Childs JD | 2004 | Journal of Orthopaedic & Sports Physical Therapy | Excluded | Exclusion reason: Wrong intervention; Kaitlyn Maddigan (2024-04-29 03:27:18) |
| **Development, implementation, and evaluation of a continuing professional development module on physical therapists use of standardized balance measures: a knowledge translation study.** | Mowder-Tinney J | 2008 | Development, Implementation & Evaluation of a Continuing Professional Development Module on Physical Therapists Use of Standardized Balance Measures: A Knowledge Translation Study | Excluded | Exclusion reason: Population is not exclusively PT |
| **Development and Early Evaluation of an Inter-professional Post-licensure Education Programme for Extended Practice Roles in Arthritis Care** | Lundon, K; Shupak, R; Schneider, R; McIlroy, JH | 2011 | PHYSIOTHERAPY CANADA | Excluded | Exclusion reason: Population is not exclusively PT; |
| **No pain no gain: critical features of peer assessment to improve compliance with guideline recommendations in physical therapy** | Maas, M; Van Dulmen, SA; Van Der Wees, PJ; Heerkens, YF; Van Der Vleuten, CP; Nijhuis-Van Der Sanden, MWG | 2015 |  | Excluded | Exclusion reason: Not primary research; |
| **Does online professional development for physiotherapists enhance clinical practice and patient outcomes? A mixed methods evaluation** | ACTRN12622000123741, | 2022 |  | Excluded | Exclusion reason: Not primary research; |
| **Building workforce capacity in management of rheumatoid arthritis: a single-blind, parallel, randomised controlled trial of web-based e-learning for physiotherapists to improv eknowledge and skills for the management of rheumatoid arthritis in community** | Fary, R; Slater, H; Chua, J; Ranelli, S; Chan, M; Briggs, A | 2014 |  | Excluded | Exclusion reason: Not primary research; |
| **HWA Expanded Scopes of Practice program evaluation: Physiotherapists in the Emergency Department sub-projec:t final report** | Thompson, Cristina; Williams, Kate; Morris, Darcy; Bird, Sonia; Kobel, Conrad; Andersen, Patrea; Eckermann, Simon; Quinsey, Karen; Masso, Malcolm |  |  | Excluded | Exclusion reason: Not primary research; |
| **Communication Skills Training for Practitioners to Increase Patient Adherence to Home-Based Rehabilitation for Chronic Low Back Pain: results of a Cluster Randomized Controlled Trial** | Lonsdale, C; Hall, AM; Murray, A; Williams, GC; McDonough, SM; Ntoumanis, N; Owen, K; Schwarzer, R; Parker, P; Kolt, GS; et al. | 2017 |  | Included |  |
| **A structured workshop enhanced Physiotherapists' skills in clinical decision-making: A pre-post study.** | Adhikari, Shambhu P; Shrestha, Nistha; Shakya, Rishita; Phuyal, Rajani; Gyawali, Manju; Dev, Rubee | 2020 | Journal of family medicine and primary care | Included |  |
| **Effects of an Online Education Program on Physical Therapists' Confidence in Weight Management for People With Osteoarthritis: A Randomized Controlled Trial** | Allison K.; Jones S.; Hinman R.S.; Briggs A.M.; Sumithran P.; Quicke J.; Holden M.; Chiavaroli N.; Crofts S.; George E.; Foster N.; Bennell K. | 2023 | Arthritis Care and Research | Included |  |
| **Der aktuelle Stand der Employability (Beschäftigungsfähigkeit) von OMT-Absolventen der muskuloskelettalen (manuellen) Therapie in Deutschland.** | Anderseck, Jana; Lüning, Eva; von Piekartz, Harry | 2020 | Manuelle Therapie | Included |  |
| **Effects of a Customized Professionalism Educational Intervention on Physical Therapists' Knowledge and Attributes of Professionalism.** | Balogun, Joseph A.; Mbada, Chidozie E.; Balogun, Adetutu O.; Okafor, Udoka A. | 2018 | Internet Journal of Allied Health Sciences & Practice | Included |  |
| **Do the clinical competencies of musculoskeletal outpatient physiotherapists improve after they have participated in a bespoke in-service education programme designed around individual and service continuing professional development needs?.** | Banks, Kevin; Meaburn, Anthony; Phelan, Elaine | 2013 | Journal of allied health | Included |  |
| **Program evaluation of GLA:D Australia: Physiotherapist training outcomes and effectiveness of implementation for people with knee osteoarthritis** | Barton C.J.; Kemp J.L.; Roos E.M.; Skou S.T.; Dundules K.; Pazzinatto M.F.; Francis M.; Lannin N.A.; Wallis J.A.; Crossley K.M. | 2021 | Osteoarthritis and Cartilage Open | Included |  |
| **Postgraduate clinical physiotherapy education in acute hospitals: a cohort study** | Bastick E.K.; O'Keeffe D.D.; Farlie M.K.; Ryan D.T.; Haines T.P.; Katz N.; Knight J.L.; Keely L.K.; Saber K.J.; Sturgess T.R.; Skinner E.H. | 2020 | Physiotherapy theory and practice | Included |  |
| **Training physiotherapists to be responsive to their clients' health literacy needs.** | Bird, Marie-Louise; Elmer, Shandell; Osborne, Richard H; Flittner, Anna; O'Brien, Jane | 2022 | Physiotherapy theory and practice | Included |  |
| **Impact of continuing education interventions on clinical outcomes of patients with neck pain who received physical therapy.** | Brennan, Gerard P; Fritz, Julie M; Hunter, Stephen J | 2006 | Physical therapy | Included |  |
| **Comparisons of Clinical Competency and Job Responsibilities of Physical Therapists With and Without Postprofessional Training.** | Briggs, Matthew S.; Gulla, Nicholas; Howald, Heidi; Weber, Mark D.; Olson-Kellogg, Becky J.; DeWitt, John J.; Hensley, Craig P.; Harrington, Kendra L.; Kidder, Melissa S.; Farrell, Joseph P.; Tichenor, Carol Jo | 2023 | Journal of Physical Therapy Education (Lippincott Williams & Wilkins) | Included |  |
| **Knowledge to Practice in Developmental Coordination Disorder: Impact of an Evidence-Based Online Module on Physical Therapists' Self-Reported Knowledge, Skills, and Practice.** | Camden, Chantal; Rivard, Lisa; Pollock, Nancy; Missiuna, Cheryl | 2015 | Physical & Occupational Therapy in Pediatrics | Included |  |
| **Developing clinical expertise in musculoskeletal physiotherapy; Using observed practice to create a valued practice-based collaborative learning cycle** | Carr M.; Morris J.; Kersten P. | 2020 | Musculoskeletal Science and Practice | Included |  |
| **The effectiveness of post‐professional physical therapist training in the treatment of chronic low back pain using a propensity score approach with machine learning.** | Cheema, Carolyn; Baldwin, Jonathan; Rodeghero, Jason; Werneke, Mark W.; Mioduski, Jerry E.; Jeffries, Lynn; Kucksdorf, Joseph; Shepherd, Mark; Randall, Ken; Dionne, Carol | 2022 | Musculoskeletal Care | Included |  |
| **Can a professional development workshop with follow-up alter practitioner behaviour and outcomes for neck pain patients? A randomised controlled trial.** | Chipchase, L S; Cavaleri, R; Jull, G | 2016 | Manual therapy | Included |  |
| **Evidence-Based Practice (EBP) in Rehabilitative Physiotherapy.** | Cimoli C. | 2012 | Internet Journal of Allied Health Sciences & Practice | Included |  |
| **Does continuing education improve physical therapists effectiveness in treating neck pain: a randomized clinical trial.** | Cleland J; Fritz J; Brennan GP; Magel JS |  | Journal of Orthopaedic & Sports Physical Therapy | Included |  |
| **Bringing Masters' level skills to the clinical setting: what is the experience like for graduates of the Master of Science in manual therapy programme?.** | Constantine, Michael; Carpenter, Christine | 2012 | Physiotherapy theory and practice | Included |  |
| **The perspectives of physiotherapists on managing nonspecific low back pain following a training programme in cognitive functional therapy: A qualitative study** | Cowell I.; O'Sullivan P.; O'Sullivan K.; Poyton R.; McGregor A.; Murtagh G. | 2019 | Musculoskeletal care | Included |  |
| **The Clinical Influence of a Collaborative Partnership for Physical Therapy Residency Training in Kenya: Perspectives of Graduates and their Employers.** | Cunningham, Shala; Jackson, Richard; Herbel, Ken | 2021 | Orthopaedic Physical Therapy Practice | Included |  |
| **Professional development and lifelong learning: the impact of physical therapy residency training in Kenya.** | Cunningham, Shala; Litwin, Bini; Fernandez-Fernandez, Alicia; Canbek, Jennifer | 2022 | Physiotherapy theory and practice | Included |  |
| **Influence of residency training on the clinical reasoning development of Kenyan physiotherapists.** | Cunningham, Shala; Litwin, Bini; Fernandez-Fernandez, Alicia; Canbek, Jennifer | 2019 | The Journal of manual & manipulative therapy | Included |  |
| **The Influence of an Orthopedic, Manual Therapy Residency Program on Improved Knowledge, Psychomotor Skills, and Clinical Reasoning in Nairobi, Kenya** | Cunningham, Shala; McFelea, Joni | 2017 | Frontiers in Public Health | Included |  |
| **Tailored skills training for practitioners to enhance assessment of prognostic factors for persistent and disabling back pain: four quasi-experimental single-subject studies.** | Demmelmaier, Ingrid; Denison, Eva; Lindberg, Per; Asenlof, Pernilla | 2012 | Physiotherapy theory and practice | Included |  |
| **What physiotherapists in private practice do: the effects of sex and training on clinical behaviour** | Dennis J.K. | 1987 | Australian Journal of Physiotherapy | Included |  |
| **Physical therapists' level of McKenzie education, functional outcomes, and utilization in patients with low back pain.** | Deutscher, Daniel; Werneke, Mark W; Gottlieb, Ditza; Fritz, Julie M; Resnik, Linda | 2014 | The Journal of orthopaedic and sports physical therapy | Included |  |
| **Effectiveness of the tailored Evidence Based Practice training program for Filipino physical therapists: a randomized controlled trial** | Dizon J.M.; Grimmer-Somers K.; Kumar S. | 2014 | BMC medical education | Included |  |
| **Policy-into-practice for rheumatoid arthritis: Randomized controlled trial and cohort study of E-learning targeting improved physiotherapy management** | Fary R.E.; Slater H.; Chua J.; Ranelli S.; Chan M.; Briggs A.M. | 2015 | Arthritis Care and Research | Included |  |
| **Clinical narratives in residency education: Exploration of the learning process.** | Furze, Jennifer A; Greenfield, Bruce H; Barr, J Bradley; Geist, Kathleen; Gale, Judith; Jensen, Gail M | 2019 | Physiotherapy theory and practice | Included |  |
| **The influence of a postgraduate clinical master's qualification in manual therapy on the careers of physiotherapists in the United Kingdom.** | Green, Ann; Perry, Jo; Harrison, Karen | 2008 | Manual therapy | Included |  |
| **Barriers and facilitators to achieving competence in lung ultrasound: A survey of physiotherapists following a lung ultrasound training course** | Hansell L.; Milross M.; Delaney A.; Tian D.H.; Rajamani A.; Ntoumenopoulos G. | 2023 | Australian critical care : official journal of the Confederation of Australian Critical Care Nurses | Included |  |
| **Knowledge, skills and barriers to evidence-based practice and the impact of a flipped classroom training program for physical therapists: An observational study** | Harrison L.; Wong D.; Traeger A.C.; Harmer A.R.; Jennings M.; Moseley A.M. | 2022 | Physiotherapy theory and practice | Included |  |
| **Telehealth e-mentoring in postgraduate musculoskeletal physiotherapy education: A mixed methods case study to inform implementation for advanced clinical practice** | Heneghan N.R.; Jagodzinska J.; Tyros I.; Johnson W.; Nazareth M.; Yeung E.; Gillis H.; Sadi J.; Rushton A. | 2022 | Physiotherapy (United Kingdom) | Included |  |
| **A Comparison of Professional Development and Leadership Activities Between Graduates and Non-graduates of Physical Therapist Clinical Residency Programs** | Jones, Stephanie; Bellah, Chuck; Godges, Joseph J. | 2008 | Journal of Physical Therapy Education | Included |  |
| **Assessing the impact of a knowledge translation intervention on physical therapists' self-efficacy and implementation of motor learning practice.** | Kafri, Michal; Levron, Yasmin; Atun-Einy, Osnat | 2023 | BMC medical education | Included |  |
| **A Knowledge Translation Programme to Increase the Utilization of Thoracic Spine Mobilization and Manipulation for Patients with Neck Pain.** | Karas, Steve; Westerheide, Angela; Daniel, Laura | 2016 | Musculoskeletal care | Included |  |
| **Knowledge translation from continuing education to physiotherapy practice in classifying patients with low back pain** | Karvonen E.; Paatelma M.; Kesonen J.-P.; Heinonen A.O. | 2015 | Journal of Manual and Manipulative Therapy | Included |  |
| **Educating patient educators: enhancing instructional effectiveness in physical therapy for low back pain patients.** | Kerssens, J J; Sluijs, E M; Verhaak, P F; Knibbe, H J; Hermans, I M | 1999 | Patient education and counseling | Included |  |
| **Online Education Improves Confidence in Mechanical Insufflation-Exsufflation** | Lambrinos E.; Elkins M.R.; Menadue C.; McGuiness O.A.; Melehan K.L.; Piper A.J. | 2023 | Respiratory care | Included |  |
| **Effectiveness of training physical therapists in pain neuroscience education for patients with chronic spine pain: A cluster-randomized trial** | Lane E.; Magel J.S.; Thackeray A.; Greene T.; Fino N.F.; Puentedura E.J.; Louw A.; Maddox D.; Fritz J.M. | 2022 | Pain | Included |  |
| **Implementation of person-centred practice principles and behaviour change techniques after a 2-day training workshop: A nested case study involving physiotherapists** | Lawford B.J.; Bennell K.L.; Kasza J.; Campbell P.K.; Gale J.; Bills C.; Hinman R.S. | 2019 | Musculoskeletal care | Included |  |
| **Training Physical Therapists in Person-Centered Practice for People With Osteoarthritis: A Qualitative Case Study.** | Lawford, Belinda J; Delany, Clare; Bennell, Kim L; Bills, Caroline; Gale, Janette; Hinman, Rana S | 2018 | Arthritis care & research | Included |  |
| **Effects of physical therapist training on outcomes of patients with chronic low back pain or chronic shoulder pain** | Levsen M.J.; Hansen M.L.; Kent A.D.; Sieren J.J.; Thoreson J.P.; Farrell K.P. | 2001 | Journal of Manual and Manipulative Therapy | Included |  |
| **The clinical impact of pain neuroscience continuing education on physical therapy outcomes for patients with low back and neck pain.** | Louw, Adriaan; Puentedura, Emilio J; Denninger, Thomas R; Lutz, Adam D; Cox, Terry; Zimney, Kory; Landers, Merrill R | 2022 | PloS one | Included |  |
| **Perceptions of the professional and personal impact of hybrid fellowship training: a qualitative study** | MacPherson, Kevin L.; Shepherd, Mark; Everett, Jamie Childs; Fritsch, Adam; Whitman, J.M.; Dunleavy, Kim | 2019 | J Man Manip Ther | Included |  |
| **Investigating the impact of postgraduate musculoskeletal physiotherapy education on practitioners' clinical reasoning skills** | Madi, Mohammad Abdelfattah Atallah | 2018 | PQDT - UK & Ireland | Included |  |
| **An exploratory study considering the potential impacts of high-fidelity simulation based education on self-evaluated confidence of non-respiratory physiotherapists providing an on-call respiratory physiotherapy service: a mixed methods study** | Mansell, Stephanie K; Harvey, Alex; Thomas, Amanda | 2020 | BMJ Simul Technol Enhanc Learn | Included |  |
| **Improving musculoskeletal physiotherapists' confidence in patient-centred care informed by acceptance and commitment therapy: A descriptive study.** | March, Marie K; Judd, Belinda; Harmer, Alison R; Eyles, Jillian; Dennis, Sarah M | 2024 | Musculoskeletal science & practice | Included |  |
| **Effect of a self-determination theory-based communication skills training program on physiotherapists' psychological support for their patients with chronic low back pain: a randomized controlled trial.** | Murray, Aileen; Hall, Amanda M; Williams, Geoffrey C; McDonough, Suzanne M; Ntoumanis, Nikos; Taylor, Ian M; Jackson, Ben; Matthews, James; Hurley, Deirdre A; Lonsdale, Chris | 2015 | Archives of physical medicine and rehabilitation | Included |  |
| **Narrative analysis to track the development of clinical reasoning during residency** | Naidoo, Keshrie; Baldwin, Jane; Lesar, Jessie; Plummer, Laura | 2022 | The Journal of Clinical Education in Physical Therapy | Included |  |
| **Evaluation of a pilot programme on diagnostic thoracic ultrasound curriculum for acute care physiotherapists** | Ntoumenopoulos G.; Ong H.K.; Toh H.C.; Saclolo R.P.; Sewa W.D. | 2017 | Australasian Journal of Ultrasound in Medicine | Included |  |
| **An innovative peer assessment program to improve evidence based practice in physical therapy** | Marjo J.M. Maas, Philip J. van der Wees, Carla Braam, Jan Koetsenruijter, Yvonne F. Heerkens, Cees P.M. van der Vleuten, Maria W.G. Nijhuis-van der Sanden | 2012 |  | Included |  |
| **Impact of a multifaceted and clinically integrated training program in evidence-based practice on knowledge, skills, beliefs and behaviour among clinical instructors in physiotherapy: A non-randomized controlled study** | Olsen N.R.; Bradley P.; Espehaug B.; Nortvedt M.W.; Lygren H.; Frisk B.; Bjordal J.M. | 2015 | PLoS ONE | Included |  |
| **Do physical therapists change their beliefs, attitudes, knowledge, skills and behaviour after a biopsychosocially orientated university course?** | Overmeer T; Boersma K; Main CJ; Linton SJ | 2009 | Journal of Evaluation in Clinical Practice | Included |  |
| **Does teaching physical therapists to deliver a biopsychosocial treatment program result in better patient outcomes? A randomized controlled trial.** | Overmeer, Thomas; Boersma, Katja; Denison, Eva; Linton, Steven J | 2011 | Physical therapy | Included |  |
| **The impact of Masters education in manual and manipulative therapy and the 'knowledge acquisition model'.** | Perry, Jo; Green, Ann; Harrison, Karen | 2011 | Manual therapy | Included |  |
| **Postgraduate education to increase adherence to a Dutch physiotherapy practice guideline for hip and knee OA: a randomized controlled trial.** | Peter, Wilfred F; van der Wees, Philip J; Verhoef, John; de Jong, Zuzana; van Bodegom-Vos, Leti; Hilberdink, Wim K H A; Fiocco, Marta; Vliet Vlieland, Thea P M | 2013 | Rheumatology (Oxford, England) | Included |  |
| **Effectiveness of an interactive postgraduate educational intervention with patient participation on the adherence to a physiotherapy guideline for hip and knee osteoarthritis: a randomised controlled trial.** | Peter, Wilfred; van der Wees, Philip J; Verhoef, John; de Jong, Zusana; van Bodegom-Vos, Leti; Hilberdink, Wim K H A; Fiocco, Marta; Vliet Vlieland, Thea P M | 2015 | Disability and rehabilitation | Included |  |
| **Master's level study: learning transitions towards clinical expertise in physiotherapy.** | Petty, Nicola J; Scholes, Julie; Ellis, Lorraine | 2011 | Physiotherapy | Included |  |
| **The impact of a musculoskeletal masters course: developing clinical expertise.** | Petty, Nicola J; Scholes, Julie; Ellis, Lorraine | 2011 | Manual therapy | Included |  |
| **The Impact of Coaching in Physical Therapy Residency on the Mentor and Resident for the Development of Clinical Reflection and Reasoning Skills** | Prizinski, Francois Andre | 2021 | ProQuest Dissertations and Theses | Included |  |
| **Evaluating two implementation strategies for whiplash guidelines in physiotherapy: a cluster randomised trial.** | Rebbeck, Trudy; Maher, Christopher G; Refshauge, Kathryn M | 2006 | The Australian journal of physiotherapy | Included |  |
| **Influence of Advanced Orthopaedic Certification on Clinical Outcomes of Patients with Low Back Pain** | Resnik L.; Hart D.L. | 2004 | Journal of Manual and Manipulative Therapy | Included |  |
| **Evaluation of an educational course for primary care physiotherapists on comorbidity‐adapted exercise therapy in knee osteoarthritis: an observational study.** | De Rooij, Mariëtte; Leeden, Marike; Esch, Martin; Lems, Willem F.; Meesters, Jorit J.L.; Peter, Wilfred F.; Roorda, Leo D.; Terbraak, Michel S.; Vredeveld, Tom; Vliet Vlieland, Thea P.M.; Dekker, Joost | 2020 | Musculoskeletal Care | Included |  |
| **Defining the construct of masters level clinical practice in manipulative physiotherapy.** | Rushton, Alison; Lindsay, Geoff | 2010 | Manual therapy | Included |  |
| **The effect of knowledge translation procedures on application of information from a continuing education conference.** | Schreiber, Joseph; Dole, Robin L | 2012 | Pediatric physical therapy : the official publication of the Section on Pediatrics of the American Physical Therapy Association | Included |  |
| **Transferring New Physical Therapy Skills from the Weekend to Monday Morning in the Clinic: A Pilot Study.** | Seif, Gretchen A.; Faris, Katie; Russo, Gabriella; Middleton, Addie; Timko, Michael | 2019 | Journal of Allied Health | Included |  |
| **Satisfaction and attitudes towards online continuous medical education and its impact on clinical practice among physiotherapists.** | Shalabi, Kholood Matouq; Almurdi, Muneera Mohammed | 2024 | BMC medical education | Included |  |
| **Integrating theory and practice by self-directed inquiry-based learning? A pilot study** | Sjodahl Hammarlund C.; Nordmark E.; Gummesson C. | 2013 | European Journal of Physiotherapy | Included |  |
| **Orthopaedic residency training: a survey of the graduates' perspective.** | Smith, K L; Tichenor, C J; Schroeder, M | 1999 | The Journal of orthopaedic and sports physical therapy | Included |  |
| **Educational and Experiential Factors Associated With Physical Therapists' Diagnostic Reasoning.** | Souter, Courtney; Musy, Emily; Hartstein, Aaron; Lievre, Arthur; Fergus, Andrea | 2019 | Journal of Physical Therapy Education (Lippincott Williams & Wilkins) | Included |  |
| **Study at master's level by practising physiotherapists** | Stathopoulos I.; Harrison K. | 2003 | Physiotherapy | Included |  |
| **Development, implementation and evaluation of a bespoke, advanced practice musculoskeletal training programme within a clinical assessment and treatment service.** | Stevenson, Kay; Bicker, Greg; Cliffe, Stephanie; Kemp, John; Menon, Ajit; Hall, Emma; Ryan, Sarah | 2020 | Musculoskeletal Care | Included |  |
| **Does physiotherapy management of low back pain change as a result of an evidence-based educational programme?.** | Stevenson, Kay; Lewis, Martyn; Hay, Elaine | 2006 | Journal of evaluation in clinical practice | Included |  |
| **Using Outcome Measures in Daily Practice: Development and Evaluation of an Implementation Strategy for Physiotherapists in the Netherlands** | Swinkels, Raymond; Meerhoff, Guus; Custers, Jan; Peppen, Roland; Beurskens, Anna; Wittink, Harriet | 2015 | Physiotherapy Canada | Included |  |
| **Physiotherapists report improved understanding of and attitude toward the cognitive, psychological and social dimensions of chronic low back pain after Cognitive Functional Therapy training: a qualitative study** | Synnott A.; O'Keeffe M.; Bunzli S.; Dankaerts W.; O'Sullivan P.; Robinson K.; O'Sullivan K. | 2016 | Journal of physiotherapy | Included |  |
| **Promoting physical therapists' use of research evidence to inform clinical practice: part 2--a mixed methods evaluation of the PEAK program** | Tilson J.K.; Mickan S.; Sum J.C.; Zibell M.; Dylla J.M.; Howard R. | 2014 | BMC medical education | Included |  |
| **A Method Model Presentation: Online Clinical Mentoring for Physical Therapists Experiencing Professional Isolation.** | Westervelt, Karen C.; Kunker, Katrina; Patel, Radha; Smith, Elizabeth; Wolitzer, Kaylee | 2020 | Journal of Physical Therapy Education (Lippincott Williams & Wilkins) | Included |  |
| **An orthopedic manual physical therapy fellowship training’s impact on professional development, involvement, personal lives, and income – A survey study** | Whitman, Julie M.; Shepherd, Mark; Neilson, Brett; Janicky, T. J.; Garcia, William J.; Peterson, Seth; Stevens, Barbara J. |  | J Man Manip Ther | Included |  |
| **Evaluation of the clinical effectiveness of a work-based mentoring programme to develop clinical reasoning on patient outcome: A stepped wedge cluster randomised controlled trial.** | Williams, Aled; Rushton, Alison; Lewis, James J; Phillips, Ceri | 2019 | PloS one | Included |  |
| **The Impact of Physical Therapy Residency or Fellowship Education on Clinical Outcomes for Patients With Musculoskeletal Conditions** | Rodeghero J, Wang YC, Flynn T, Cleland JA, Wainner RS, Whitman JM | 2015 | J Orthop Sports Phys Ther | Included |  |
